# MoS_2_-Catalyzed transamidation reaction

**DOI:** 10.1038/s41598-019-39210-5

**Published:** 2019-02-22

**Authors:** Feng Zhang, Lesong Li, Juan Ma, Hang Gong

**Affiliations:** 1 College of Science, Hunan Agricultural University, Changsha, 410128 China; 2 The Key Laboratory of Environmentally Friendly Chemistry and Application of the Ministry of Education, College of Chemistry, Xiangtan University, Xiangtan, 411105 China

## Abstract

The MoS_2_-catalyzed transamidation reaction with high yields using *N*,*N*-dimethylformamide and other amides as carbonyl sources is developed here. The protocol is simple, does not require any additive such as acid, base, ligand, etc., and encompasses a broad substrate scope for primary, secondary and heterocyclic amines. Moreover, the acetylation and propanylation of amines also can be achieved with good to excellent yield by this strategy.

## Introduction

**Figure 1:**
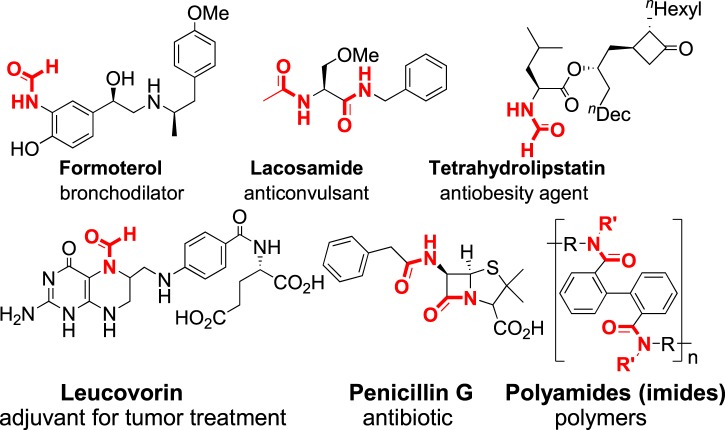
Amide-containing biologically active molecules and polymer.

Transamidation reaction is very important in organic synthesis chemistry^1–6^. And the amide framework is widely applied in medicines^7–10^, natural products^11^, and functional materials (Fig. 1)^12^. Several organic small molecules have been served as carbonyl sources in transamidation reactions, including DMF/DMA^13–24^, formic acid/formate^25–31^, methanol^32–36^, ester^37–41^, and others^42,43^. Among them, DMF is well known as a cheap and readily available industrial organic solvent. Additionally, DMF has also been widely applied as a source of dimethylamino, formyl, carbonyl, −CONMe_2_, and methyl^44–48^.

The catalyzed transamidation reactions using DMF as carbonyl source have been reported. Various catalysts, including metal catalysts, such as Ni^13^, Ce^14^, Fe^15^, Cu^16^, L-Proline^17^, Pd^49^ and metal-free catalysts, such as boronic acid^18^ and their derivatives^19,20^, and imidazole^50^ and its derivatives^21^ have been succeed in this transformation. However, these strategies are often suffered from drawbacks such as use of unreadily available and costly catalysts, large amount of catalyst, essential additives, limited scope, and etc.

**Figure 2:**
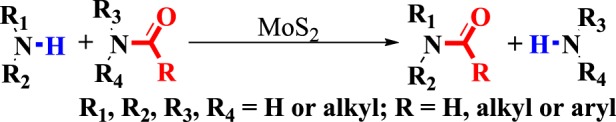
MoS_2_-catalyzed transamidation reaction.

Herein, a highly efficient MoS_2_-catalyzed transamidation reaction using DMF and other amides as carbonyl sources has been developed (Fig. 2). This method has advantages such as using inexpensive catalyst and reagents, no need for any other additives. Moreover, this strategy has a broad substrate scope that both primary and secondary amines with different groups are suitable for this reaction and good to excellent yields can be achieved. Particularly, acetylation and propanylation reaction using the corresponding amides as reagents could be achieved with almost the same good results as formylation reaction.

## Results and Discussion

**Figure 3:**
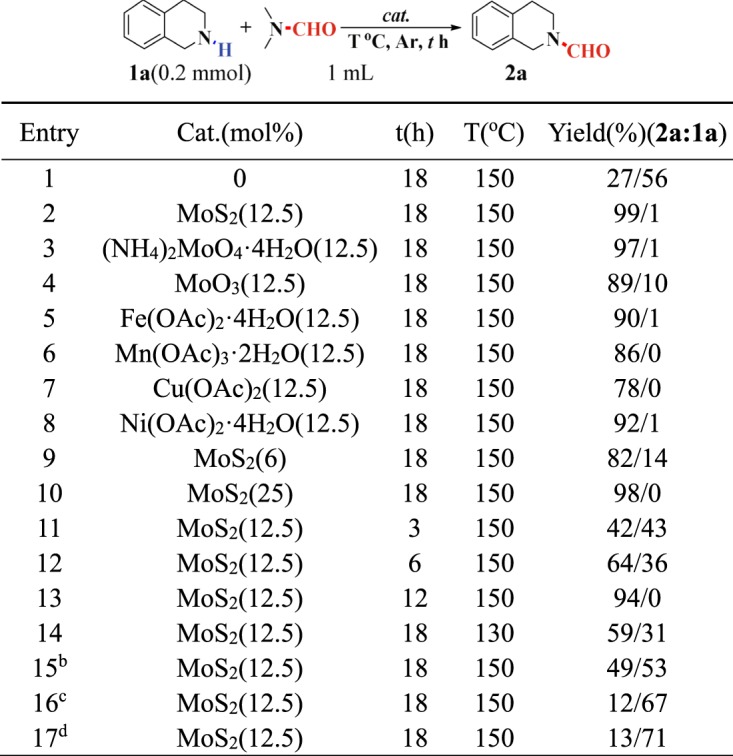
Selected optimization results. ^a^Unless otherwise noted, all reactions were conducted at 0.2 mmol scale in a sealed tube in 1.0 mL DMF under argon atmosphere. Yields are detected by 1 H NMR using CH_3_NO_2_ as internal standard; ^b^Under air atmosphere; ^c^1 mL toluene and 1.5 equiv. DMF were used; ^d^1 mL dioxane and 1.5 equiv. DMF were used.

Initially, the transamidation of tetrahydroisoquinoline and DMF catalyzed by MoS_2_ was investigated as the model reaction (Fig. 3). Various reaction conditions were optimized, such as catalyst, temperature, reaction time, and atmosphere. The results showed the yield of desired product is very poor in the absence of Mo catalyst (entry 1). When MoS_2_ (12.5 mol%) and (NH_4_)_2_MoO_4_·4H_2_O (12.5 mol%) were used as catalyst, an excellent yield of 99% and 97% can be obtained, respectively (entries 2 and 3). Afterward, other metal salts, such as Fe(OAc)_2_·4H_2_O, Mn(OAc)_3_·2H_2_O, Cu(OAc)_2_, and Ni(OAc)_2_·4H_2_O were investigated as catalysts. Satisfactory yields could also be achieved, but none of these catalyst worked better than MoS_2_. Therefore, MoS_2_ was selected as the optimal catalyst (entries 5–8). This reaction can be completed within 18 hours (entries 1, 11–13). Decreasing the reaction temperature (entry 14) proved to be unfavorable to this transformation. Additionally, the presence of air was harmful to this reaction (entry 15). Finally, this reaction was conducted by using stoichiometric amounts of DMF (1.5 equiv.) as the reagent in another solvent, but only poor yield was obtained (entries 16–17).

**Figure 4:**
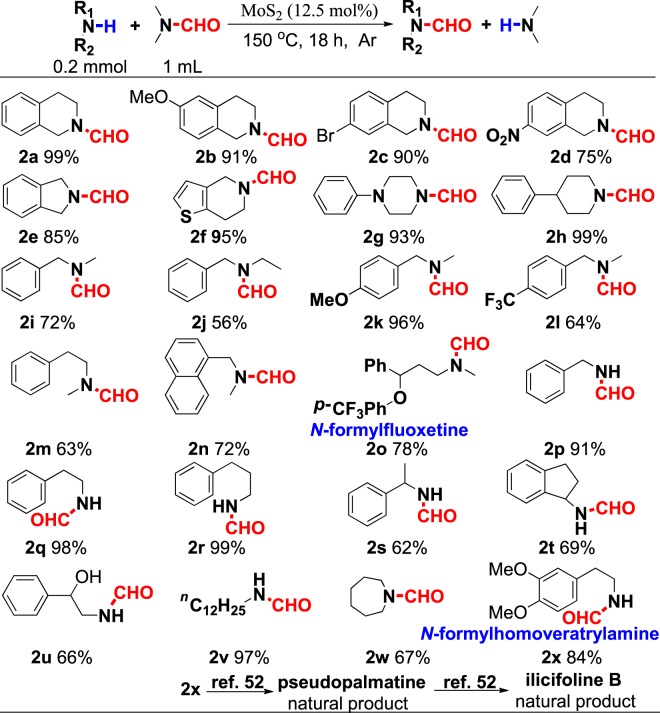
Substrate scope of amines. ^a^Unless otherwise noted, all reactions were conducted on a 0.2 mmol scale, DMF (1.0 mL), MoS_2_ (12.5 mol%) in a sealed tube under an atmosphere of argon for 18 h. Isolated yield was given.

With the optimized condition in hand, various amines, including primary, secondary and heterocyclic amines, were tested for this reaction (Fig. 4). The results indicated that tetrahydroisoquinoline or its analogs could be converted to desired product with good to excellent yields (**2a–f**). This reaction was tolerable for various functional groups such as methoxy, bromide, and nitro. Notably, the electron-withdrawing group is unfavorable to this reaction (**2d**), and a lower yield would be found. In the case of heterocyclic amines, this transformation proceeded well and the transamidation products were obtained with excellent yields (**2g–h**). Other secondary amines, either circular or linear amines, underwent this reaction smoothly with high yields in most cases (**2i–o**). Remarkably, compound **2o** which containing trifluoromethyl group, a marker for fluoxetine^51^, could be synthesized by this strategy with good yield (78%). However, the substrate with obvious steric hindrance group, such as ethyl (**1j**) on nitrogen was suffered from a reduced yield. This transamidation strategy was also applicable to primary amines, and good results as well as secondary amines were achieved (**2p–v**). Particularly, substrate containing hydroxyl group also converted to desired product with good yield (**2** **u**). The transamidation reaction and the corresponding product are valuable in organic synthesis reaction. For example, the natural product homoveratrylamine (**1x**) can be modified with formyl group by this strategy, and the corresponding product **2x** could be converted to a series of natural compounds such as pseudopalmatine, 8-oxopseudopalmatine, and ilicifoline B^52^. However, when aromatic amines such as aniline, tetrahydroquinoline and tetrahydroindole were used as substrates, only trace amount of desired products could be detected.

**Figure 5:**
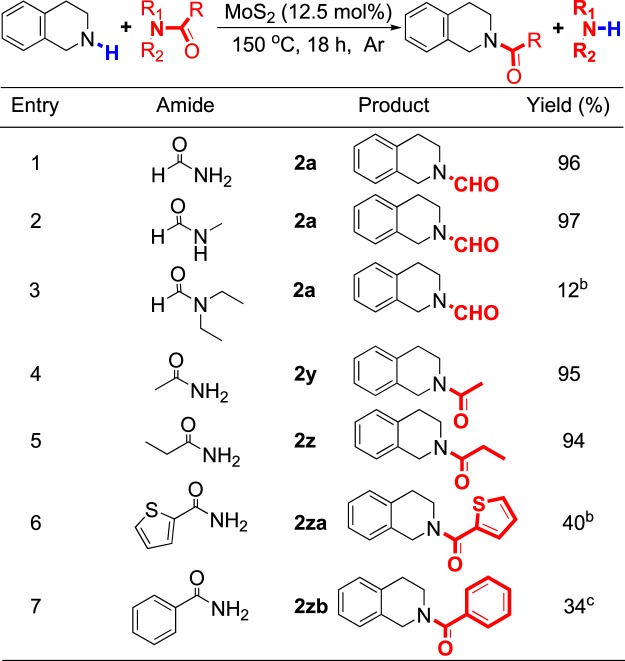
Substrate scope of amides. ^a^Unless otherwise noted, all reactions were conducted on a 0.2 mmol scale in a sealed tube in 1.0 mL amide under an atmosphere of argon for 18 h. Isolated yield was given; ^b^Yield is detected by ^1^H NMR using CH_3_NO_2_ as internal standard; ^c^From 2-thiophenecarboxamide 0.5 g, ethylene glycol 0.5 mL, reaction time is 24 h; ^d^From benzamide 0.5 g, ethylene glycol 0.5 mL, reaction time is 24 h.

After the expanded substrate scope of amines, we then considered other appropriate carbonyl sources except DMF (Fig. 5). The results showed this reaction could also be proceeded with excellent yield by using formamide or *N*-methylformamide as formyl sources (entries 1–2). However, in the case of using sterically hindered amide such as *N*,*N*-diethylformamide as substrate, the yield would be reduced seriously (entry 3). Delightedly, acetamide and propionamide could be applied as carbonyl sources, and the corresponding *N*-acetylation and *N*-propionylation reaction were achieved with excellent yields (entries 4–5). These results further confirmed the transamidation strategy developed by us has a broad scope and is valuable. Moreover, in the case of aromatic amides used as carbonyl sources, a yield of 30~40% still could be found (entries 6–7).

**Figure 6:**
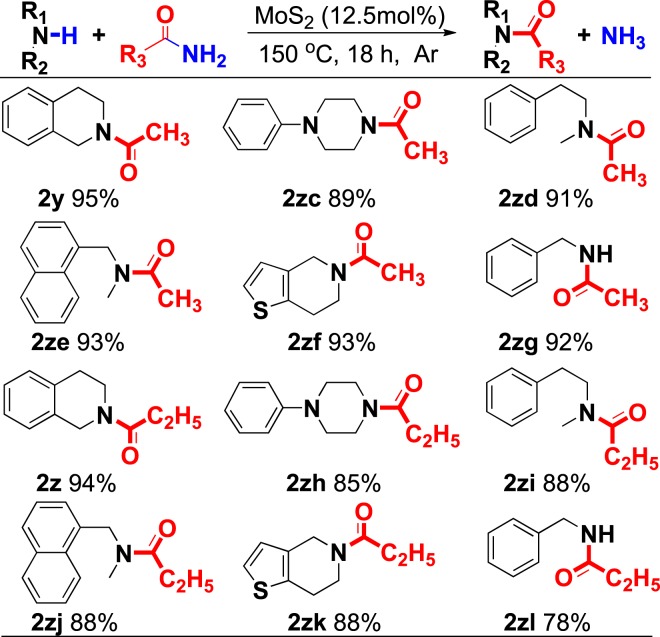
Substrate scope of acetylation and propanylation of amines. ^a^Unless otherwise noted, all reactions were conducted on a 0.2 mmol scale, acetamide or propionamide (1.0 g), MoS_2_ (12.5 mol%) in a sealed tube under an atmosphere of argon for 18 h. Isolated yield was given.

To verification the practicability of this strategy, the *N*-acetylation and *N*-propionylation reaction were expanded (Fig. 6). Either primary amines or secondary amines were converted to corresponding products with good to excellent yields (**2zc-zl**).

**Figure 7:**
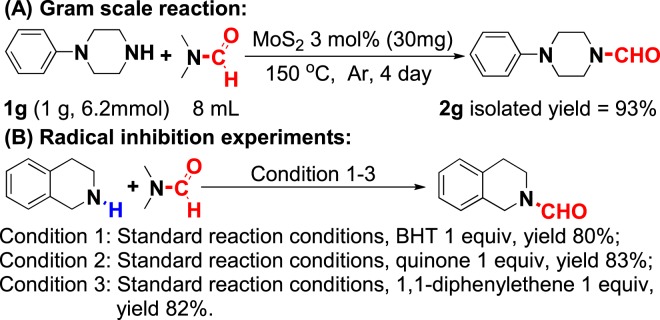
Gram reaction of 1 g and radical blocking experiments of **1a**.

Significantly, the gram-scale synthesis of **2g** using only 3 mol% MoS_2_ as catalyst was proceeded (Fig. 7A), and an excellent yield of 93% was obtained with an extended reaction time (4 days). Subsequently, the radical blocking experiments were performed using 1 equiv butylated hydroxytoluene (BHT), quinone, or 1,1-diphenylethene as a blocker, and the transamidation products were achieved with 80%, 83%, and 82% yield, respectively (Fig. 7B). These results indicated that this process is not a radical reaction, but a nucleophilic reaction.

**Figure 8:**
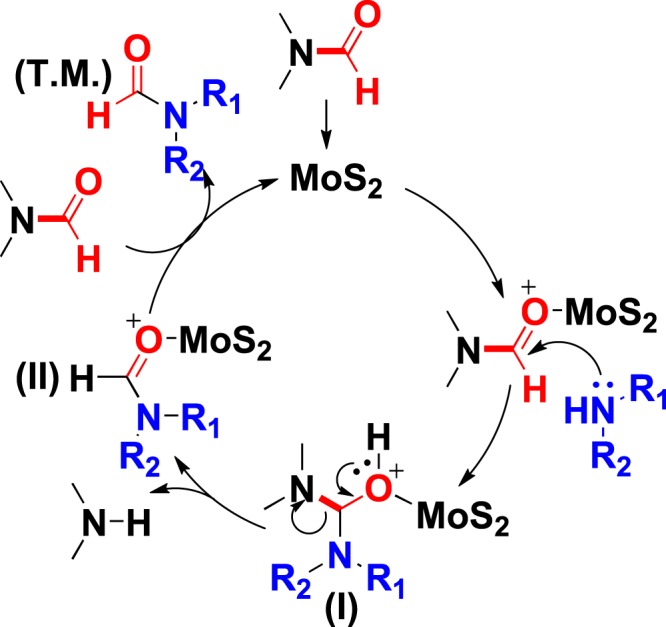
Proposed mechanism for transamidation using MoS_2_ as catalyst.

Based on the radical blocking experiments and previous reported metal-catalyzed transamidation reactions^13–15^, the reaction mechanism was proposed (Fig. 8). First, MoS_2_ coordinated with DMF, and the carbonyl group is activated. Then, substrate amine acting as a nucleophilic reagent attacks the carbonyl group of the activated DMF. Subsequently, a tetrahedral intermediate (**I**) is generated. And then, the sterically congested intermediate (**I**) is disintegrated with a proton transfer, and intermediate **II** is formed. Finally, a ligand exchange reaction occurs between intermediate **II** and DMF to release the target molecule (**T**.**M**.).

## Conclusions

In summary, an efficient MoS_2_-catalyzed transamidation reaction using amides as carbonyl sources was reported. The advantages of this reaction are the readily available and inexpensive metal applied as catalyst, cheap amides applied as carbonyl source, scalable, broad scopes, free of any additives such as acid, base, ligand, and etc.

## Materials and Methods

### General Information

The prepared thin-layer chromatography (Prep TLC) was performed for product purification using Sorbent Silica Gel 60 F254 TLC plates and visualized with ultraviolet light. IR spectra were recorded on a new Fourier transform infrared spectroscopy. ^1^H, ^13^C and ^19^F NMR spectra were recorded on 400, 100, 377 MHz NMR spectrometer using CDCl_3_ as solvent unless otherwise stated. HRMS were made by means of ESI. Melting points were measured on micro melting point apparatus and uncorrected. Unless otherwise noted, all reagents were weighed and handled in the air, and all reactions were carried out in a sealed tube under an atmosphere of argon. Unless otherwise noted, all reagents were purchased from reagent company, and used without further purifications. Notably, the powder MoS_2_ were used in this work.

### Experimental Section

A typical experimental procedure for transamidation was conducted as follows: A solution of amine (0.2 mmol), MoS_2_ (12.5 mol%, 4 mg) in DMF (1.0 mL) was stirred in a sealed tube under an atmosphere of argon at 150 °C for 18 h. After being cooled to room temperature, the reaction mixture was filtered, washed with ethyl acetate (20 mL). Afterward, the solution was added 10 mL water and extracted with ethyl acetate (3 × 15 mL), and then the combined organic layers were dried with Na_2_SO_4_. The solvent was evaporated under vacuum and the crude product was purified by Prep TLC on silica gel with petroleum ether and ethyl acetate to obtain the pure product.

## Supplementary information

Supplementary Information (traceless)
