# Supplementary material for: MoS2-Catalyzed transamidation reaction
Source: Sci Rep. 2019 Feb 22;9:2536. doi: 10.1038/s41598-019-39210-5 (PMC6385372; doi:10.1038/s41598-019-39210-5)
Supplement: Supplementary file 1 — Supplementary Information (traceless) [file 41598_2019_39210_MOESM1_ESM.pdf]

Supplementary Information

MoS_2-Catalyzed transamidation reaction

Feng Zhang,[1,*] Lesong Li,[2] Juan Ma,[2] Hang Gong*[,2]

[1]College of Science, Hunan Agricultural University, Changsha, 410128, (China)

[2]The Key Laboratory of Environmentally Friendly Chemistry and Application of the Ministry of Education, College of Chemistry, Xiangtan University, Xiangtan 411105, China.

E-mail: zhangf@iccas.ac.cn; E-mail: hgong@xtu.edu.cn)

Table of Contents

1. Characterization data for products..1

2. References...13

3. Copies of ^{1}H, ^{13}C and ^{19}F NMR spectra of products 14

1. Characterization data for products

2-Formyl-1,2,3,4-tetrahydroisoquinoline (2a).[1] Purified by Prep TLC (PET/EtOAc = 3/1); Yellow oil; Isolated yield 99%; Two rotamers (ratio 63:37) were observed. ^{1}H NMR (400 MHz, $CDCl_3$) δ 8.23 and 8.18 (s and s, total 1H), 7.21-7.10 (m, 4H), 4.67 and 4.52 (s and s, total 2H), 3.77 and 3.63 (t, *J* = 6.2 Hz and t, *J* = 5.8 Hz, total 2H), 2.90-2.84 (m, 2H); ^{13}C NMR (100 MHz, $CDCl_3$) δ 161.8 (major rotamer), 161.2 (minor rotamer), 134.4 (minor rotamer), 133.6 (major rotamer), 132.3 (minor rotamer), 131.8 (major rotamer), 129.2 (minor rotamer), 129.0 (major rotamer), 127.1, 126.7-126.5 (m), 125.9, 47.3 (minor rotamer), 43.3 (major rotamer), 42.3 (major rotamer), 38.0 (minor rotamer), 29.7 (major rotamer), 27.9 (minor rotamer); IR (neat) 3151, 2931, 2862, 1672, 1439, 1282, 1197, 1049, 930, 882, 751, 675 cm^{-1}; HRMS (ESI) m/z $[M+Na]^+$ calcd for $C_{10}H_{11}NNaO$ 184.0733, found 184.0733.

2-Formyl-l,2,3,4-tetrahydro-6-methoxyisoquinoline (2b).[2] Purified by Prep TLC (PET/EtOAc = 3/1); Yellow solid; Isolated yield 91%; mp 63-64 °C; Two rotamers (ratio 57:42) were observed. ^{1}H NMR (400 MHz, $CDCl_3$) δ 8.24 and 8.19 (s and s, total 1H), 7.06-7.01 (m, 1H), 6.80-6.76 (m, 1H), 6.67 (d, *J* = 10.0 Hz, 1H), 4.63 and 4.49 (s and s, total 2H), 3.79-3.75 (m, 3.81H), 3.63 (t, *J* = 5.8 Hz, 1.21H), 2.90-2.83 (m, 2H); ^{13}C NMR (100 MHz, CDCl3) δ 161.7 (major rotamer), 161.2 (minor rotamer), 158.5 (minor rotamer), 158.2 (major rotamer), 135.7 (minor rotamer), 134.8 (major rotamer), 127.7 (major rotamer), 126.9 (minor rotamer), 124.4 (minor rotamer), 123.9 (major rotamer), 113.8 (minor rotamer), 113.6 (major rotamer), 113.1 (major rotamer), 112.8 (minor rotamer), 55.3 (s), 46.9 (minor rotamer), 43.2 (major rotamer), 41.9 (major rotamer), 37.9 (minor rotamrer), 30.0 (major rotamer), 28.3 (minor rotamer); IR (neat) 3140, 1672, 1612, 1508, 1402, 1277, 1262, 1119, 529 cm^{-1}; HRMS (ESI) m/z $[M+HCO2H-H]^-$ calcd for $C_{12}H_{14}NO_4$ 236.0917, found 236.0921.

7-Bromo-3,4-dihydroisoquinoline-2(1H)-carbaldehyde (2c). Purified by Prep TLC (PET/EtOAc = 3/1); Yellow solid; Isolated yield 90%; mp 58-60 °C; Two rotamers (ratio 64:36) were observed. ^{1}H NMR (400 MHz, $CDCl_3$) δ 8.24 and 8.19 (s and s, total 1H), 7.33-7.26 (m, 2H), 7.02 (t, *J* = 8.8 Hz, 1H), 4.65 and 4.52 (s and s, total 2H), 3.78 and 3.64 (t, *J* = 6.2 Hz and t, *J* = 5.8 Hz, total 2H), 2.87-2.80 (m, 2H); ^{13}C NMR (100 MHz, $CDCl_3$) δ 161.7 (major rotamer), 161.2 (minor rotamer), 134.3 (minor rotamer), 133.9 (major rotamer), 133.4 (minor rotamer), 132.5 (major rotamer), 130.9 (minor rotamer), 130.6 (major rotamer), 130.2 (minor rotamer), 129.8 (major rotamer), 129.5 (major rotamer), 128.8 (minor rotamer), 120.3 (major rotamer), 120.0 (minor rotamer), 46.9 (minor rotamer), 43.0 (major rotamer), 41.9 (major rotamer), 37.8 (minor rotamer), 29.3

(major rotamer), 27.5 (minor rotamer); IR (neat) 3140, 1672, 1402, 1191, 1157, 1116, 1075, 1051, 932, 829 cm^{-1}; HRMS (ESI) m/z $[M+NH4]^+$ calcd for $C_{10}H_{14}BrN_2O$ 257.0284, found 257.0275.

7-Nitro-3,4-dihydroisoquinoline-2(1H)-carbaldehyde (2d). Purified by Prep TLC (PET/EtOAc = 1/1); brown oil; Isolated yield 75%; Two rotamers (ratio 61:39) were observed. ^{1}H NMR (400 MHz, $CDCl_3$) δ 8.30 and 8.24 (s and s, total 1H), 8.08-8.04 (m, 2H), 7.36-7.28 (m, 1H), 4.79 and 4.66 (s and s, total 2H), 3.85 and 3.72 (t, J = 6.0 Hz and t, J = 5.8 Hz, total 2H), 3.05-2.97 (m, 2H); ^{13}C NMR (100 MHz, $CDCl_3$) δ 161.6 (major rotamer), 161.1 (minor rotamer), 146.8 (major rotamer), 146.5 (minor rotamer), 142.2 (minor rotamer), 141.2 (major rotamer), 133.8 (minor rotamer), 133.5 (major rotamer), 130.4 (minor rotamer), 130.1 (major rotamer), 122.1 (minor rotamer), 121.9 (major rotamer), 121.7 (major rotamer), 121.3 (minor rotamer), 47.1 (minor rotamer), 42.5 (major rotamer), 42.1 (major rotamer), 37.3 (minor rotamer), 29.9 (major rotamer), 28.2 (minor rotamer); IR (neat) 3129, 1672, 1525, 1402, 1347, 1088, 855, 744, 531 cm^{-1}; HRMS (ESI) m/z $[M+CH_3CO_2H-H]^-$ calcd for $C_{12}H_{13}N_2O_5$ 265.0819, found 265.0824.

2-Formyl-1,3-dihydro-isoindole (2e).[3] Purified by Prep TLC (PET/EtOAc = 3/1); Black oil; Isolated yield 85%; ^{1}H NMR (400 MHz, $CDCl_3$) δ 8.43 (s, 1H), 7.32-7.27 (m, 4H), 4.90 (s, 2H), 4.77 (s, 2H); ^{13}C NMR (100 MHz, $CDCl_3$) δ 161.6, 136.0, 135.3, 128.1, 127.8, 123.3, 122.9, 51.5, 49.9; IR (neat) 3140, 1668, 1465, 1402, 1159, 1092, 747, 608 cm^{-1}; HRMS (ESI) m/z $[M+Cl]^-$ calcd for C_9H_9ClNO 182.0367, found 182.0368.

5-Formyl-4,5,6,7-tetrahydrothieno[3,2-c]pyridine (2f).[4] Purified by Prep TLC (PET/EtOAc = 3/1); Yellow oil; Isolated yield 95%; Two rotamers (ratio 62:38) were observed. ^{1}H NMR (400 MHz, $CDCl_3$) δ 8.25 and 8.21 (s and s, total 1H), 7.18-7.16 (m, 1H), 6.82-6.79 (m, 1H), 4.61 and 4.48 (s and s, total 2H), 3.86 and 3.70 (t, J = 5.6 Hz and t, J = 5.8 Hz, total 2H), 2.95-2.88 (m, 2H); ^{13}C NMR (100 MHz, $CDCl_3$) δ 161.8 (major rotamer), 161.4 (minor rotamer), 133.9 (minor rotamer), 132.2 (major rotamer), 130.8 (major rotamer), 130.8 (minor rotamer), 125.0 (major rotamer), 124.4 (minor rotamer), 123.9 (s), 45.8 (minor rotamer), 43.8 (major rotamer), 40.7 (major rotamer), 38.0 (minor rotamer), 25.9 (major rotamer), 24.5 (minor rotamer); IR (neat) 3129, 1705, 1670, 1433, 1402, 1314, 1176, 1043, 1018, 824, 706, 593 cm^{-1}; HRMS (ESI) m/z $[M+NH4]^+$ calcd for $C_8H_{13}N_2SO$ 185.0743, found 185.0735.

4-Phenylpiperazine-1-carboxaldehyde (2g).[5] Purified by Prep TLC (PET/EtOAc = 3/1); Yellow solid; Isolated yield 93%; mp 86-87 ^oC; ^{1}H NMR (400 MHz, $CDCl_3$) δ 8.08 (s, 1H), 7.30-7.26 (m, 2H), 6.94-6.90 (m, 3H), 3.69 (t, J = 5.2 Hz, 2H), 3.51 (t, J = 5.0 Hz, 2H), 3.18-3.11 (m, 4H); ^{13}C NMR (100 MHz, $CDCl_3$) δ 160.8, 151.0, 129.3, 120.9, 117.1, 50.5, 49.4, 45.6, 40.0; IR (neat) 3131,

1664, 1402, 1152, 1115, 529 cm^{-1}; HRMS (ESI) m/z $[M+K]^+$ calcd for $C_{11}H_{14}N_2KO$ 229.0738, found 229.0742.

4-Phenylpiperidine-1-carbaldehyde (2h).[5] Purified by Prep TLC (PET/EtOAc = 3/1); Yellow solid; Isolated yield 99%; mp 98-99 °C; ^1H NMR (400 MHz, $CDCl_3$) δ 8.06 (s, 1H), 7.33-7.18 (m, 5H), 4.56 (d, *J* = 13.6 Hz, 1H), 3.72 (d, *J* = 13.2 Hz, 1H), 3.19 (t, *J* = 13 Hz, 1H), 2.77-2.70 (m, 2H), 1.92 (t, *J* = 15.8 Hz, 2H), 1.64-1.57 (m, 2H); ^{13}C NMR (100 MHz, $CDCl_3$) δ 160.9, 144.9, 128.7, 126.7, 126.7, 46.5, 42.9, 40.3, 33.9, 32.4; IR (neat) 3140, 1675, 1653, 1402, 1170, 1064, 759, 699 cm^{-1}; HRMS (ESI) m/z $[M+Na]^+$ calcd for $C_{12}H_{15}NNaO$ 212.1046, found 212.1048.

***N*-Methyl-*N*-benzylformamide (2i).**[6] Purified by Prep TLC (PET/EtOAc = 3/1); Yellow oil; Isolated yield 72%; Two rotamers (ratio 57:43) were observed. ^1H NMR (400 MHz, $CDCl_3$) δ 8.30 and 8.17 (s and s, total 1H), 7.38-7.20 (m, 5H), 4.53 and 4.40 (s and s, total 2H), 2.86 and 2.79 (s and s, total 3H); ^{13}C NMR (100 MHz, $CDCl_3$) δ 162.9 (major rotamer), 162.7 (minor rotamer), 136.0 (minor rotamer), 135.7 (major rotamer), 128.9 (major rotamer), 128.7 (minor rotamer), 128.3 (major rotamer), 128.2 (minor rotamer), 127.7 (minor rotamer), 127.4 (major rotamer), 53.6 (major rotamer), 47.8 (minor rotamer), 34.1 (minor rotamer), 29.5 (major rotamer); IR (neat) 3122, 1664, 1402, 1379, 1140, 1066, 1081, 705, 529 cm^{-1}; HRMS (ESI) m/z $[M+CH_3CO_2H-H]^-$ calcd for $C_{11}H_{14}NO_3$ 208.0968, found 208.0977.

***N*-Benzyl-*N*-ethylformamide (2j).**[7] Purified by Prep TLC (PET/EtOAc = 3/1); Yellow oil; Isolated yield 56%; Two rotamers (ratio 50:50) were observed. ^1H NMR (400 MHz, $CDCl_3$) δ 8.26 and 8.23 (s and s, total 1H), 7.39-7.21 (m, 5H), 4.55 and 4.40 (s and s, total 2H), 3.29 and 3.20 (q, *J* = 7.2 Hz and q, *J* = 7.2 Hz, total 2H), 1.15 and 1.06 (t, *J* = 7.2 Hz and t, *J* = 7.2 Hz, total 3H); ^{13}C NMR (100 MHz, $CDCl_3$) δ 162.7, 136.5 (major rotamer), 136.2 (minor rotamer), 128.9 (minor rotamer), 128.7 (major rotamer), 128.2 (major rotamer), 128.1 (minor rotamer), 127.6 (minor rotamer), 127.5 (major rotamer), 50.9 (minor rotamer), 44.8 (major rotamer), 41.5 (major rotamer), 36.8 (minor rotamer), 14.4 (major rotamer), 12.2 (minor rotamer); IR (neat) 3140, 1672, 1497, 1402, 1109, 1079, 740, 703, 528 cm^{-1}; HRMS (ESI) m/z $[M+CH_3CO_2H-H]^-$ calcd for $C_{12}H_{16}NO_3$ 222.1125, found 222.1119.

***N*-(4-Methoxybenzyl)-*N*-methylformamide (2k).** Purified by Prep TLC (PET/EtOAc = 3/1); Yellow oil; Isolated yield 96%; Two rotamers (ratio 59:41) were observed. ^1H NMR (400 MHz, $CDCl_3$) δ 8.27 and 8.13 (s and s, total 1H), 7.20-7.12 (m, 2H), 6.91-6.85 (m, 2H), 4.46 and 4.33 (s and s, total 2H), 3.81 and 3.80 (s and s, total 3H), 2.82 and 2.75 (s and s, total 3H); ^{13}C NMR (100 MHz, $CDCl_3$) δ 162.6 (major rotamer), 162.5 (minor rotamer), 159.4 (major rotamer), 159.1 (minor rotamer), 130.5, 129.7 (minor rotamer), 128.8 (major rotamer), 128.1 (minor rotamer), 127.6 (major

rotamer), 114.2 (major rotamer), 114.0 (minor rotamer), 113.6, 55.3 (major rotamer, d, J = 4.4 Hz), 53.0 (minor rotamer), 47.1 (major rotamer), 44.9 (minor rotamer), 34.0 (minor rotamer), 29.2 (major rotamer); IR (neat) 3140, 1671, 1612, 1515, 1402, 1303, 1249, 1176, 1079, 1032, 846, 814, 559 cm^{-1}; HRMS (ESI) m/z $[M+NH4]^+$ calcd for $C_{10}H_{17}N_2O_2$ 197.1285, found 197.1304.

***N*-Methyl-*N*-[[4-(trifluoromethyl)phenyl]methyl]methanamide (2l).** Purified by Prep TLC (PET/EtOAc = 3/1); Yellow oil; Isolated yield 64%; Two rotamers (ratio 53:47) were observed. ^{1}H NMR (400 MHz, $CDCl_3$) δ 8.31 and 8.19 (s and s, total 1H), 7.66-7.59 (m, 2H), 7.39-7.34 (m, 2H), 4.59 and 4.48 (s and s, total 2H), 2.90 and 2.80 (s and s, total 3H); ^{13}C NMR (100 MHz, $CDCl_3$) δ 162.9 (minor rotamer), 162.8 (major rotamer), 140.1 (major rotamer), 139.9 (minor rotamer), 130.6 (major rotamer), 130.3 (minor rotamer), 130.1 (major rotamer), 129.8 (minor rotamer), 128.4 (major rotamer), 127.7 (minor rotamer), 126.0 (minor rotamer, q, J = 3.7 Hz), 125.7 (major rotamer q, J = 3.7 Hz), 125.4 (major rotamer), 125.3 (minor rotamer), 122.7 (major rotamer), 122.6 (minor rotamer), 53.0 (minor rotamer), 47.4 (major rotamer), 34.2 (major rotamer), 29.6 (minor rotamer) ;^{19}F NMR (377 MHz, CDCl3) δ -62.53 (major rotamer, s), -62.58 (minor rotamer, s); IR (neat) 3140, 2361, 2343, 1675, 1621, 1402, 1327, 1113, 1068, 1019, 848, 818 cm-^{1}; HRMS (ESI) m/z $[M+NH4]^+$ calcd for $C_{10}H_{14}F_3N_2O$ 235.1053, found 235.1067.

***N*-Methyl-*N*-phenethyl-formamide (2m).**[8] Purified by Prep TLC (PET/EtOAc = 3/1); Yellow oil; Isolated yield 63%; Two rotamers (ratio 63:37) were observed. ^{1}H NMR (400 MHz, $CDCl_3$) δ 8.01 and 7.80 (s and s, total 1H), 7.33-7.13 (m, 5H), 3.56 and 3.47 (t, J = 7.6 Hz and t, J = 7.0 Hz, total 2H), 2.90-2.82 (m, 5H); ^{13}C NMR (100 MHz, $CDCl_3$) δ 162.7 (major rotamer), 162.5 (minor rotamer), 138.6 (minor rotamer), 137.8 (major rotamer), 128.8 (major rotamer), 128.8 (minor rotamer), 128.7 (major rotamer), 128.6 (minor rotamer), 126.9 (major rotamer), 126.5 (minor rotamer), 51.3 (major rotamer), 46.0 (minor rotamer), 35.1 (minor rotamer), 34.8 (major rotamer), 33.2 (minor rotamer), 29.8 (minor rotamrer); IR (neat) 3140, 1666, 1402, 1152, 529 cm^{-1}; HRMS (ESI) m/z $[M+H]^+$ calcd for $C_{10}H_{14}NO$ 164.1070, found 164.1071.

***N*-Methyl-*N*-(naphthalen-1-ylmethyl)methanamide (2n).** Purified by Prep TLC (PET/EtOAc = 3/1); Yellow oil; Isolated yield 72%; Two rotamers (ratio 61:38) were observed. ^{1}H NMR (400 MHz, $CDCl_3$) δ 8.38 and 8.17 (s and s, total 1H), 8.10 and 7.91-7.82 (d, J = 8.0 Hz and m, total 3H), 7.55-7.26 (m, 4H), 4.98 and 4.87 (s and s, total 2H), 2.85 and 2.74 (s and s, total 3H); ^{13}C NMR (100 MHz, $CDCl_3$) δ 163.3 (minor rotamer), 162.4 (major rotamer), 133.9, 131.6, 131.3, 131.1, 131.0, 129.1, 128.9, 128.9, 128.7, 127.8, 126.8, 126.2, 125.6, 125.4, 125.2, 123.9 (major rotamer), 122.3 (minor rotamer), 51.1 (minor rotamer), 45.9 (major rotamer), 34.1 (major rotamer), 30.1 (minor

rotamer); IR (neat) 3140, 1672, 1510, 1402, 1258, 1161, 1081, 803, 779, 529 cm^{-1}; HRMS (ESI) m/z $[M+H]^+$ calcd for $C_{13}H_{14}NO$ 200.1070, found 200.1069.

***N*-Formylfluoxetine (2o).** Purified by Prep TLC (PET/EtOAc = 2/1); Yellow oil; Isolated yield 78%; Two rotamers (ratio 58:42) were observed. ^1H NMR (400 MHz, $CDCl_3$) δ 8.03 and 7.99 (s and s, total 1H), 7.43 (d, *J* = 8.8 Hz, 2H), 7.38-7.26 (m, 5H), 6.90-6.87 (m, 2H), 5.20 and 5.13 (dd, *J* = 8.8, 4.4 Hz and dd, *J* = 8.8, 4.0 Hz, total 1H), 3.58-3.53 and 3.42-3.36 (m and m, total 2H), 2.94 and 2.90 (s and s, total 3H), 2.25-2.18 and 2.15-2.09 (m and m, total 2H); ^{13}C NMR (100 MHz, $CDCl_3$) δ 162.8 (major rotamer), 162.7 (minor rotamer), 160.2 (minor rotamer), 159.9 (major rotamer), 140.4 (minor rotamer), 139.9 (major rotamer), 129.1 (major rotamer), 128.9 (minor rotamer), 128.3 (major rotamer), 128.1 (minor rotamer), 127.0-126.8 (m), 125.7 (minor rotamer), 125.6 (major rotamer), 123.1 (s), 115.7 (minor rotamer), 115.7 (major rotamer), 78.2 (minor rotamer), 76.9 (major rotamer), 46.0 (major rotamer), 41.6 (minor rotamer), 36.9 (major rotamer), 35.9 (minor rotamer), 34.9 (minor rotamer), 29.6 (major rotamer); ^{19}F NMR (377 MHz, CDCl3) δ -61.52 (minor rotamer, s); -61.59 (major rotamer, s); IR (neat) 3140, 1675, 1616, 1519, 1329, 1251, 1161, 1113, 1068, 837, 703, 527 cm^{-1}; HRMS (ESI) m/z $[M+Na]^+$ calcd for $C_{18}H_{18}F_3NNaO_2$ 360.1182, found 360.1178.

***N*-Benzylformamide (2p).**[9] Purified by Prep TLC (PET/EtOAc = 3/1); Yellow solid; Isolated yield 91%; mp 54-58 °C; Two rotamers (ratio 84:16) were observed. ^1H NMR (400 MHz, $CDCl_3$) δ 8.25 and 8.17 (s and d, *J* = 12 Hz, total 1H), 7.35-7.24 (m, 5H), 6.00 (br s, 1H), 4.48 and 4.41 (d, *J* = 6.0 Hz and d, *J* = 6.4 Hz, total 2H); ^{13}C NMR (100 MHz, $CDCl_3$) δ 164.8 (minor rotamer), 161.1 (major rotamer), 137.6 (major rotamer), 137.5 (minor rotamer), 129.0 (minor rotamer), 128.8 (major rotamer), 128.0 (minor rotamer), 127.8 (major rotamer), 127.7 (major rotamer), 127.0 (minor rotamer), 45.7 (minor rotamer), 42.2 (major rotamer); IR (neat) 3140, 1666, 1402, 699, 526 cm^{-1}; HRMS (ESI) m/z $[M+H]^+$ calcd for $C_8H_{10}NO$ 136.0757, found 136.0747.

***N*-(2-Phenylethyl)formamide (2q).**[6] Purified by Prep TLC (PET/EtOAc = 2/1); Yellow oil; Isolated yield 98%; Two rotamers (ratio 83:17) were observed. ^1H NMR (400 MHz, $CDCl_3$) δ 8.10 and 7.88 (s and d, *J* = 12 Hz, total 1H), 7.32-7.20 (m, 5H), 5.82 (br s, 1H), 3.59-3.44 (m, 2H), 2.86-2.80 (m, 2H); ^{13}C NMR (100 MHz, $CDCl_3$) δ 164.6 (minor rotamer), 161.3 (major rotamer), 138.5 (major rotamer), 137.6 (minor rotamer), 128.9-128.7 (m), 126.9 (minor rotamer), 126.7 (major rotamer), 43.2 (minor rotamer), 39.2 (major rotamer), 37.7 (minor rotamer), 35.5 (major rotamer); IR (neat) 3140, 1670, 1402, 1154, 689, 527 cm^{-1}; HRMS (ESI) m/z $[M+Na]^+$ calcd for $C_9H_{11}NNaO$ 172.0733, found 172.0741.

***N*-Formyl-3-phenylpropylamine (2r).**[10] Purified by Prep TLC (PET/EtOAc = 2/1); Yellow oil;

Isolated yield 99%; Two rotamers (ratio 81:19) were observed. ^{1}H NMR (400 MHz, $CDCl_3$) δ 8.13 and 8.00 (s and d, *J* = 12 Hz, total 1H), 7.32-7.16 (m, 5H), 6.03 (br s, 1H), 3.31 and 3.20 (q, *J* = 6.8 Hz and q, *J* = 6.8 Hz, total 2H), 2.68-2.63 (m, 2H), 1.88-1.81 (m, 2H); ^{13}C NMR (100 MHz, $CDCl_3$) δ 164.9 (minor rotamer), 161.5 (major rotamer), 141.2 (major rotamer), 140.6 (minor rotamer), 128.7 (minor rotamer), 128.5 (major rotamer), 128.4 (major rotamer), 126.3 (minor rotamer), 126.1 (major rotamer), 41.1 (minor rotamer), 37.8 (major rotamer), 33.2 (major rotamer), 32.6 (minor rotamer), 32.5 (minor rotamer), 31.1 (major rotamer); IR (neat) 3122, 1666, 1402, 1154, 1113, 749, 701, 529 cm^{-1}; HRMS (ESI) m/z $[M+Na]^+$ calcd for $C_{10}H_{13}NNaO$ 186.0889, found 186.0897.

***N*-(1-Phenylethyl)formamide (2s).**[11] Purified by Prep TLC (PET/EtOAc = 3/1); Yellow oil; Isolated yield 62%; Two rotamers (ratio 80:20) were observed. ^{1}H NMR (400 MHz, $CDCl_3$) δ 8.11 (s, 1H), 7.36-7.26(m, 5H), 6.34 (br s, 1H), 5.21-5.14 and 4.70-4.65 (m and m, total 1H), 1.55 and 1.49 (d, *J* = 6.8 Hz and d, *J* = 6.8 Hz, total 3H); ^{13}C NMR (100 MHz, $CDCl_3$) δ 160.5, 142.6, 129.0 (minor rotamer), 128.8 (major rotamer), 127.8 (minor rotamer), 127.5 (major rotamer), 126.2 (major rotamer), 125.8 (minor rotamer), 51.8 (minor rotamer), 47.6 (major rotamer), 23.6 (minor rotamer), 21.8 (major rotamer); IR (neat) 3100, 1662, 1534, 1497, 1402, 1238, 1118, 762, 698, 609 cm^{-1}; HRMS (ESI) m/z $[M+H]^+$ calcd for $C_9H_{12}NO$ 150.0913, found 150.0912.

***N*-Formyl-1-indanamin (2t).**[12] Purified by Prep TLC (PET/EtOAc = 3/1); Yellow solid; Isolated yield 69%; mp 109-110 ^{o}C; Two rotamers (ratio 89:11) were observed. ^{1}H NMR (400 MHz, $CDCl_3$) δ 8.24 and 8.20 (s and s, total 1H), 7.29-7.20 (m, 4H), 5.57 (br s, 1H), 5.54 and 4.99 (q, *J* = 8.0 Hz and q, *J* = 8.0 Hz, total 1H), 3.02-2.95 (m, 1H), 2.91-2.83 (m, 1H), 2.64-2.56 (m, 1H), 1.90-1.78 (m, 1H); ^{13}C NMR (100 MHz, $CDCl_3$) δ 163.9 (minor rotamer), 161.0 (major rotamer), 143.5 (major rotamer), 142.6 (minor rotamer), 128.5 (minor rotamer), 128.2 (major rotamer), 127.1 (minor rotamer), 126.9 (major rotamer), 125.1 (minor rotamer), 124.9 (major rotamer), 124.0 (major rotamer), 123.8 (minor rotamer), 57.5 (minor rotamer), 53.3 (major rotamer), 35.2 (minor rotamer), 34.0 (major rotamer), 30.3 (major rotamer), 29.9 (minor rotamer); IR (neat) 3118, 1640, 1547, 1402, 1154, 1115, 751, 529 cm^{-1}; HRMS (ESI) m/z $[M+CH_3CO_2H-H]^-$ calcd for $C_{12}H_{14}NO_3$ 220.0968, found 220.0971.

***N*-(2-Hydroxy-2-phenylethyl)formamide (2u).**[13] Purified by Prep TLC (PET/EtOAc = 1/1); Yellow oil; Isolated yield 66%; Two rotamers (ratio 82:18) were observed. ^{1}H NMR (400 MHz, $CDCl_3$) δ 8.09 and 7.87 (s and d, *J* = 12 Hz, total 1H), 7.35-7.26 (m, 5H), 6.35 (br s, 1H), 4.80 and 4.71 (dd, *J* = 8.4, 3.6 Hz and dd, *J* = 7.6, 4.0 Hz, total 1H), 3.73-3.67 and 3.40-3.38 (m and m, total 2H), 3.34-3.27 (m, 1H); ^{13}C NMR (100 MHz, $CDCl_3$) δ 165.5 (minor rotamer), 162.3 (major rotamer),

141.4 (major rotamer), 140.9 (minor rotamer), 128.8 (minor rotamer), 128.6 (major rotamer), 128.3 (minor rotamer), 128.0 (major rotamer), 125.9 (minor rotamer), 125.9 (major rotamer), 73.4 (minor rotamer), 73.0 (major rotamer), 49.3 (minor rotamer), 45.8 (major rotamer); IR (neat) 3140, 1670, 1523, 1495, 1402, 1239, 1198, 1096, 915, 755, 703, 533 cm^{-1}; HRMS (ESI) m/z $[M+H]^+$ calcd for $C_9H_{12}NO_2$ 166.0863, found 166.0860.

***N*-(Dodecyl)formamide (2v).**[6] Purified by Prep TLC (PET/EtOAc = 10/1); Gray solid; Isolated yield 97%; mp 33.2-34.5 °C; Two rotamers (ratio 80:20) were observed. ^1H NMR (400 MHz, $CDCl_3$) δ 8.15 and 8.03 (s and d, *J* = 12 Hz, total 1H), 6.05 (br s, 1H), 3.28 and 3.20 (q, *J* = 6.8 Hz and q, *J* = 6.8 Hz, total 2H), 1.54-1.49 (m, 2H), 1.30-1.26 (m, 18H), 0.88 (t, *J* = 6.8 Hz, 3H); ^{13}C NMR (100 MHz, $CDCl_3$) δ 164.7 (minor rotamer), 161.3 (major rotamer), 41.8 (minor rotamer), 38.2 (major rotamer), 31.9 (major rotamer), 31.2 (minor rotamer), 29.6-29.2 (m), 26.9 (major rotamer), 26.4 (minor rotamer), 22.7, 14.1; IR (neat) 3122, 1670, 1401, 1150, 1113, 529 cm^{-1}; HRMS (ESI) m/z $[M+K]^+$ calcd for $C_{13}H_{27}NKO$ 252.1724, found 252.1724.

***N*-Formylhexamethyleneimine (2w).**[9] Purified by Prep TLC (PET/EtOAc = 10/1); Yellow oil; Isolated yield 67%; Two rotamers (ratio 50:50) were observed. ^1H NMR (400 MHz, $CDCl_3$) δ 8.08 (s, 1H), 3.46 and 3.38 (t, *J* = 6.0 Hz and t, *J* = 6.0 Hz, total 4H), 1.76-1.70 (m, 4H), 1.62-1.55 (m, 4H); ^{13}C NMR (100 MHz, $CDCl_3$) δ 162.9, 47.7, 43.4, 30.3, 28.0, 27.0, 26.8; IR (neat) 3137, 2932, 2857, 1681, 1402, 1299, 1260, 1202, 1157, 885, 751, 654, 531 cm^{-1}; HRMS (ESI) m/z $[M+HCO_2H-H]^-$ calcd for $C_8H_{14}NO_3$ 172.0968, found 172.0978.

***N*-[2-(3,4-Dimethoxyphenyl)ethyl]formamide (2x).**[14] Purified by Prep TLC (PET/EtOAc = 1/1); Yellow oil; Isolated yield 84%; Two rotamers (ratio 85:15) were observed. ^1H NMR (400 MHz, $CDCl_3$) δ 8.12 and 7.90 (s and d, *J* = 12 Hz, total 1H), 6.83-6.68 (m, 3H), 5.96 (br s, 1H), 3.87 and 3.86 (s and s, total 6H), 3.54 and 3.45 (q, *J* = 6.4 Hz and q, *J* = 6.4 Hz, total 2H), 2.85-2.74 (m, 2H); ^{13}C NMR (100 MHz, $CDCl_3$) δ 164.6 (minor rotamer), 161.3 (major rotamer), 149.1 (minor rotamer), 149.0 (major rotamer), 147.9 (minor rotamer), 147.7 (major rotamer), 131.0 (major rotamer), 130.1 (minor rotamer), 120.9 (minor rotamer), 120.7 (major rotamer), 111.9 (minor rotamer), 111.8 (major rotamer), 111.4 (minor rotamer), 111.3 (major rotamer), 55.9(major rotamer), 55.9(minor rotamer), 43.3 (minor rotamer), 39.3 (major rotamer), 37.3 (minor rotamer), 35.1 (major rotamer); IR (neat) 3140, 3006, 2941, 2838, 1668, 1593, 1519, 1400, 1265, 1159, 1029, 811, 766 cm^{-1}; HRMS (ESI) m/z $[M+H]^+$ calcd for $C_{11}H_{16}NO_3$ 210.1125, found 210.1122.

***N*-Acetyl-1,2,3,4-tetrahydroisoquinoline (2y).**[15] Purified by Prep TLC (PET/EtOAc = 3/1); Yellow oil; Isolated yield 95%; Two rotamers (ratio 58:42) were observed. ^1H NMR (400 MHz, $CDCl_3$) δ

7.20-7.13 (m, 4H), 4.72 and 4.61 (s and s, total 2H), 3.81 and 3.67 (t, *J* = 6.0 Hz and t, *J* = 6.0 Hz, total 2H), 2.90 and 2.86 (t, *J* = 6.0 Hz and t, *J* = 6.8 Hz, total 2H), 2.18 and 2.17 (s and s, total 3H); ^{13}C NMR (100 MHz, $CDCl_3$) δ 169.6 (major rotamer), 169.6 (minor rotamer), 135.1 (minor rotamer), 134.0 (major rotamer), 133.5 (major rotamer), 132.5 (minor rotamer), 129.0 (minor rotamer), 128.3 (major rotamer), 127.0 (minor rotamer), 126.7 (major rotamer), 126.6 (major rotamer), 126.6 (minor rotamer), 126.4 (major rotamer), 126.1 (minor rotamer), 48.1 (minor rotamer), 44.1 (major rotamer), 44.0 (major rotamer), 39.5 (minor rotamer), 29.5 (major rotamer), 28.5 (minor rotamer), 22.0 (minor rotamer), 21.7 (major rotamer); IR (neat) 3159, 2933, 2857, 1661, 1634, 1456, 1403, 1232, 1034, 930, 766, 636 cm^{-1}; HRMS (ESI) m/z $[M+HCO_2H-H]^-$ calcd for $C_{12}H_{14}NO_3$ 220.0968, found 220.0981.

***N*-Propionyl-1,2,3,4-tetrahydroisoquinoline (2z).**[16] Purified by Prep TLC (PET/EtOAc = 2/1); Yellow oil; Isolated yield 94%; Two rotamers (ratio 56:44) were observed. ^{1}H NMR (400 MHz, $CDCl_3$) δ 7.27-7.10 (m, 4H), 4.73 and 4.61 (s and s, total 2H), 3.83 and 3.67 (t, *J* = 6.0 Hz and t, *J* = 6.0 Hz, total 2H), 2.90 and 2.84 (t, *J* = 5.8 Hz and t, *J* = 6.0 Hz, total 2H), 2.46-2.42 (m, 2H), 1.21-1.16 (m, 3H); ^{13}C NMR (100 MHz, $CDCl_3$) δ 172.9 (major rotamer), 172.8 (minor rotamer), 135.2 (minor rotamer), 134.1 (major rotamer), 133.7 (major rotamer), 132.7 (minor rotamer), 129.0 (minor rotamer), 128.3 (major rotamer), 126.9, 126.7, 126.6, 126.5, 126.3, 126.0, 47.3 (minor rotamer), 44.3 (major rotamer), 43.1 (major rotamer), 39.7 (minor rotamer), 29.5 (major rotamer), 28.5 (minor rotamer), 27.0 (minor rotamer), 26.8 (major rotamer), 9.4 (major rotamer), 9.4 (minor rotamer); IR (neat) 3120, 2983, 2941, 2607, 2361, 1942, 1694, 1646, 1402, 1223, 1148, 1083, 746, 650 cm^{-1}; HRMS (ESI) m/z $[M+HCO_2H-H]^-$ calcd for $C_{13}H_{16}NO_3$ 234.1125, found 234.1125.

(3,4-Dihydroisoquinolin-2(1*H*)-yl)(thiophen-2-yl)methanone (2za). Purified by Prep TLC (n-hexane/DCM = 8/1); Yellow oil; Isolated yield 40%; ^{1}H NMR (400 MHz, $CDCl_3$) δ 7.46 (d, *J* = 4.8 Hz ,1H), 7.38 (d, *J* = 3.6 Hz ,1H), 7.20-7.06 (m, 5H), 4.87 (s, 2H), 3.93 (t, *J* = 5.8 Hz, 2H), 2.96 (t, *J* = 5.8 Hz, 2H); ^{13}C NMR (100 MHz, $CDCl_3$) δ 163.9, 137.6, 134.5, 133.0, 128.9, 128.9, 128.7, 126.8, 126.8, 126.6, 126.4, 44.9 (br s), 29.7, 29.2; IR (neat) 3152, 2361, 1620, 1523, 1402, 1297, 1251, 1101, 932, 818, 740, 721 cm^{-1}; HRMS (ESI) m/z $[M-H]^-$ calcd for $C_{14}H_{12}NSO$ 242.0645, found 242.0640.

(3,4-Dihydroisoquinolin-2(1*H*)-yl)(phenyl)methanone (2zb).[17] Purified by Prep TLC (*n*-hexane/DCM = 8/1); Yellow oil; Isolated yield 34%; Two rotamers (ratio 56:44) were observed. ^{1}H NMR (400 MHz, $CDCl_3$) δ 7.44 (s, 5H), 7.21-7.16(m, 4H), 4.90 and 4.59 (s and s, total 2H), 4.00 and 3.64 (s and s, total 2H), 2.97 and 2.88 (s and s, total 2H); ^{13}C NMR (100 MHz, $CDCl_3$) δ 171.0

136.1, 133.0, 129.8, 128.6, 127.3-126.4(m), 49.8, 45.3, 44.9, 40.5, 29.7, 28.3; IR (neat) 3153, 2363, 2345, 1633, 1402, 1299, 1258, 1107, 934, 753, 699, 527 cm^{-1}; HRMS (ESI) m/z $[M+H]^+$ calcd for $C_{16}H_{16}NO$ 238.1226, found 238.1222.

***N*-(4-phenylpiperazin-1-yl)acetamide (2zc).**[18] Purified by Prep TLC (PET/EtOAc = 2/1); Yellow oil; Isolated yield 89%; ^1H NMR (400 MHz, $CDCl_3$) δ 7.32-7.27 (m, 2H), 6.95-6.90 (m, 3H), 3.78 (t, *J* = 5.2 Hz, 2H), 3.63 (t, *J* = 5.2 Hz, 2H), 3.20-3.14 (m, 4H), 2.15 (s, 3H); ^{13}C NMR (100 MHz, $CDCl_3$) δ 169.1, 151.0, 129.3, 120.6, 116.7, 49.7, 49.4, 46.3, 41.4, 21.4; IR (neat) 3152, 2820, 1644, 1597, 1504, 1433, 1230, 1157, 997, 906, 760, 695 cm^{-1}; HRMS (ESI) m/z $[M+Na]^+$ calcd for $C_{12}H_{16}N_2NaO$ 227.1155, found 227.1157.

***N*-Methyl-*N*-phenethylacetamide (2zd).** Purified by Prep TLC (PET/EtOAc = 2/1); Pale yellow oil; Isolated yield 91%; Two rotamers (ratio 54:46) were observed. ^1H NMR (400 MHz, $CDCl_3$) δ 7.33-7.15 (m, 5H), 3.58 and 3.50 (t, *J* = 7.6 Hz and t, *J* = 7.2 Hz, total 2H), 2.94 and 2.87 (s and s, total 3H), 2.84 (t, *J* = 7.4 Hz, 2H), 2.06 and 1.85 (s and s, total 3H); ^{13}C NMR (100 MHz, $CDCl_3$) δ 170.7, 170.5, 139.2 (minor rotamer), 138.2 (major rotamer), 128.8 (m), 128.5, 126.8, 126.3, 52.6 (major rotamer), 49.8 (minor rotamer), 36.9 (minor rotamer), 34.8 (major rotamer), 33.8 (major rotamer), 33.4 (minor rotamrer), 22.0 (minor rotamer), 21.0 (major rotamer); IR (neat) 3029, 2935, 1651, 1402, 1305, 1202, 1129, 1079, 1033, 1005, 753, 703 cm^{-1}; HRMS (ESI) m/z $[M+Na]^+$ calcd for $C_{11}H_{15}NNaO$ 200.1046, found 200.1039.

***N*-Methyl-*N*-(naphthalen-1-ylmethyl)acetamide (2ze).**[19] Purified by Prep TLC (PET/EtOAc = 2/1); Yellow oil; Isolated yield 93%; Two rotamers (ratio 61:39) were observed. ^1H NMR (400 MHz, $CDCl_3$) δ 8.10 and 7.92-7.79 (d, *J* = 8.0 Hz and m, total 3H), 7.59-7.21 (m, 4H), 5.06 and 4.99 (s and s, total 2H), 3.06 and 2.83 (s and s, total 3H), 2.18 and 2.12 (s and s, total 3H); ^{13}C NMR (100 MHz, $CDCl_3$) δ 171.7 (minor rotamer), 170.6 (major rotamer), 133.9 (major rotamer), 133.8 (minor rotamer), 132.7, 131.7, 131.4, 130.7, 129.1, 128.6, 128.5, 128.1, 127.1, 126.6, 126.5, 126.1, 126.0, 125.6, 125.2, 124.0, 122.5 (minor rotamer), 122.0 (major rotamer), 52.1 (minor rotamer), 48.4 (major rotamer), 34.9 (major rotamer), 34.4 (minor rotamer), 22.1 (major rotamer), 21.3 (minor rotamer); IR (neat) 3140, 1655, 1402, 1262, 1163, 792, 529 cm^{-1}; HRMS (ESI) m/z $[M+Na]^+$ calcd for $C_{14}H_{15}NNaO$ 236.1046, found 236.1041.

5-Acetyl-4,5,6,7-tetrahydrothieno[3,2-c]pyridine (2zf).[20] Purified by Prep TLC (PET/EtOAc = 3/1); A pale yellow oil; Isolated yield 93%; Two rotamers (ratio 52:48) were observed. ^1H NMR (400 MHz, $CDCl_3$) δ 7.17-7.13 (m, 1H), 6.81-6.79 (m, 1H), 4.67 and 4.55 (s and s, total 2H), 3.91 and 3.74 (t, *J* = 5.8 Hz and t, *J* = 5.6 Hz, total 2H), 2.92 and 2.86 (t, *J* = 5.6 Hz and t, *J* = 5.6 Hz,

total 2H), 2.20 and 2.17 (s and s, total 3H); ^{13}C NMR (100 MHz, $CDCl_3$) δ 169.7 (minor rotamer), 169.5 (major rotamer), 134.4 (minor rotamer), 132.5 (major rotamer), 132.2 (minor rotamer), 131.1 (major rotamer), 125.2 (major rotamer), 124.5 (minor rotamer), 123.7 (minor rotamer), 123.5 (major rotamer), 46.4 (minor rotamer), 44.3 (major rotamer), 42.5 (major rotamer), 39.5 (minor rotamer), 25.6 (major rotamer), 24.7 (minor rotamer), 22.0 (minor rotamer), 21.7 (major rotamer); IR (neat) 3111, 3014, 2928, 2850, 1634, 1403, 1241, 1217, 1010, 893, 833, 708, 593 cm^{-1}; HRMS (ESI) m/z $[M+Na]^+$ calcd for $C_9H_{11}NSNaO$ 204.0454, found 204.0452.

***N*-Benzylacetamide (2zg).**[21] Purified by Prep TLC (PET/EtOAc = 2/1); A colorless solid; Isolated yield 92%; mp 64.3-66.2 °C; ^1H NMR (400 MHz, $CDCl_3$) δ 7.35-7.26 (m, 5H), 6.06 (br s, 1H), 4.40 (d, *J* = 5.6 Hz, 2H), 2.00 (s, 3H); ^{13}C NMR (100 MHz, $CDCl_3$) δ 170.1, 138.3, 128.7, 127.9, 127.5, 43.7, 23.3; IR (neat) 3282, 1651, 1556, 1402, 1290, 1079, 1031, 734, 699, 611 cm^{-1}; HRMS (ESI) m/z $[M+Na]^+$ calcd for $C_9H_{11}NNaO$ 172.0733, found 172.0728.

1-(4-phenylpiperazin-1-yl)propan-1-one (2zh). Purified by Prep TLC (PET/EtOAc = 2/1); Yellow oil; Isolated yield 85%; ^1H NMR (400 MHz, $CDCl_3$) δ 7.31-7.27 (m, 2H), 6.95-6.89 (m, 3H), 3.79 (t, *J* = 4.8 Hz, 2H), 3.62 (t, *J* = 4.6 Hz, 2H), 3.18-3.14 (m, 4H), 2.40 (q, *J* = 7.6 Hz, 2H), 1.18 (t, *J* = 7.6 Hz, 3H); ^{13}C NMR (100 MHz, $CDCl_3$) δ 172.4, 151.0, 129.3, 120.5, 116.7, 49.8, 49.4, 45.4, 41.5, 26.5, 9.5; IR (neat) 3137, 1653, 1601, 1498, 1402, 1230, 1157, 1029, 936, 760, 695 cm^{-1}; HRMS (ESI) m/z $[M+Na]^+$ calcd for $C_{13}H_{18}N_2NaO$ 241.1311, found 241.1305.

***N*-Methyl-*N*-phenethylpropionamide (2zi).** Purified by Prep TLC (PET/EtOAc = 2/1); Yellow oil; Isolated yield 88%; Two rotamers (ratio 53:47) were observed. ^1H NMR (400 MHz, $CDCl_3$) δ 7.33-7.15 (m, 5H), 3.58 and 3.50 (t, *J* = 7.8 Hz and t, *J* = 7.4 Hz, total 2H), 2.96 and 2.87 (s and s, total 3H), 2.84 (t, *J* = 7.4 Hz, 2H), 2.30 and 2.12 (q, *J* = 7.2 Hz and q, *J* = 7.6 Hz, total 2H), 1.14 and 1.03 (t, *J* = 7.4 Hz and t, *J* = 7.4 Hz, total 3H); ^{13}C NMR (100 MHz, $CDCl_3$) δ 173.8 (minor rotamer), 173.6 (major rotamer), 139.3 (major rotamer), 138.3 (minor rotamer), 128.9 (minor rotamer), 128.8 (major rotamer), 128.5, 126.8 (minor rotamer), 126.3 (major rotamer), 51.6 (minor rotamer), 50.1 (major rotamer), 36.0 (minor rotamer), 34.9 (major rotamer), 33.9 (major rotamer), 33.6 (minor rotamer), 26.9 (major rotamer), 25.9 (minor rotamer), 9.5 (minor rotamer), 9.3 (major rotamer); IR (neat) 3152, 1649, 1402, 1152, 531 cm^{-1}; HRMS (ESI) m/z $[M+H]^+$ calcd for $C_{12}H_{18}NO$ 192.1383, found 192.1379.

***N*-Methyl-*N*-(naphthalen-1-ylmethyl)propionamide (2zj).** Purified by Prep TLC (PET/EtOAc = 2/1); Yellow oil; Isolated yield 88%; Two rotamers (ratio 63:37) were observed. ^1H NMR (400 MHz, $CDCl_3$) δ 8.09 and 7.92-7.79 (d, *J* = 8.0 Hz and m, 3H), 7.58-7.40 (m, 3H), 7.33 and 7.20 (d, *J* = 6.8

Hz, and d, *J* = 7.2 Hz, 1H), 5.08 and 5.00 (s and s, total 2H), 3.08 and 2.83 (s and s, total 3H), 2.44-2.33 (m, 2H), 1.22 and 1.15 (t, *J* = 7.4 Hz and t, *J* = 7.6 Hz, total 3H); ^{13}C NMR (100 MHz, $CDCl_3$) δ 174.8 (minor rotamer), 173.7 (major rotamer), 133.9 (major rotamer), 133.8 (minor rotamer), 132.9 (major rotamer), 131.8 (major rotamer), 131.7 (minor rotamer), 130.7 (minor rotamer), 129.1 (minor rotamer), 128.6 (major rotamer), 128.4 (major rotamer), 128.0 (minor rotamer), 127.0 (minor rotamer), 126.5 (major rotamer), 126.1 (minor rotamer), 126.0 (major rotamer), 125.6 (minor rotamer), 125.2 (major rotamer), 124.0, 122.4 (minor rotamer), 122.0 (major rotamer), 51.1 (minor rotamer), 48.6 (major rotamer), 34.6 (minor rotamer), 34.0 (major rotamer), 27.0 (major rotamer), 26.1 (minor rotamer), 9.6 (minor rotamer), 9.5 (major rotamer); IR (neat) 3144, 1651, 1510, 1403, 1256, 1120, 1066, 794 cm^{-1}; HRMS (ESI) m/z $[M+Na]^+$ calcd for $C_{15}H_{17}NNaO$ 250.1202, found 250.1196.

1-(6,7-dihydrothieno[3,2-c]pyridin-5(4*H*)-yl)propan-1-one (2zk). Purified by Prep TLC (PET/EtOAc = 2/1); Pale yellow oil; Isolated yield 88%; Two rotamers (ratio 53:47) were observed. ^{1}H NMR (400 MHz, $CDCl_3$) δ 7.16-7.12 (m, 1H), 6.82-6.78 (m, 1H), 4.68 and 4.55 (s and s, total 2H), 3.93 and 3.75 (t, *J* = 5.8 Hz and t, *J* = 5.6 Hz, total 2H), 2.91 and 2.86 (t, *J* = 5.6 Hz and t, *J* = 5.6 Hz, total 2H), 2.48-2.39 (m, 2H), 1.22-1.16 (m, 3H); ^{13}C NMR (100 MHz, $CDCl_3$) δ 172.9, 172.8, 134.5, 132.7, 132.2, 131.2, 125.3, 124.5, 123.6, 123.4, 45.5 (minor rotamer), 43.3 (major rotamer), 42.6 (major rotamer), 39.7 (minor rotamer), 27.1 (minor rotamer), 26.8 (major rotamer), 25.7 (major rotamer), 24.8 (minor rotamer), 9.6 (major rotamer), 9.4 (minor rotamer); IR (neat) 3122, 1648, 1429, 1402, 1264, 1224, 1046, 889, 831, 706, 658 cm^{-1}; HRMS (ESI) m/z $[M+Na]^+$ calcd for $C_{10}H_{13}NSNaO$ 218.0610, found 218.0603.

***N*-Benzylpropionamide (2zl).**[22] Purified by Prep TLC (PET/EtOAc = 2/1); Yellow oil; Isolated yield 78%; ^{1}H NMR (400 MHz, $CDCl_3$) δ 7.34-7.25 (m, 5H), 5.96 (br s, 1H), 4.42 (d, *J* = 5.2 Hz, 2H), 2.23 (q, *J* = 7.6 Hz, 2H), 1.17 (t, *J* = 7.6 Hz, 3H); ^{13}C NMR (100 MHz, $CDCl_3$) δ 173.7, 138.4, 128.7, 127.8, 127.5, 43.6, 29.7, 9.9; IR (neat) 3289, 1651, 1554, 1402, 1236, 1105, 1031, 732, 699, 501 cm^{-1}; HRMS (ESI) m/z $[M+Na]^+$ calcd for $C_{10}H_{13}NNaO$ 186.0889, found 186.0890.

2. References

1. Lv, H.; Xing, Q.; Yue, C.; Lei, Z.; Li, F. *Chem. Commun.* **2016**, *52*, 6545-6548.

2. Shinohara, T.; Takeda, A.; Toda, J.; Ueda, Y.; Kohno, M.; Sano, T. *Chem. Pharm. Bull.* **1998**, *46*, 918-927.

3. Lewin, A. H.; Frucht, M. *Org. Magn. Resonance.* **1975**, *7,* 206-225.

4. Sonawane, R. B.; Rasal, N. K.; Jagtap, S. V. *Org. Lett*. **2017**, *19*, 2078-2081.

5. Nguyen, T. V. Q.; Yoo, W.-J.; Kobayashi, S. *Angew. Chem. Int. Ed.* **2015**, *54*, 9209-9212.

6. Zhang, L.; Han, Z.; Zhao, X.; Wang, Z.; Ding, K. *Angew. Chem. Int. Ed*. **2015**, *54*, 6186-6189.

7. Ke, Z.; Zhang, Y.; Cui, X.; Shi, F. *Green Chem.* **2016**, *18*, 808-816.

8. Katritzky, A. R.; Yao, G.; Lan, X.; Zhao, X. *J. Org. Chem*. **1993**, *58*, 2086-2093.

9. Ortega, N.; Richter, C.; Glorius, F. *Org. Lett*. **2013**, *15*, 1776-1779.

10. Kang, B.; Hong, S. H. *Adv. Synth. Catal*. **2015**, *357*, 834-840.

11. Nakamura, T.; Tateishi, K.; Tsukagoshi, S.; Hashimoto, S.; Watanabe, S.; Soloshonok, V. A.; Aceña, J. L.; Kitagawa, O. *Tetrahedron* **2012**, *68*, 4013-4017.

12. Singh, T.; Stein, R. G.; Hoops, J. F.; Biel, J. H.; Hoya, W. K.; Cruz, D. R. *J. Med. Chem*. **1971**, *14*, 283-286.

13. Akikusa, N.; Mitsui, K.; Sakamoto, T.; Kikugawa, Y. *Synthesis* **1992**, 1058-1060.

14. Bélanger, G.; Darsigny, V.; Doré, M.; Lévesque, F. *Org. Lett*. **2010**, *12*, 1396-1399.

15. Henry, C.; Bolien, D.; Ibanescu, B.; Bloodworth, S.; Harrowven, D. C.; Zhang, X.; Craven, A.; Sneddon, H. F.; Whitby, R. J. *Eur. J. Org. Chem*. **2015**, 1491-1499.

16. Aubert, C.; Huard-Perrio, C.; Lasne, M. C. *J. Chem. Soc., Perkin Trans. 1,* **1997**, *19*, 2837-2842.

17. Zhang, M. Z.; Guo, Q. H.; Sheng, W. B.; Guo, C. C. *Adv. Synth. Catal.* **2015**, *357*, 2855-2861.

18. Bouasla, R.; Bechlem, K.; Belhani, B. *Orient. J. Chem.* **2017**, *33*, 1454-1460.

19. Ueno, R.; Shirakawa, E. *Org. Biomol. Chem.* **2014**, *12*, 7469-7473.

20. Master, H. E.; Khan, S. I.; Poojari, K. A. *Indian J. Chem. B*. **2008**, *47*, 97-105.

21. Toshimichi, O.; Tomotsugu, A.; Michinori, S. *J. Am. Chem. Soc.* **2010**, *132*, 13191-13193.

22. Funder, E. D.; Trads, J. B.; Gothelf, K. V. *Org. Biomol. Chem.* **2015**, *13*, 185-198.

3. Copies of ^{1}H, ^{13}C and ^{19}F NMR spectra of products

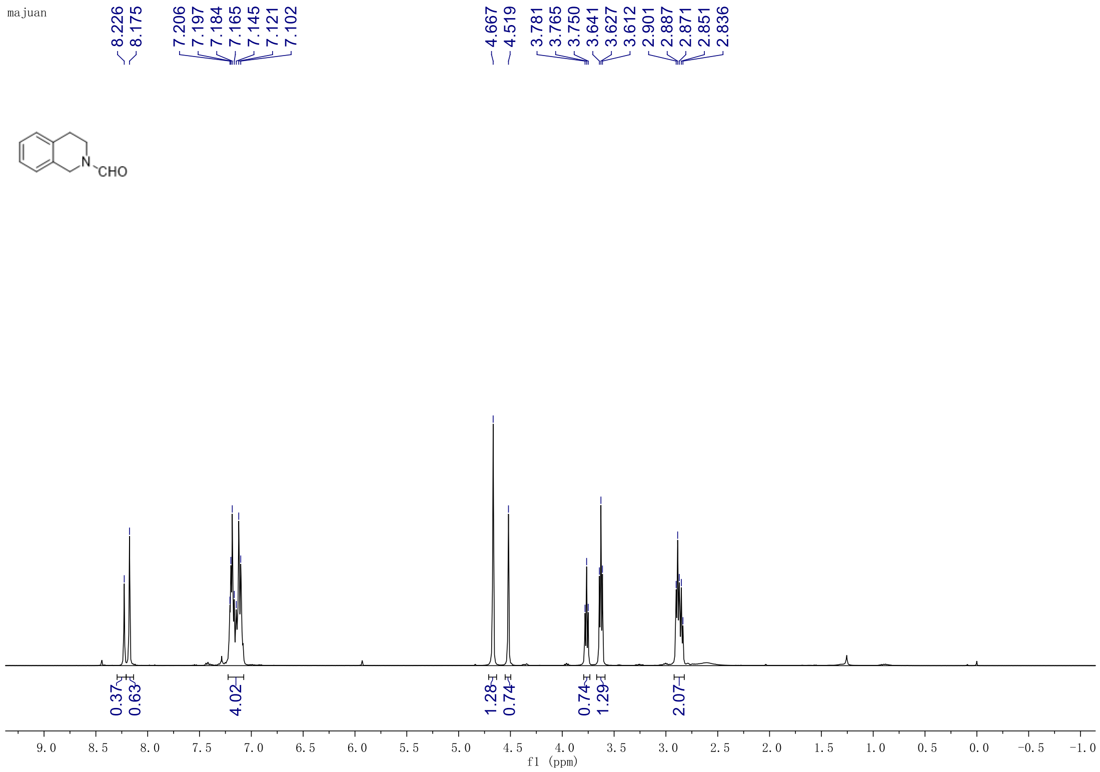

^{1}H NMR spectrum of **2a** (400 MHz, $CDCl_3$)

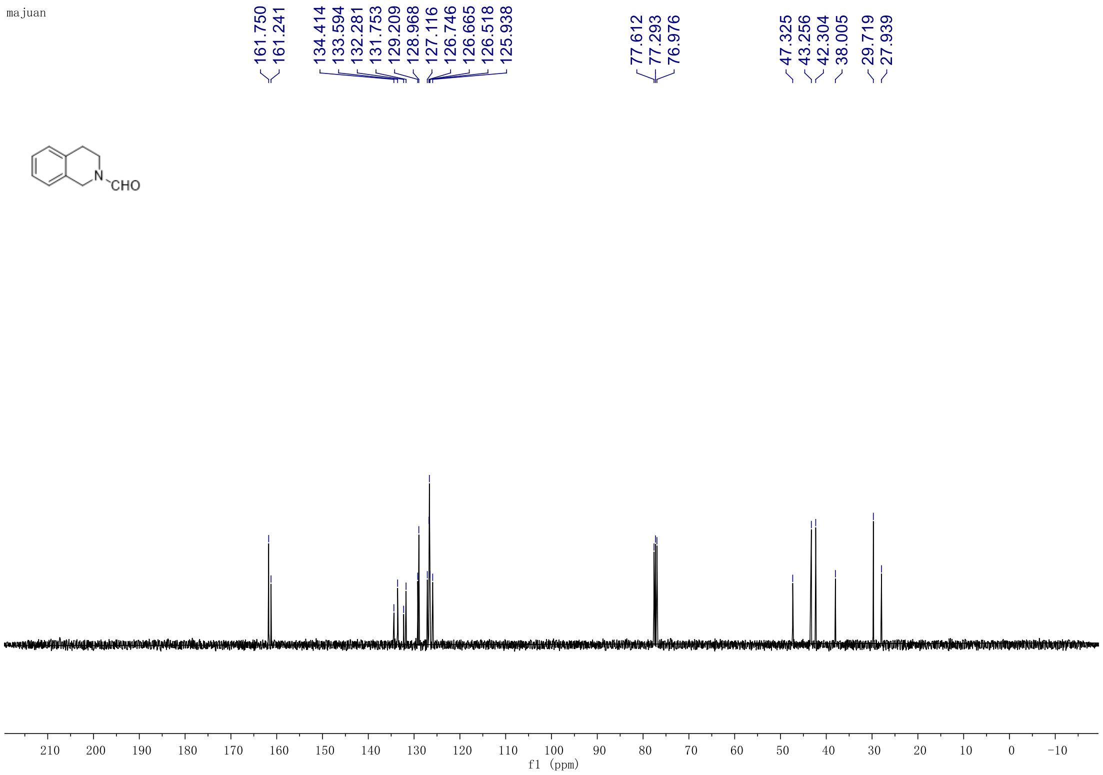

^{13}C NMR spectrum of **2a** (100 MHz, $CDCl_3$)

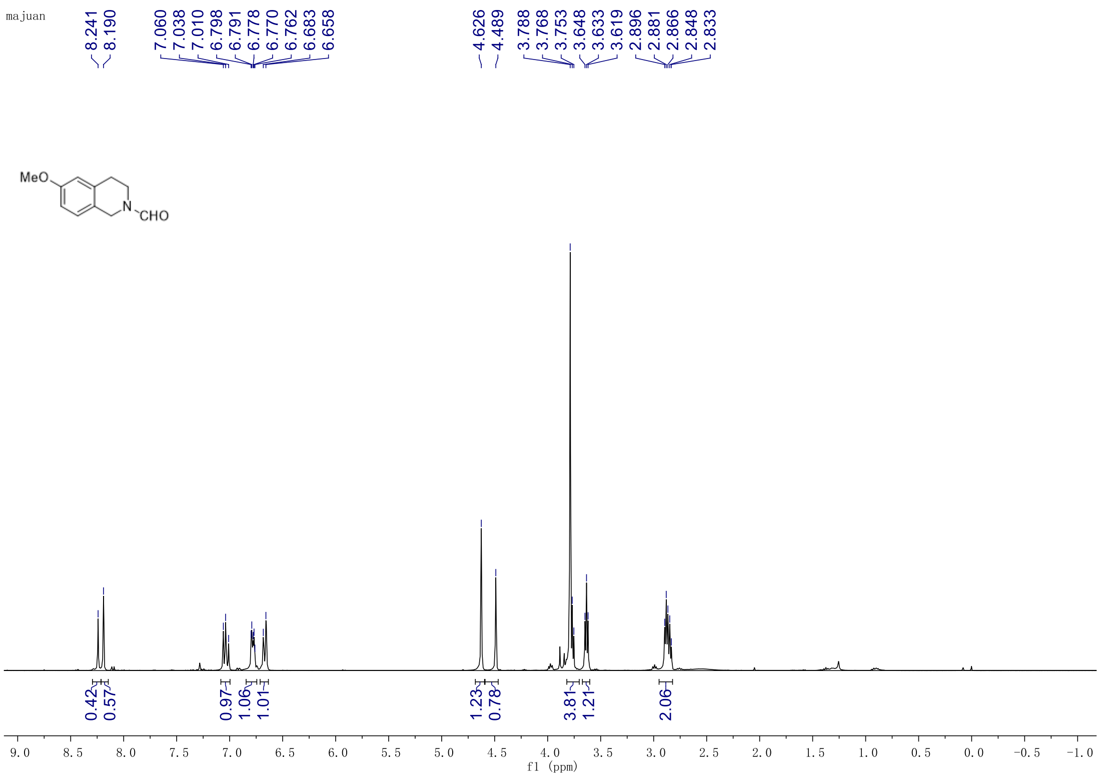

^{1}H NMR spectrum of **2b** (400 MHz, $CDCl_3$)

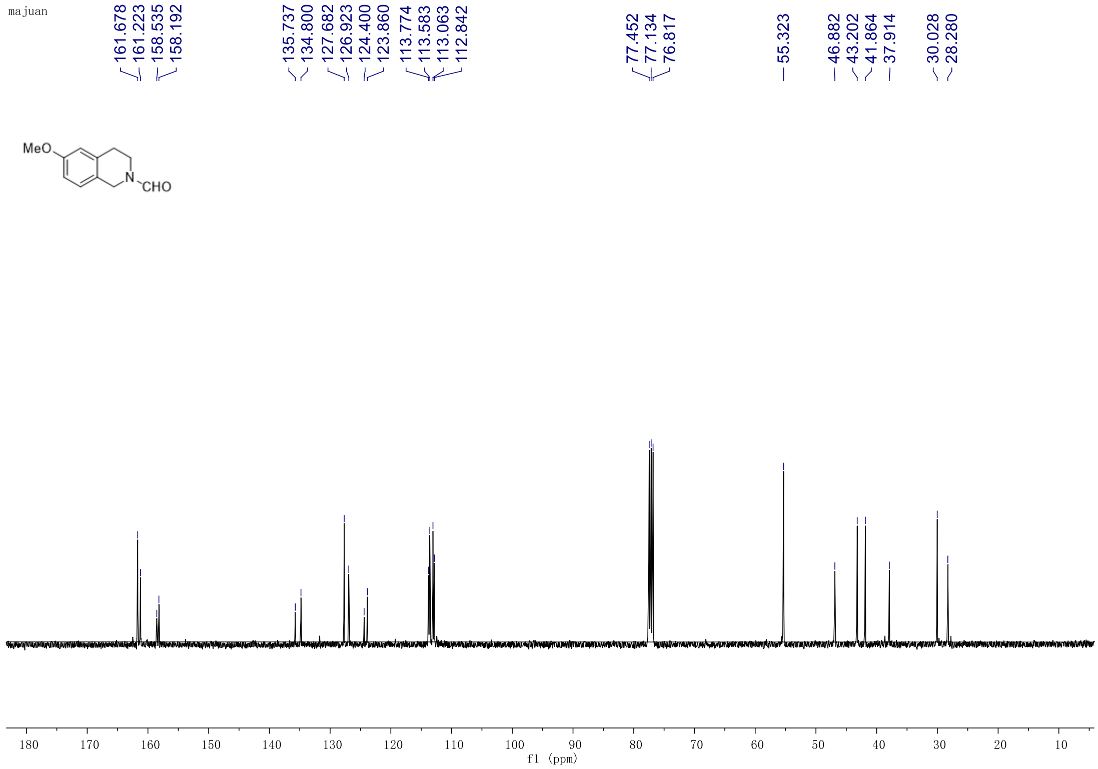

^{13}C NMR spectrum of **2b** (100 MHz, $CDCl_3$)

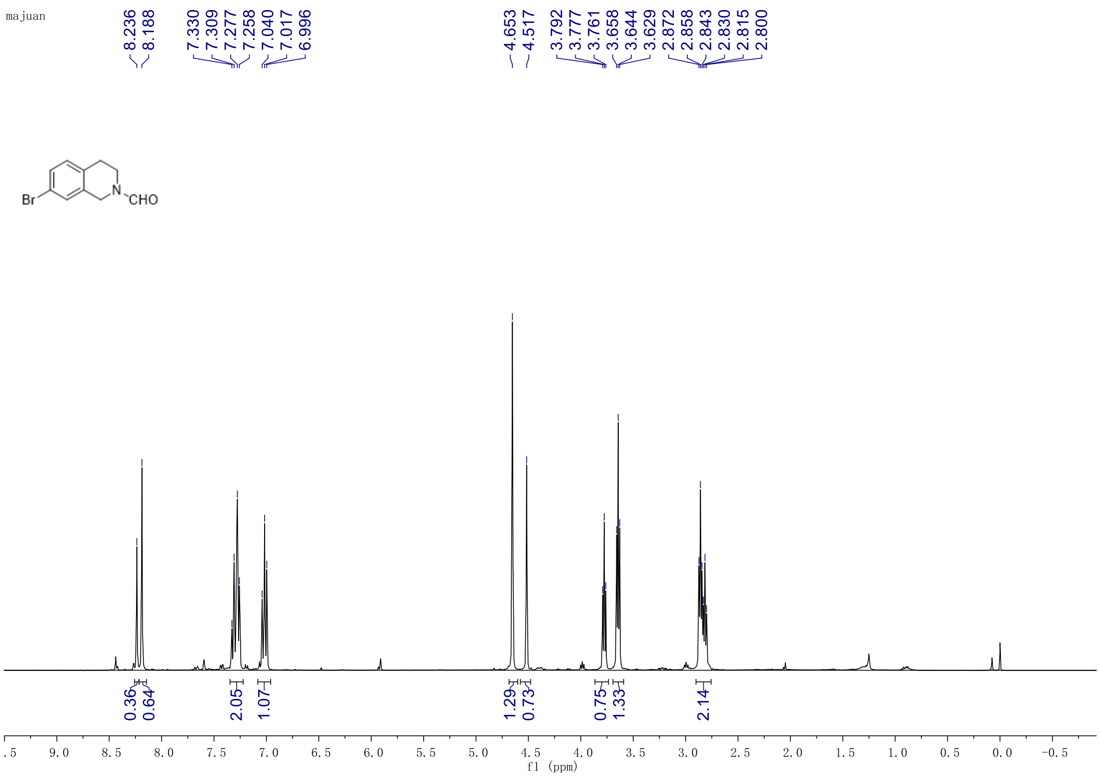

^{1}H NMR spectrum of **2c** (400 MHz, $CDCl_3$)

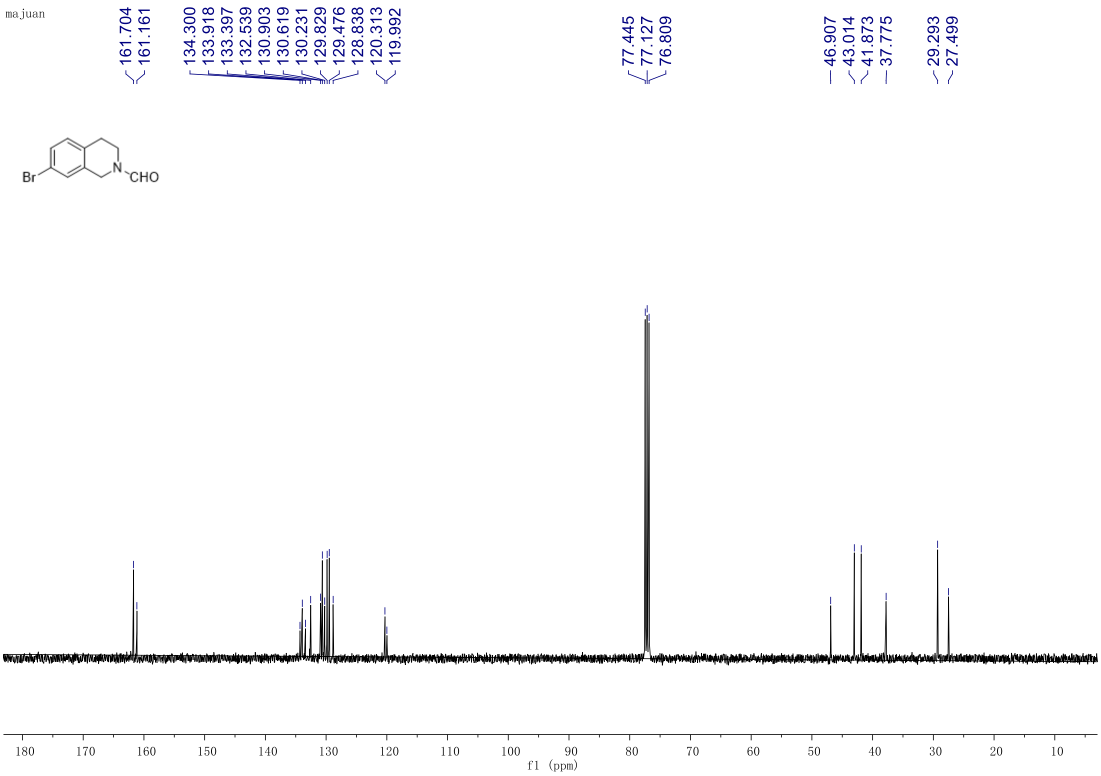

^{13}C NMR spectrum of **2c** (100 MHz, $CDCl_3$)

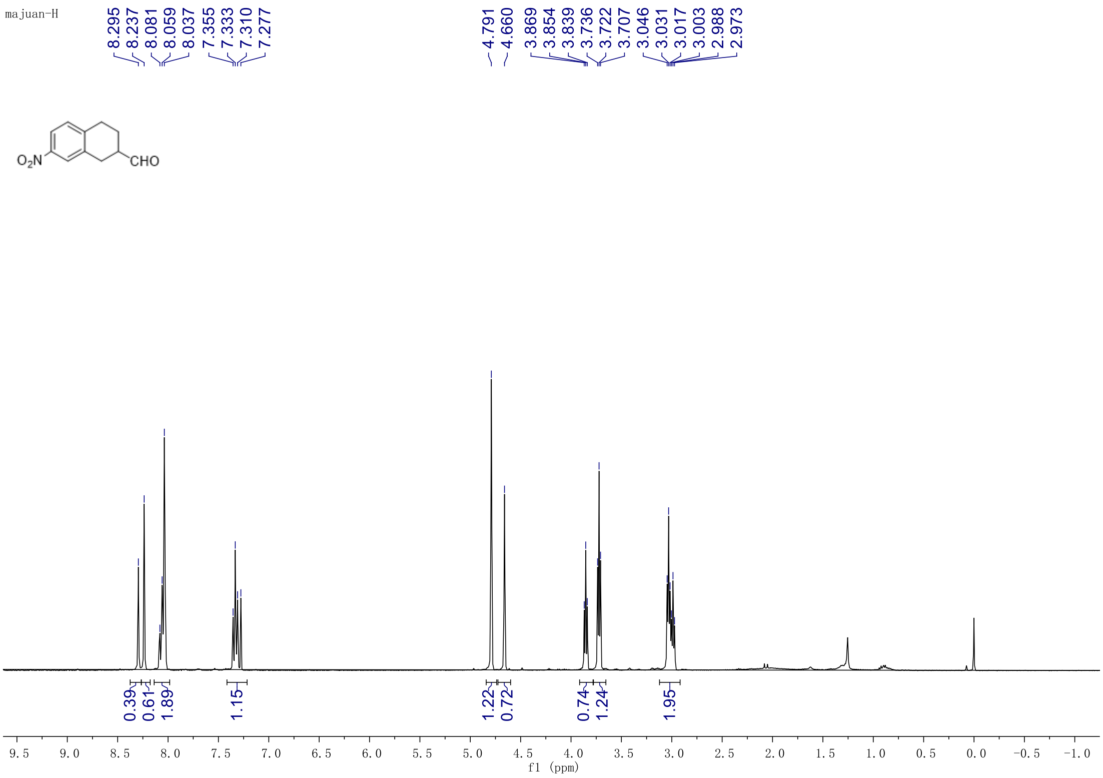

^{1}H NMR spectrum of **2d** (400 MHz, $CDCl_3$)

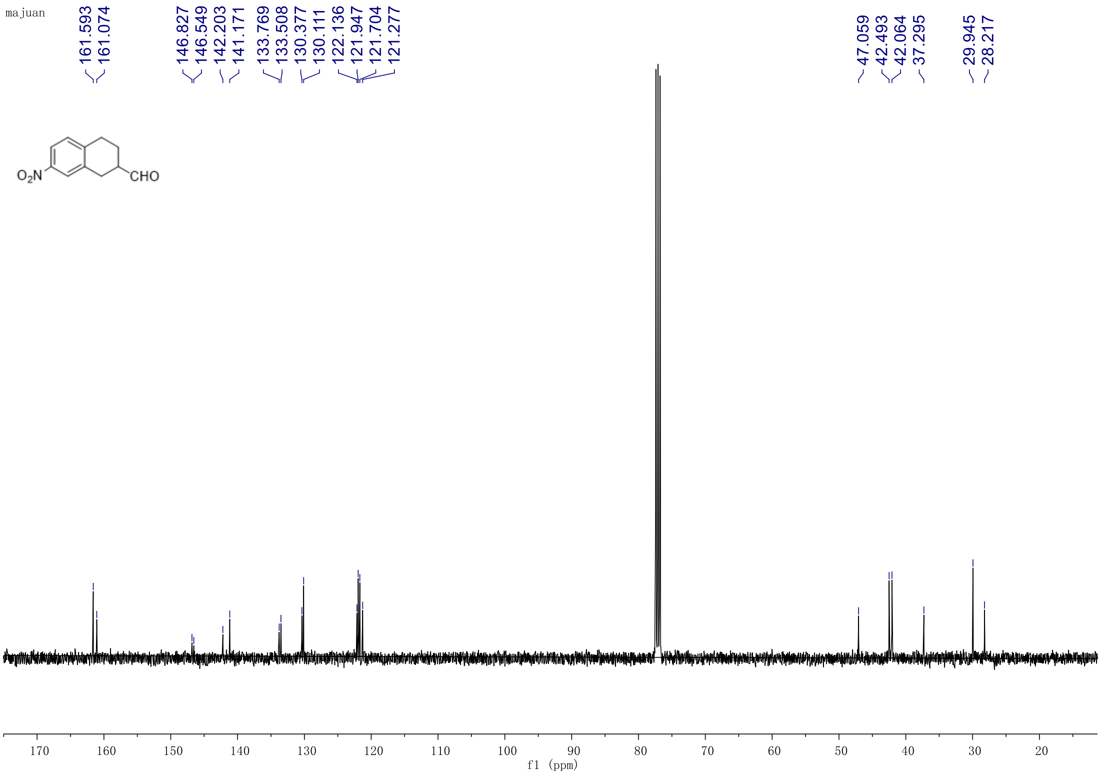

^{13}C NMR spectrum of **2d** (100 MHz, $CDCl_3$)

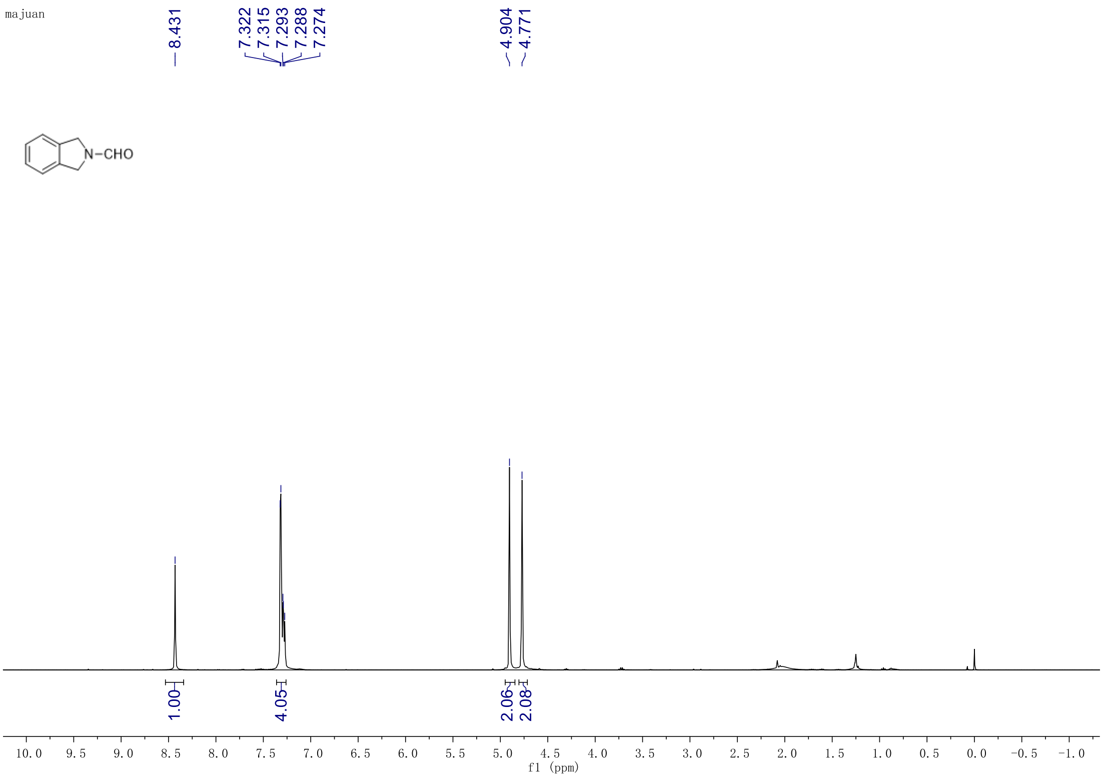

^{1}H NMR spectrum of **2e** (400 MHz, $CDCl_3$)

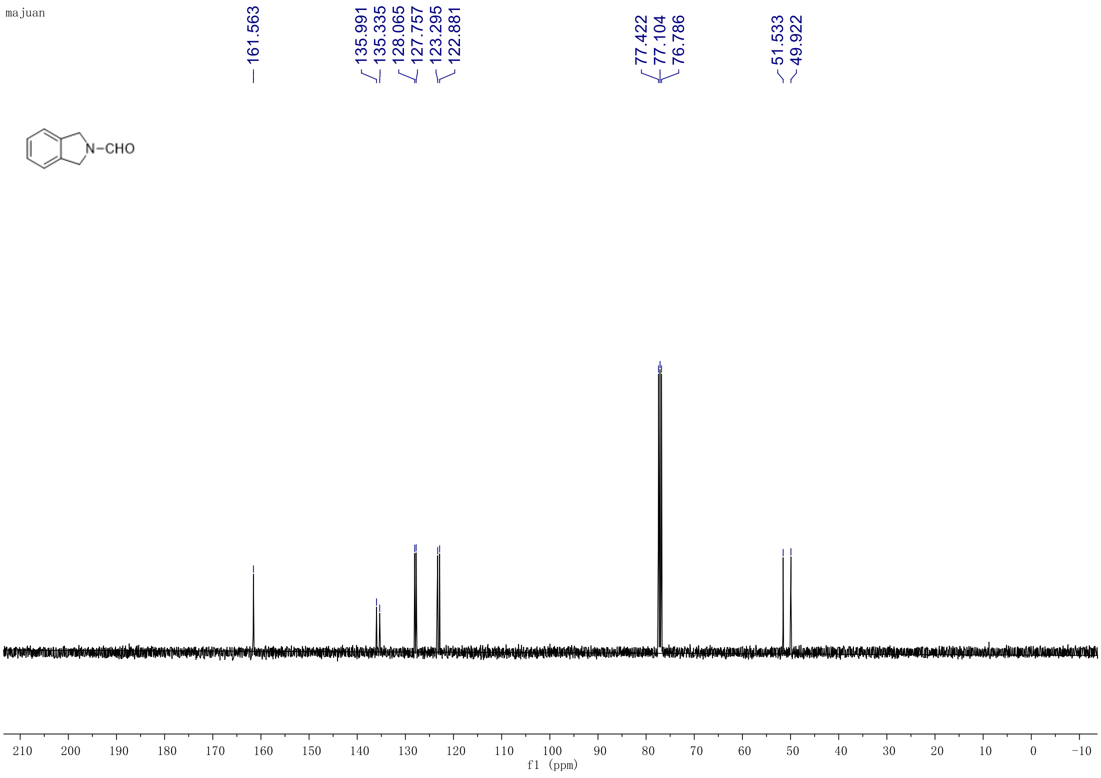

^{13}C NMR spectrum of **2e** (100 MHz, $CDCl_3$)

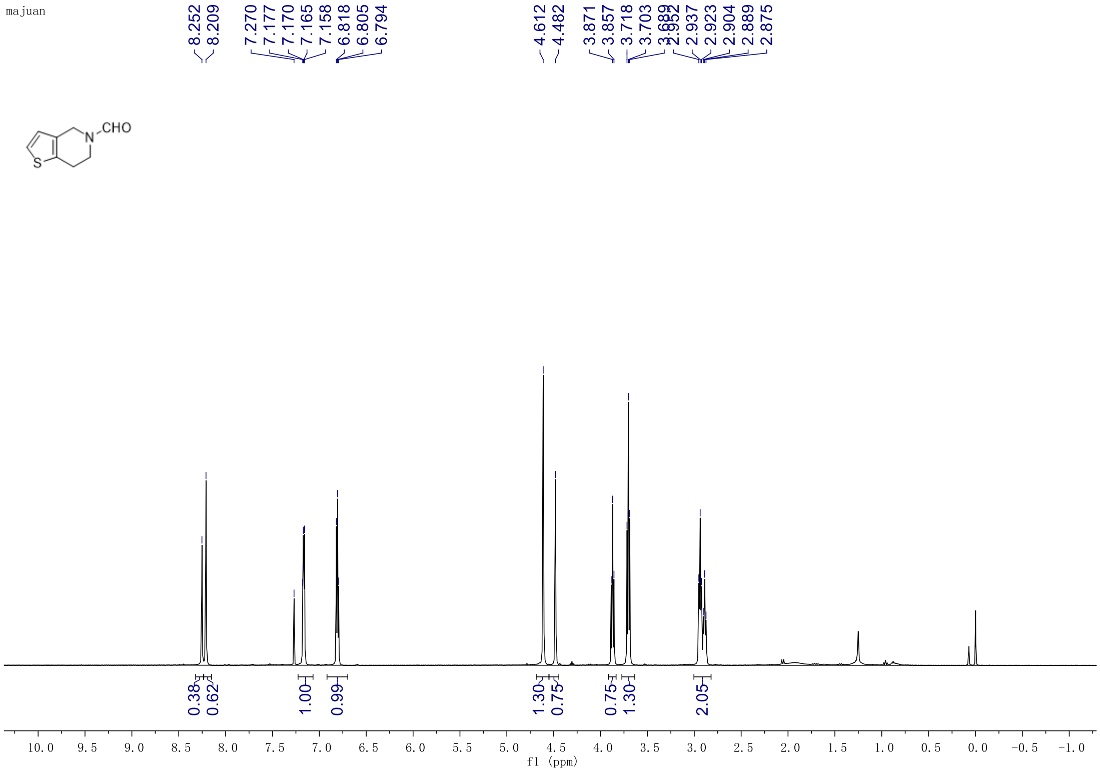

^{1}H NMR spectrum of **2f** (400 MHz, $CDCl_3$)

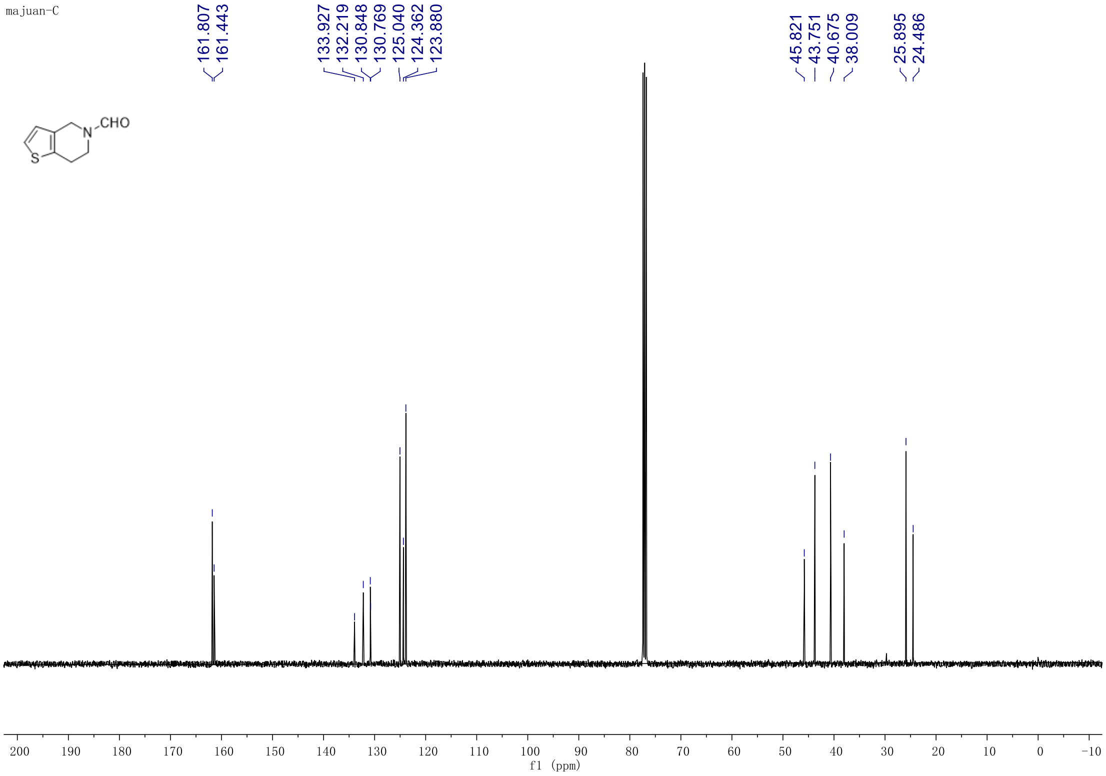

^{13}C NMR spectrum of **2f** (100 MHz, $CDCl_3$)

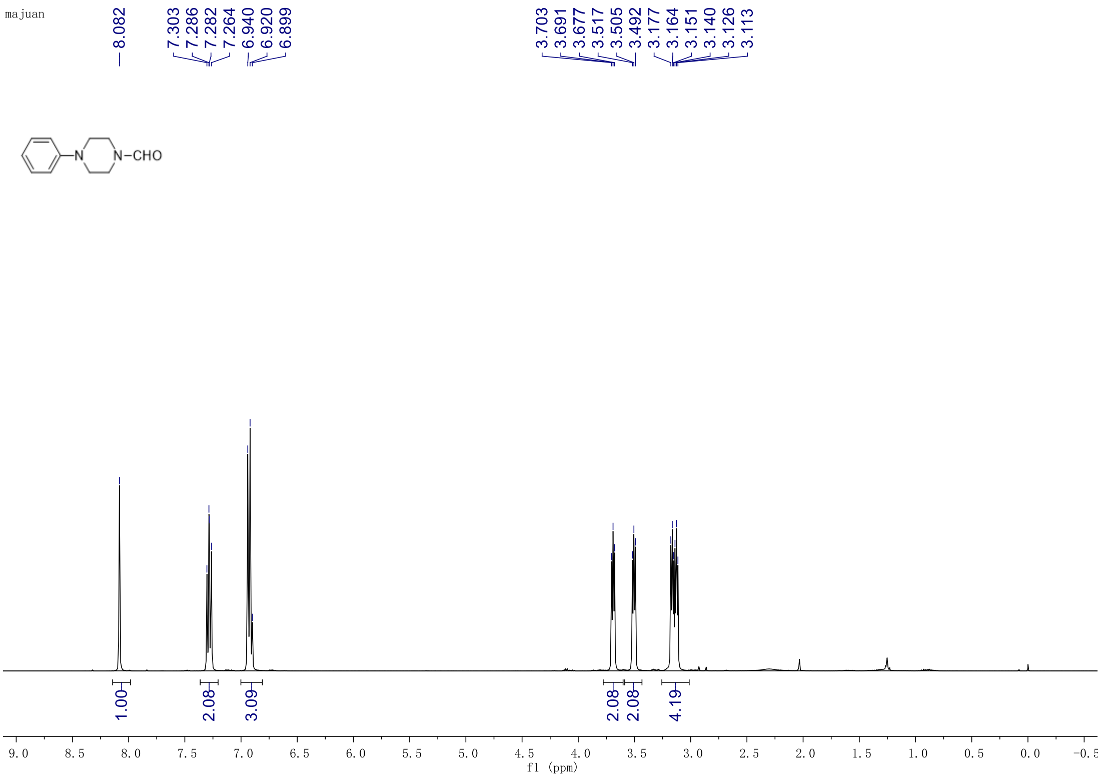

^{1}H NMR spectrum of **2g** (400 MHz, $CDCl_3$)

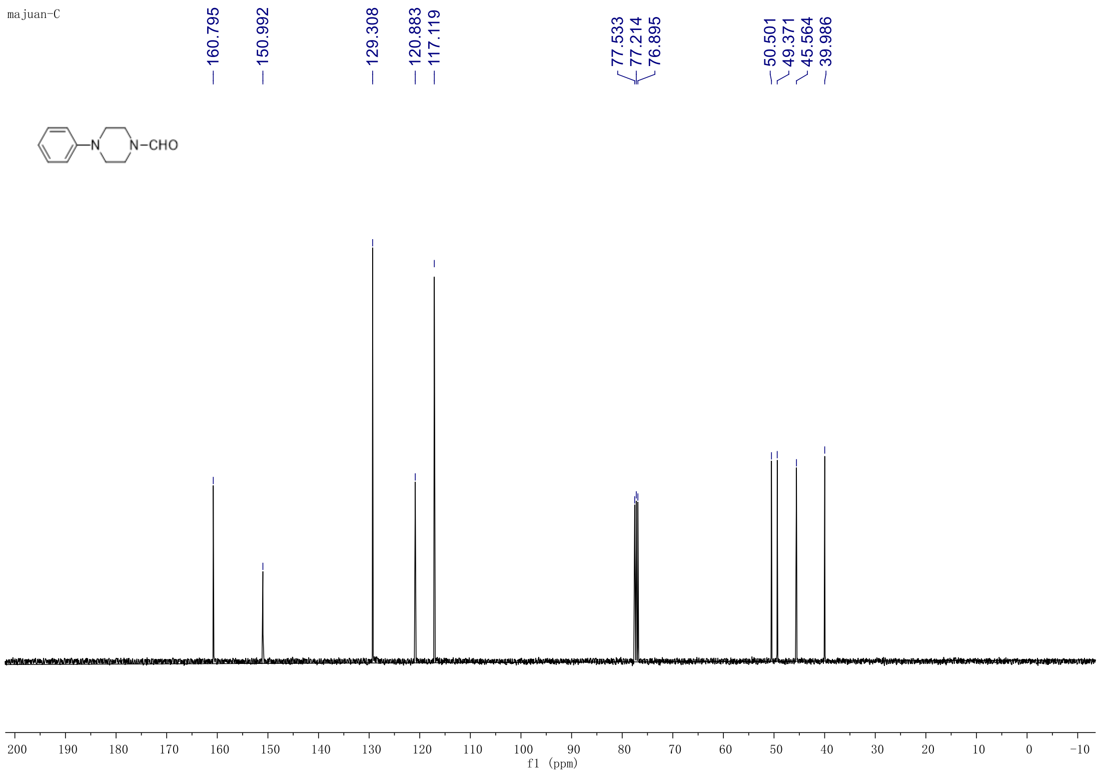

^{13}C NMR spectrum of **2g** (100 MHz, $CDCl_3$)

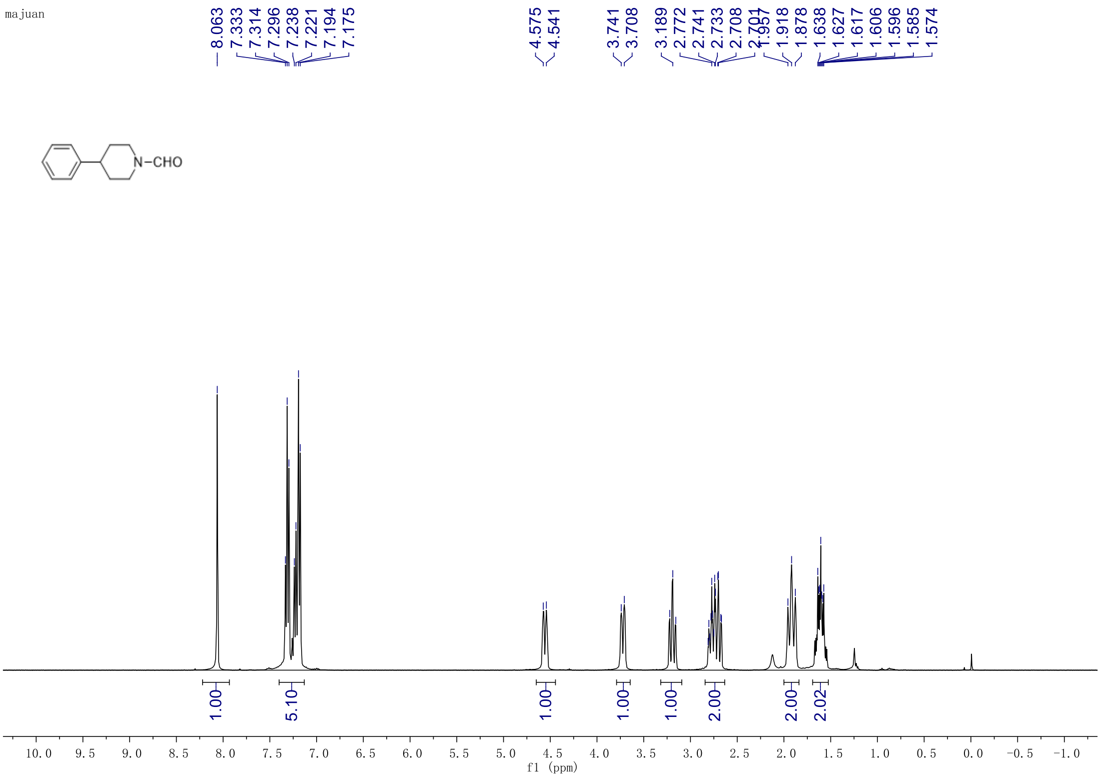

^{1}H NMR spectrum of **2h** (400 MHz, $CDCl_3$)

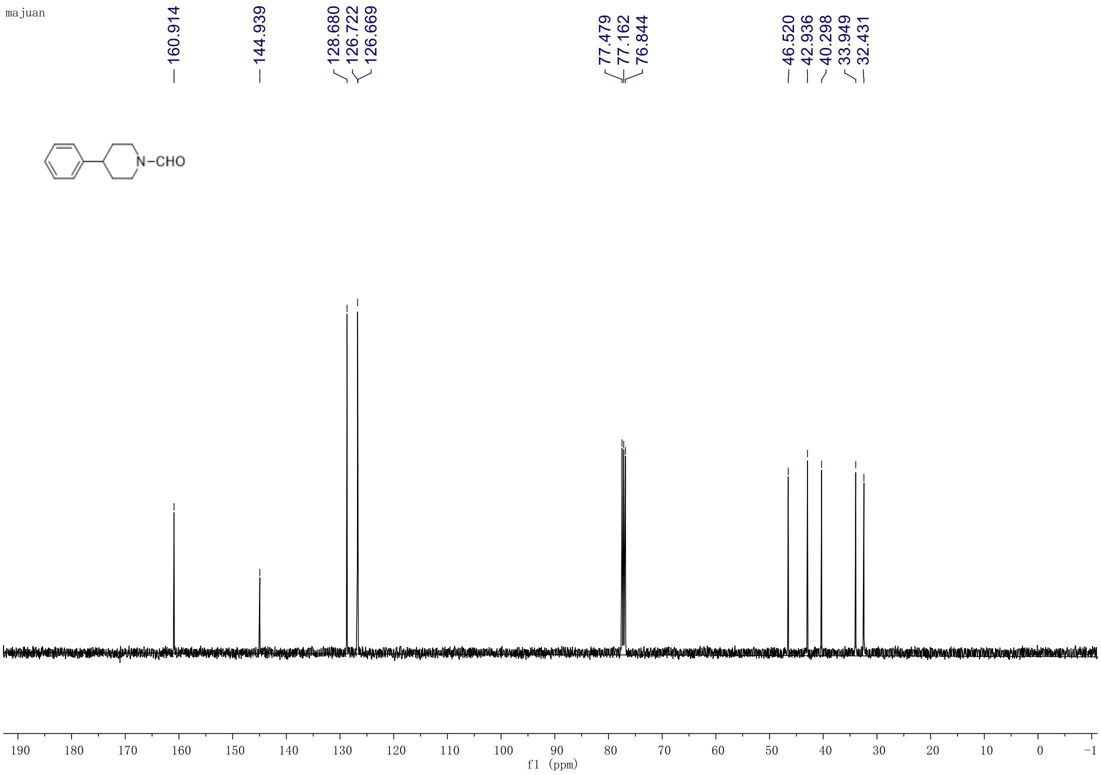

^{13}C NMR spectrum of **2h** (100 MHz, $CDCl_3$)

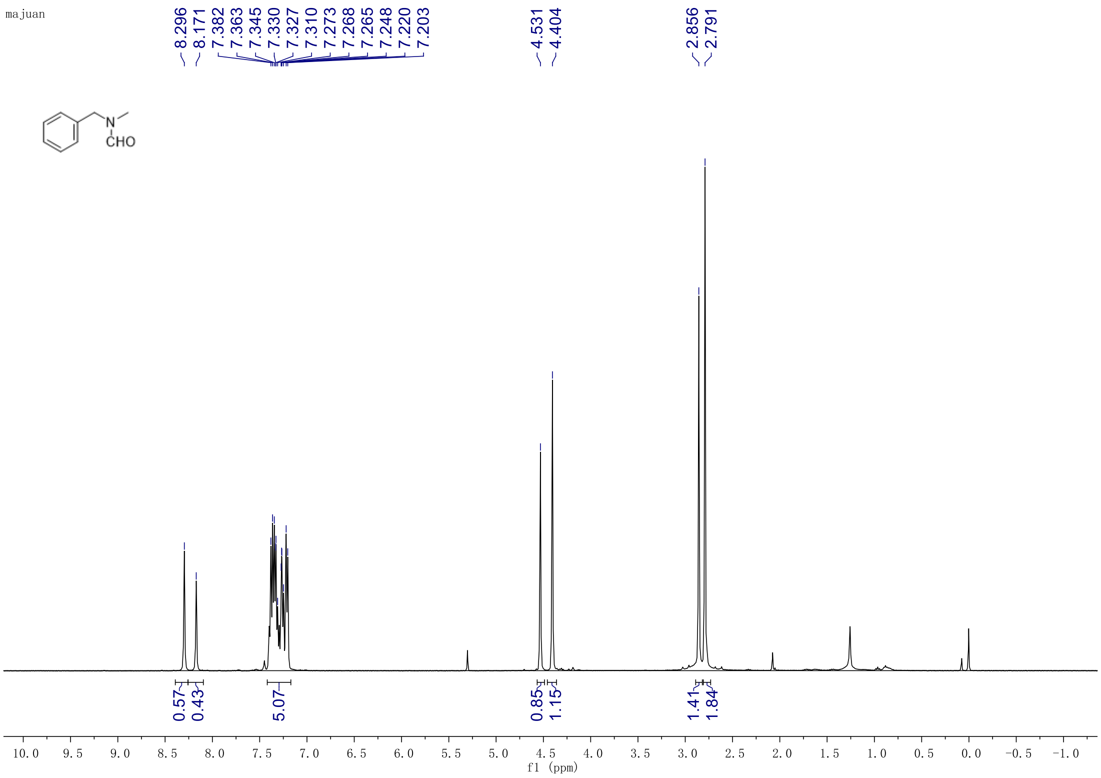

^{1}H NMR spectrum of **2i** (400 MHz, $CDCl_3$)

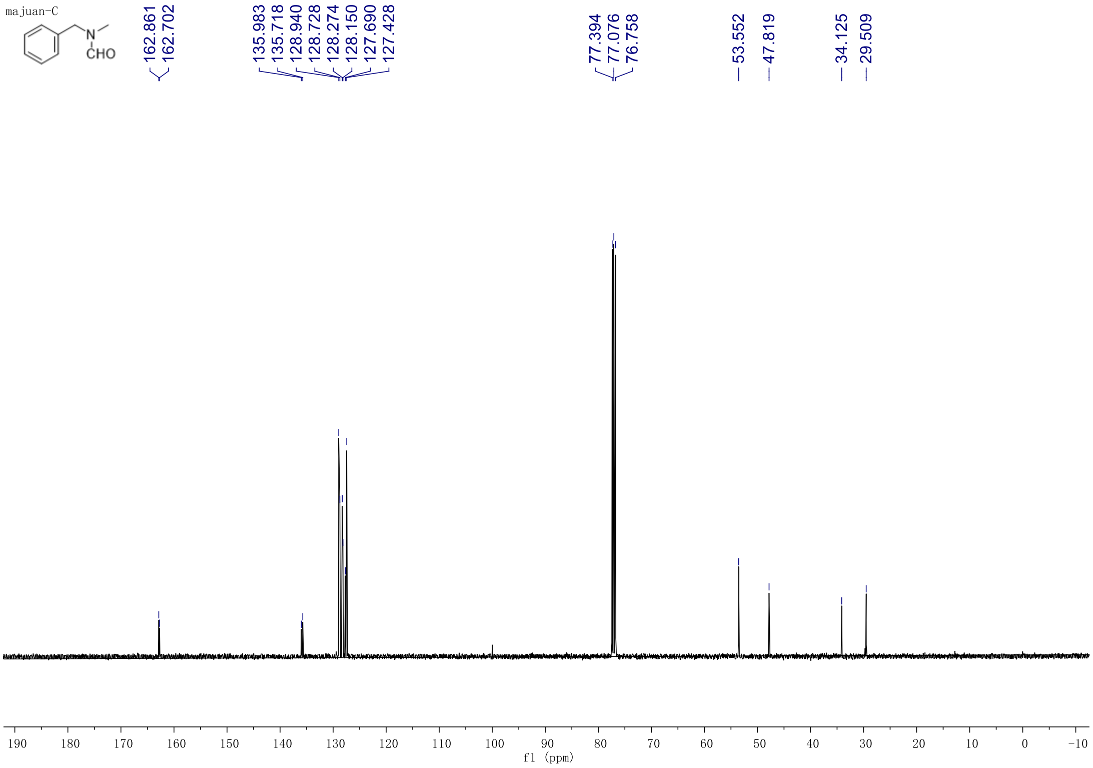

^{13}C NMR spectrum of **2i** (100 MHz, $CDCl_3$)

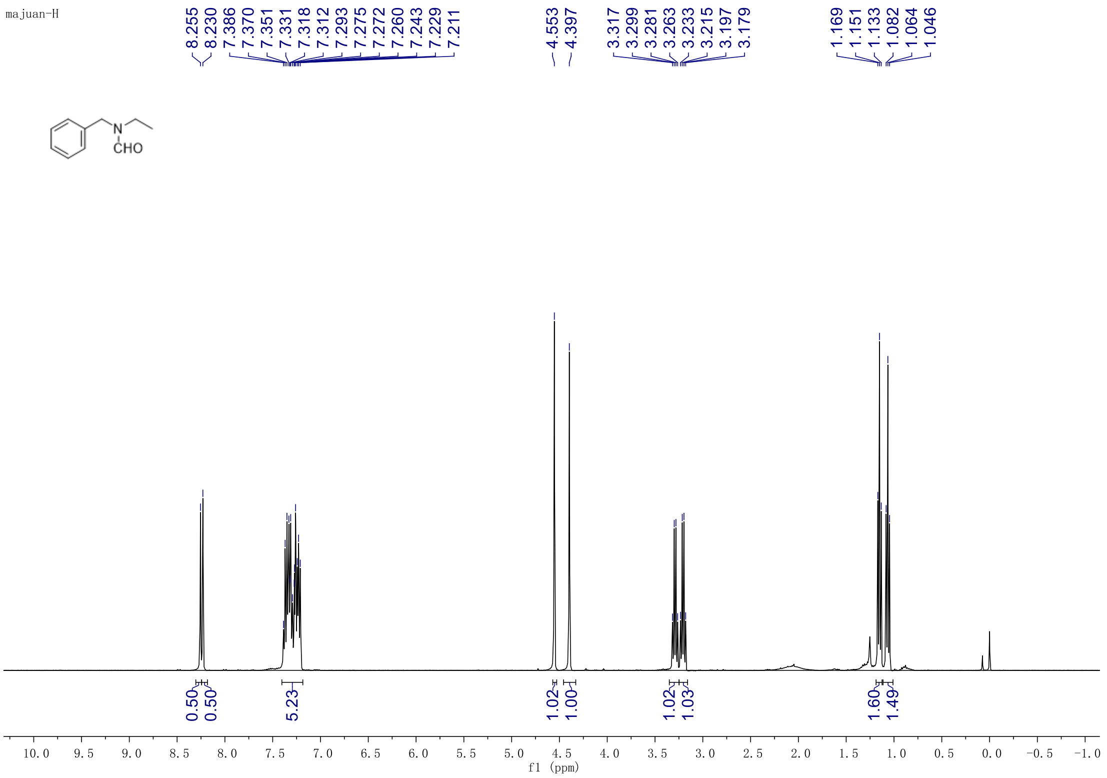

^1H NMR spectrum of **2j** (400 MHz, $CDCl_3$)

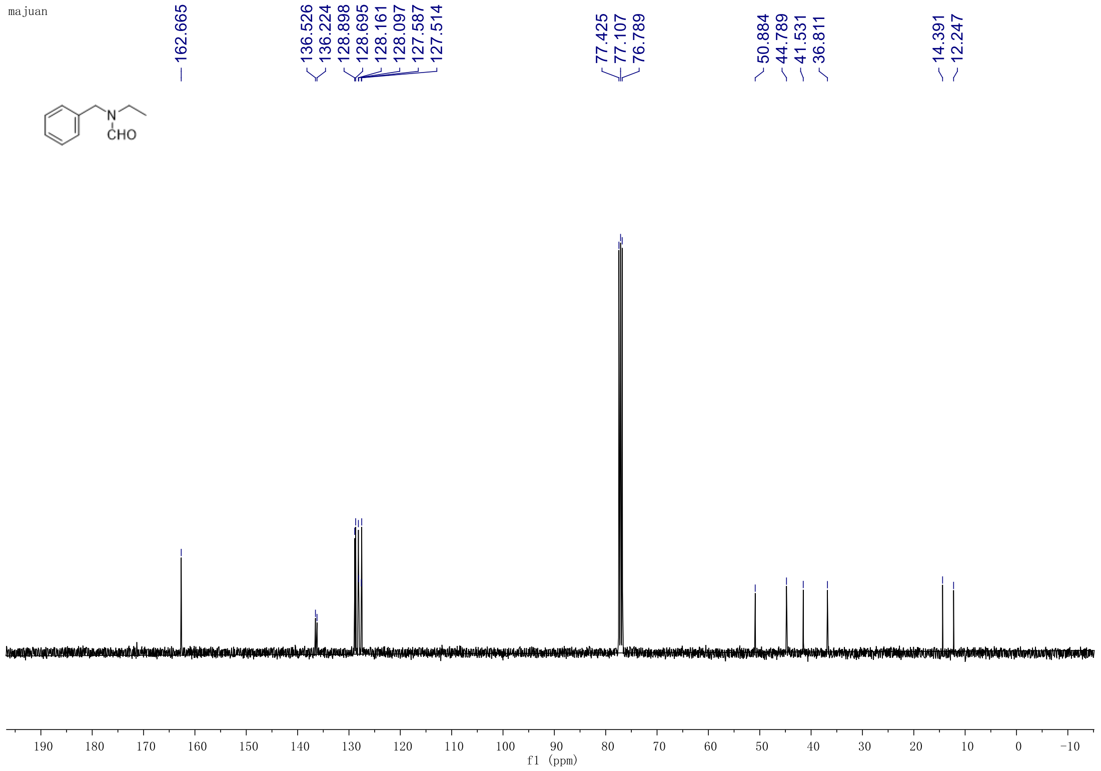

^{13}C NMR spectrum of **2j** (100 MHz, $CDCl_3$)

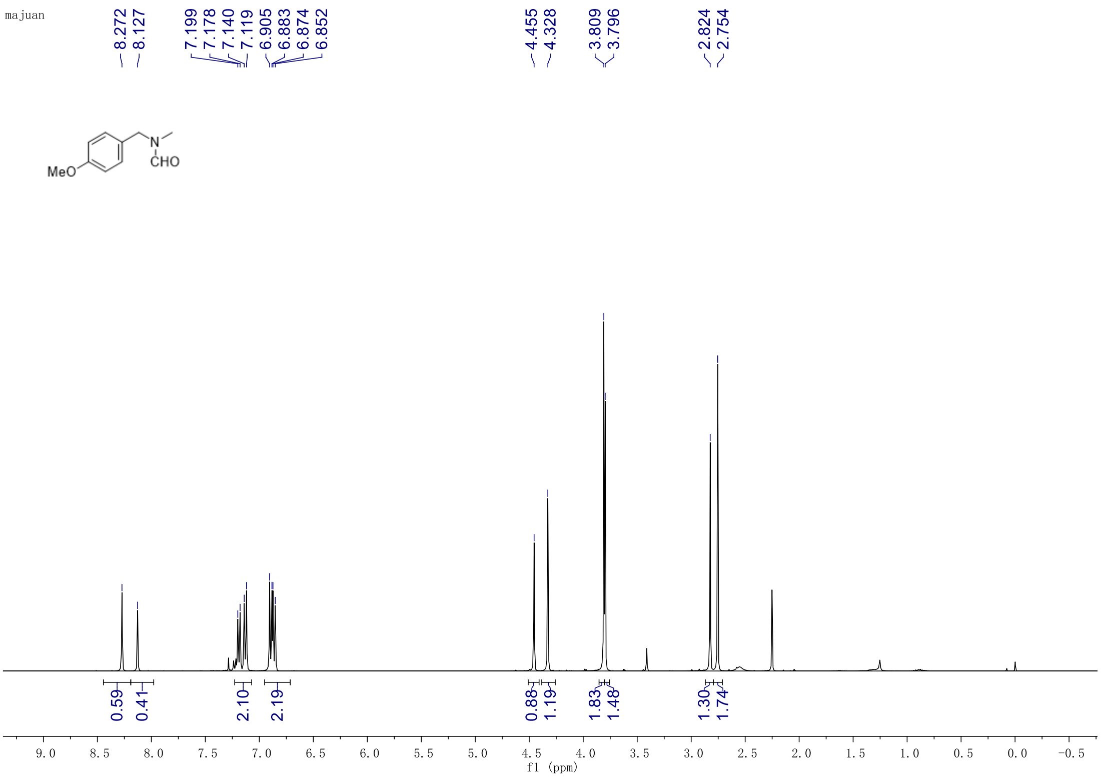

^{1}H NMR spectrum of **2k** (400 MHz, $CDCl_3$)

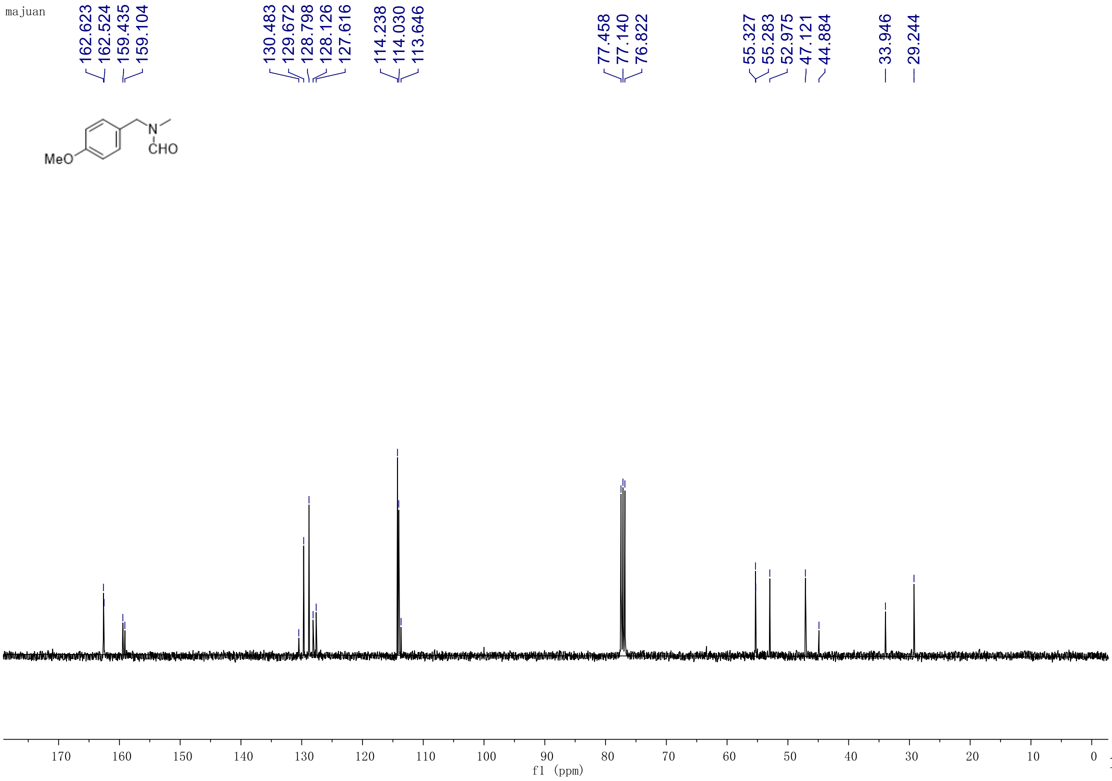

^{13}C NMR spectrum of **2k** (100 MHz, $CDCl_3$)

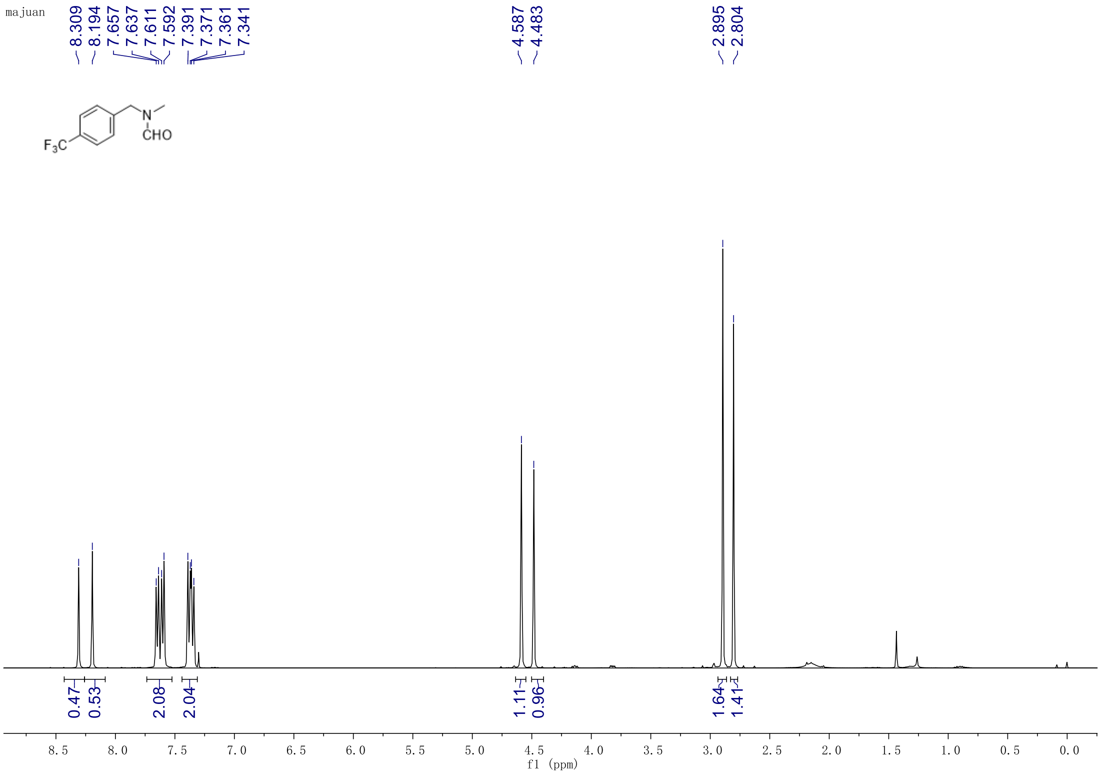

^{1}H NMR spectrum of **2l** (400 MHz, $CDCl_3$)

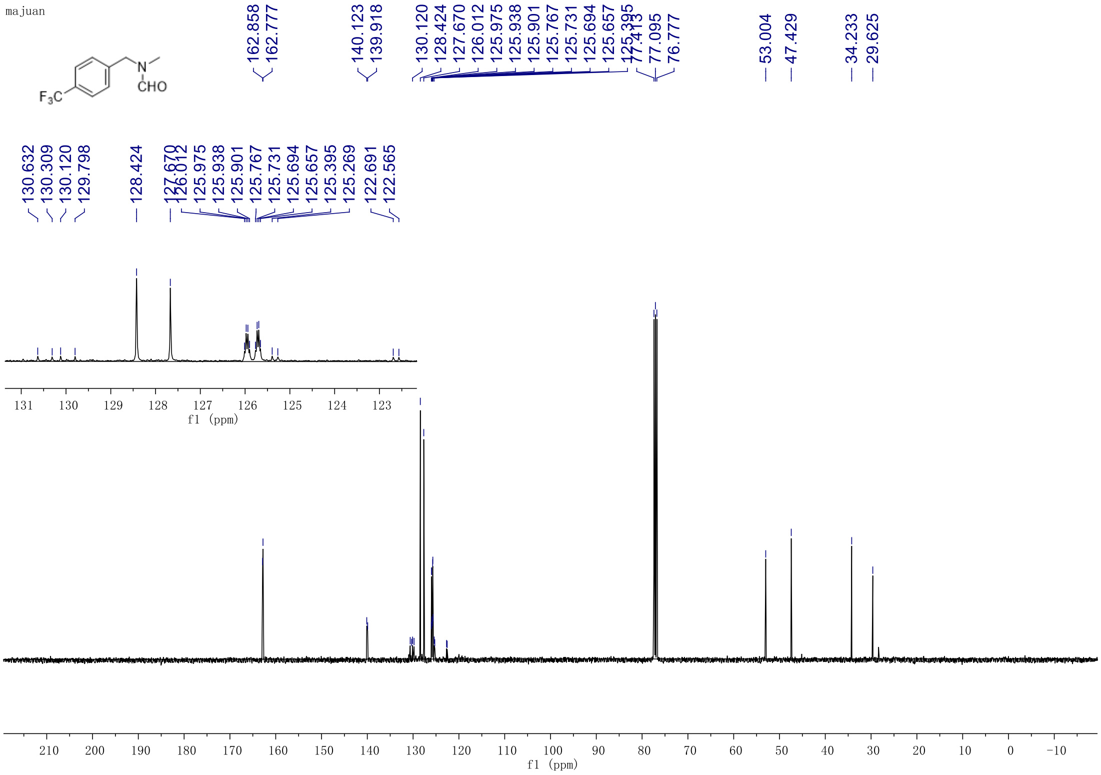

^{13}C NMR spectrum of **2l** (100 MHz, $CDCl_3$)

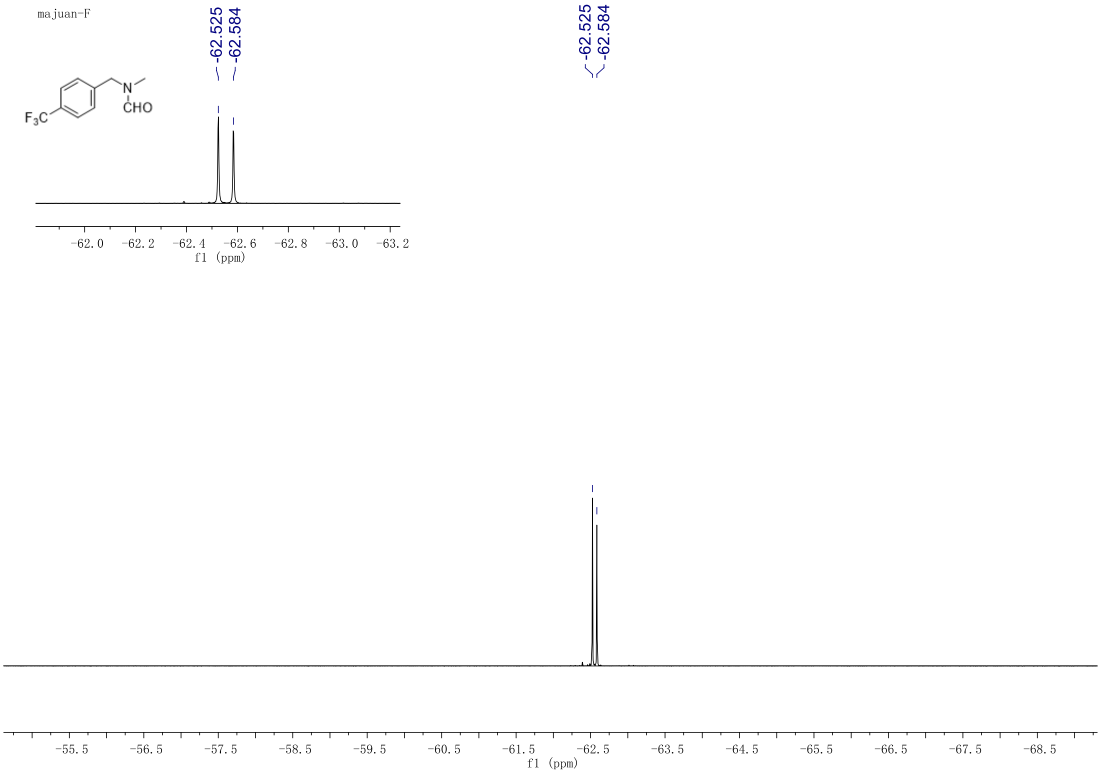

^{19}F NMR spectrum of **2l** (377 MHz, $CDCl_3$)

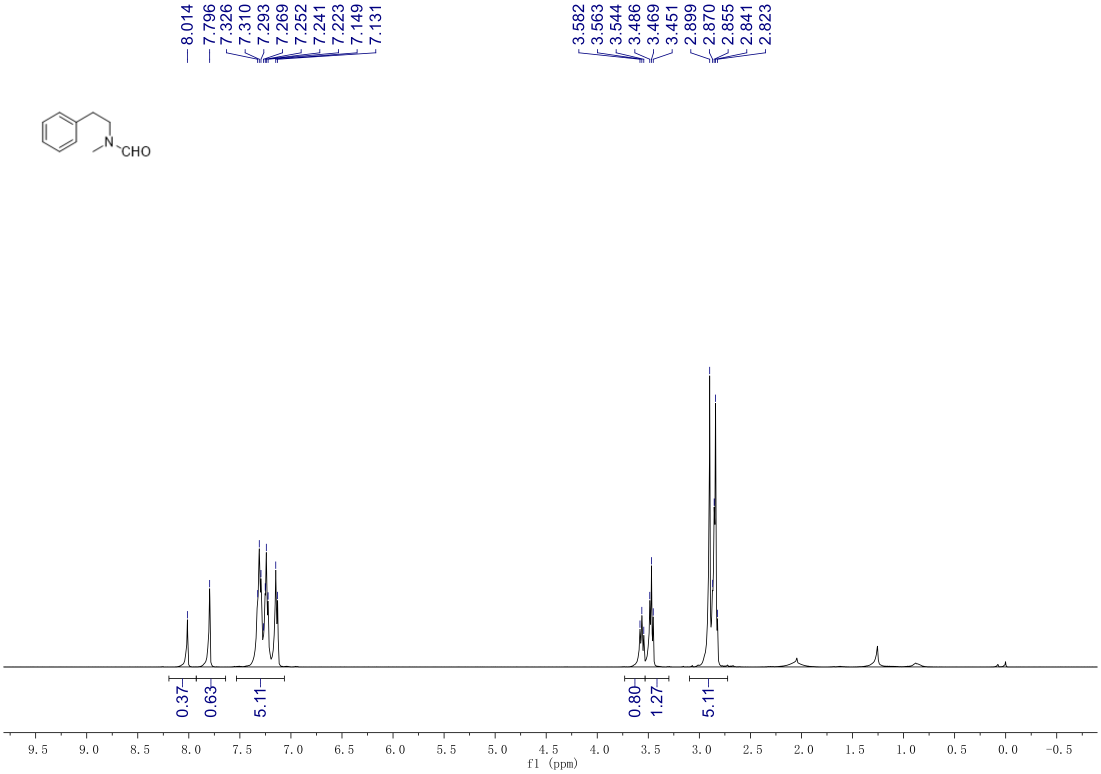

^{1}H NMR spectrum of **2m** (400 MHz, $CDCl_3$)

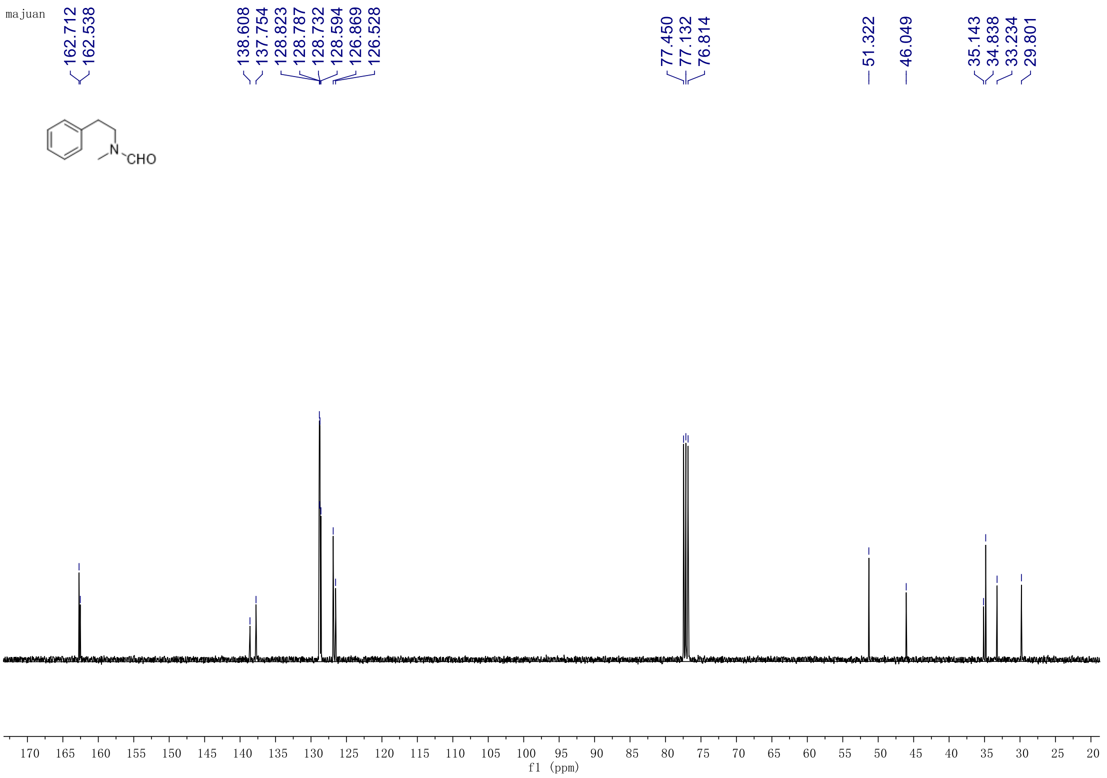

^{13}C NMR spectrum of **2m** (100 MHz, $CDCl_3$)

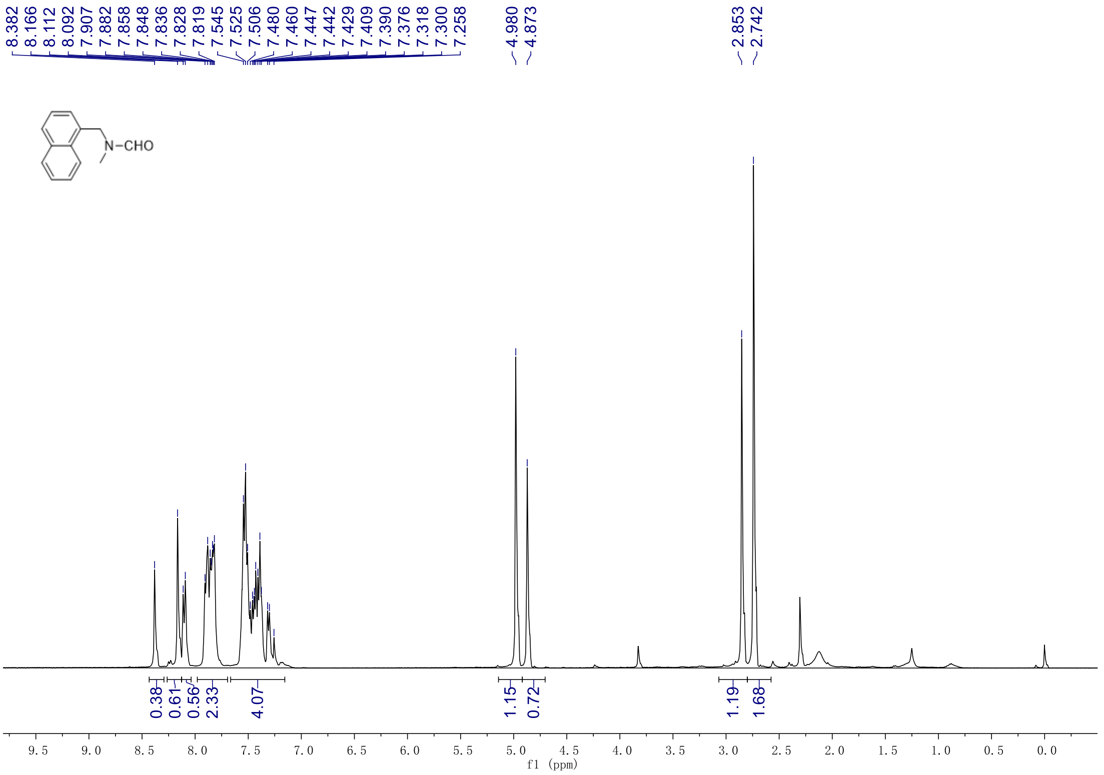

^{1}H NMR spectrum of **2n** (400 MHz, $CDCl_3$)

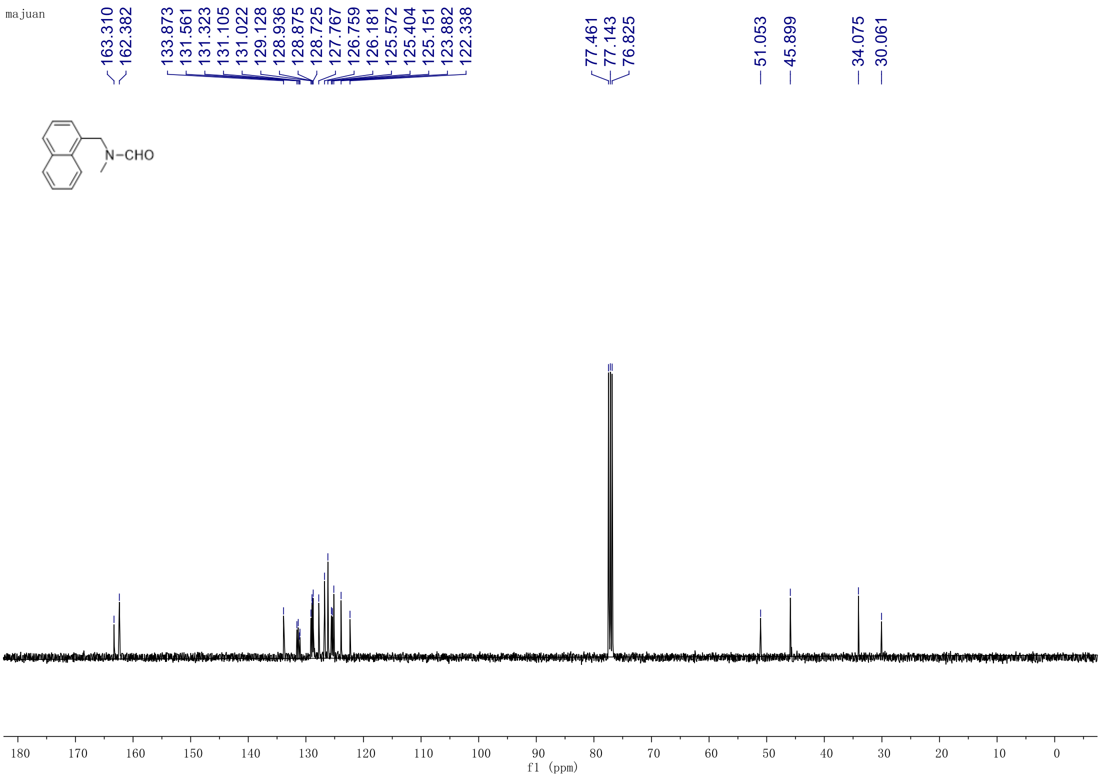

^{13}C NMR spectrum of **2n** (100 MHz, $CDCl_3$)

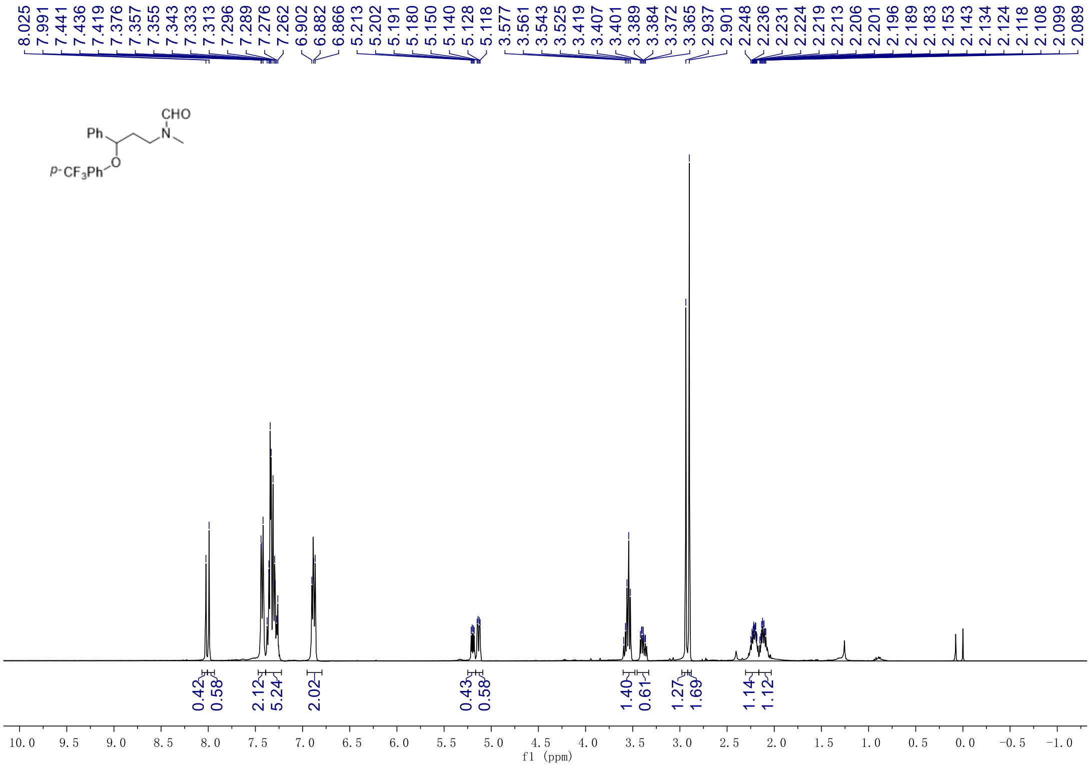

^{1}H NMR spectrum of **2o** (400 MHz, $CDCl_3$)

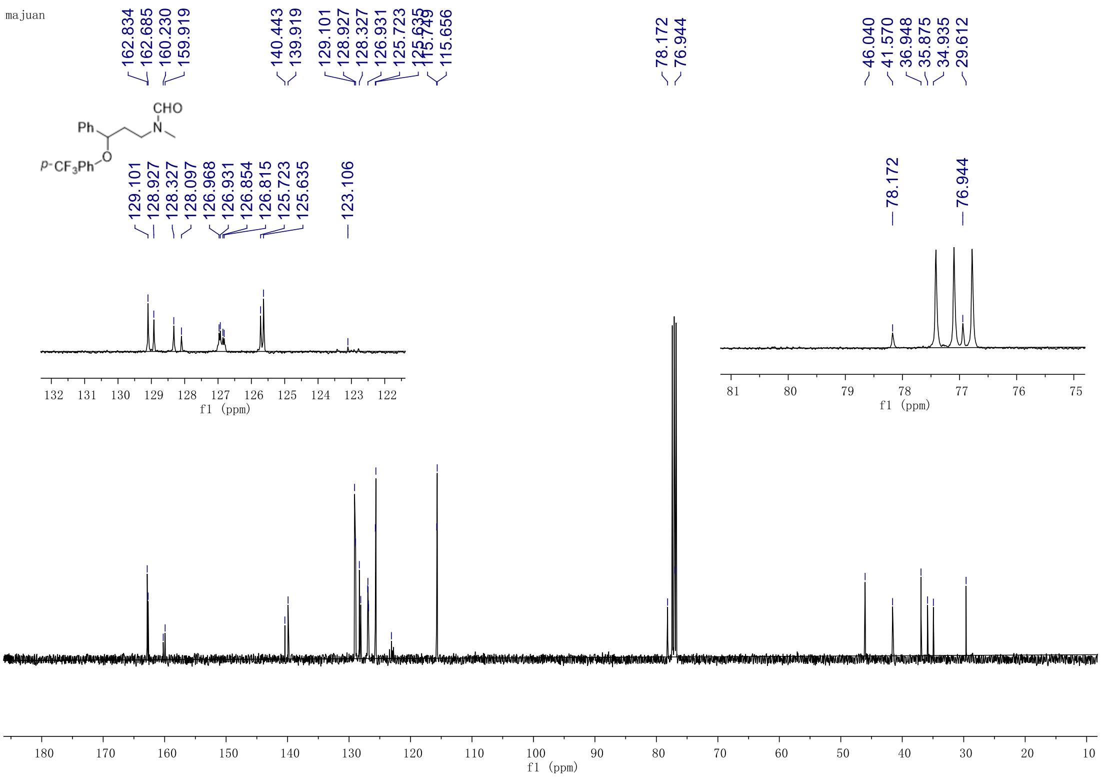

^{13}C NMR spectrum of **2o** (100 MHz, $CDCl_3$)

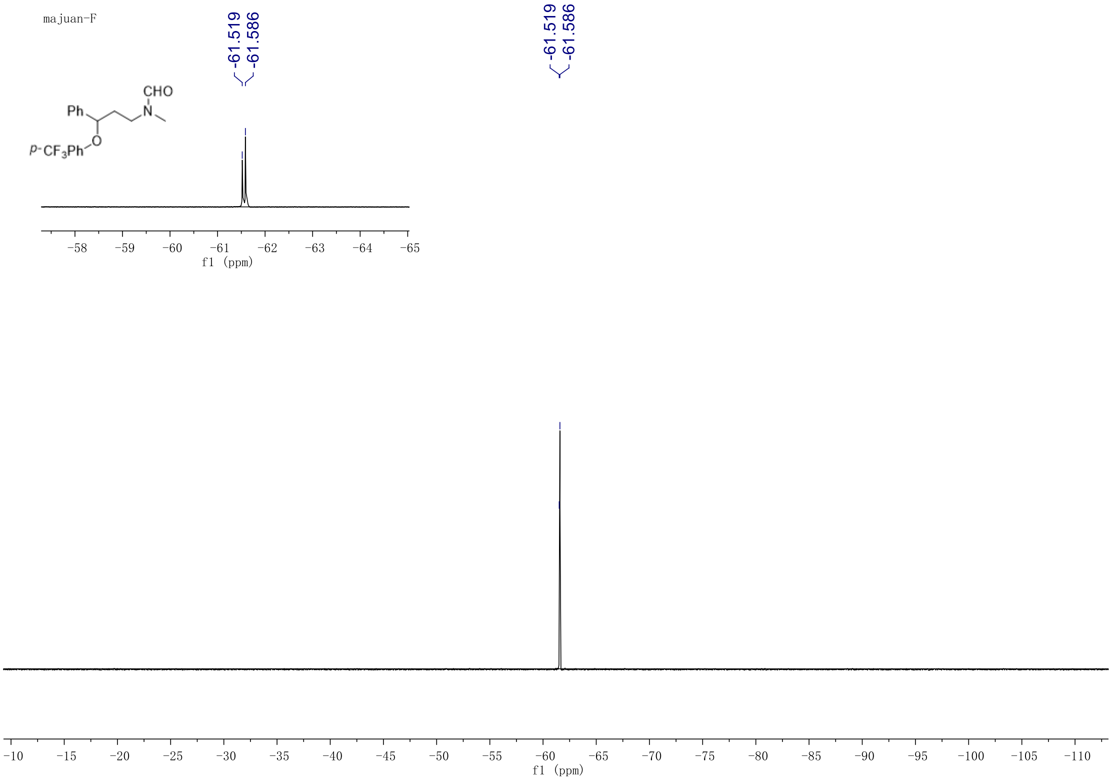

^{19}F NMR spectrum of **2o** (377 MHz, $CDCl_3$)

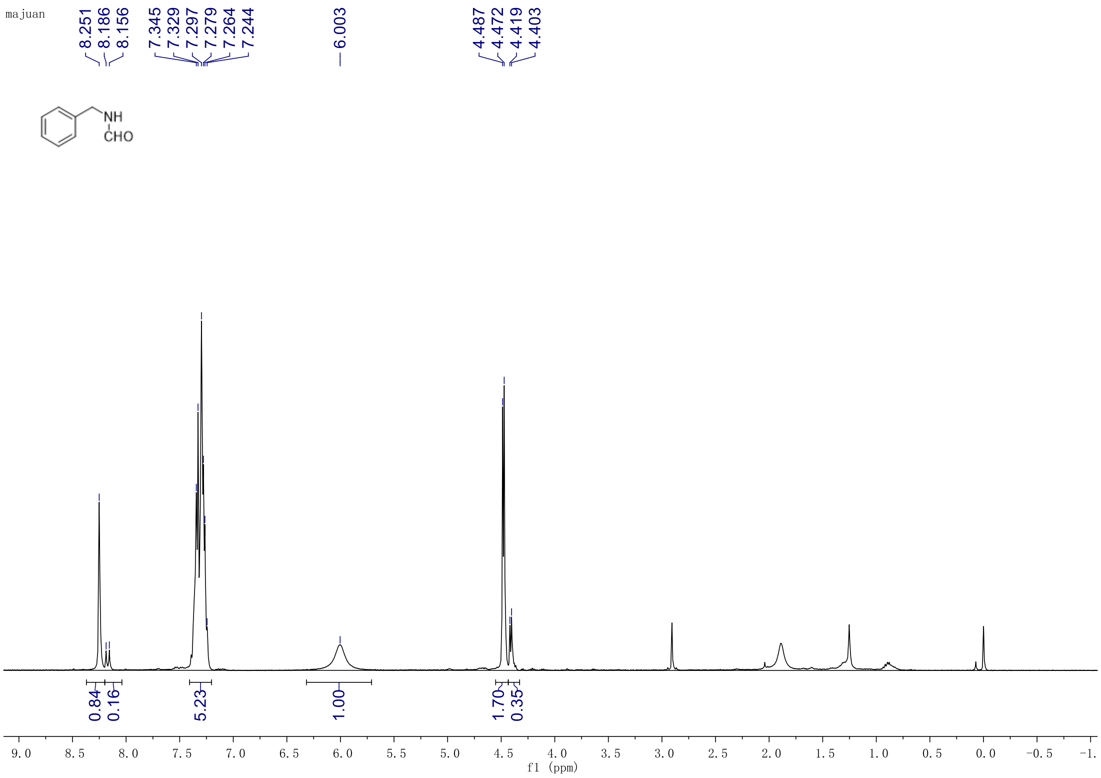

^{1}H NMR spectrum of **2p** (400 MHz, $CDCl_3$)

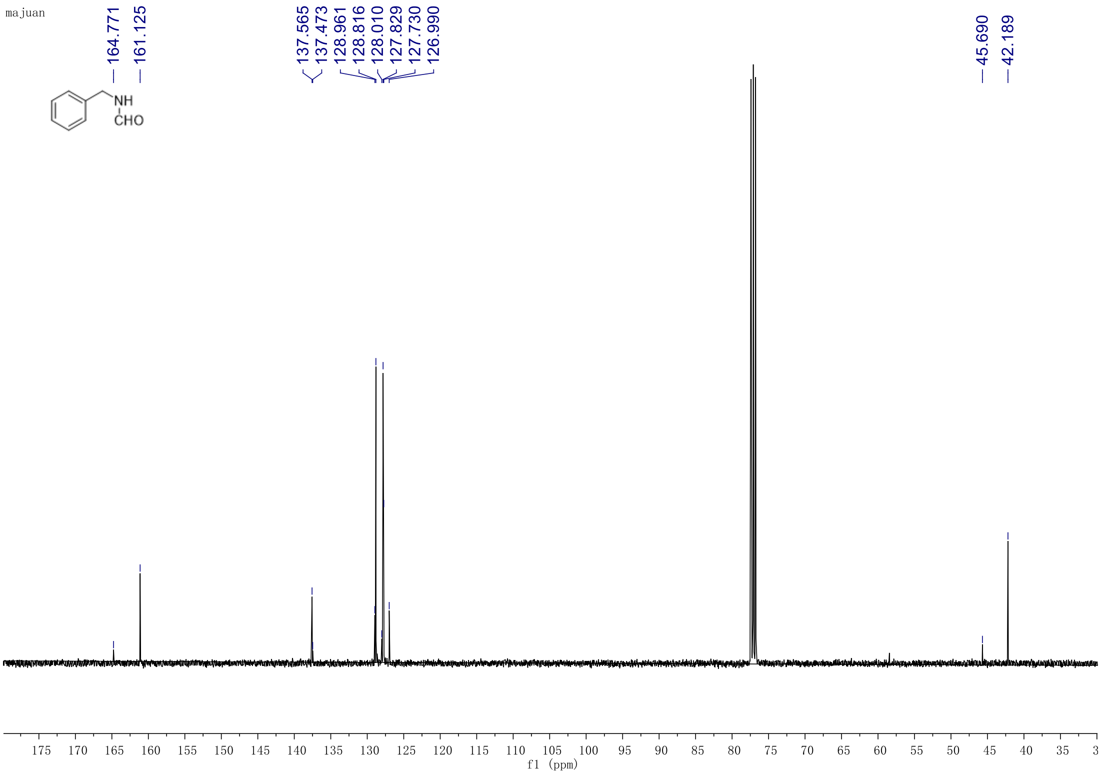

^{13}C NMR spectrum of **2p** (100 MHz, $CDCl_3$)

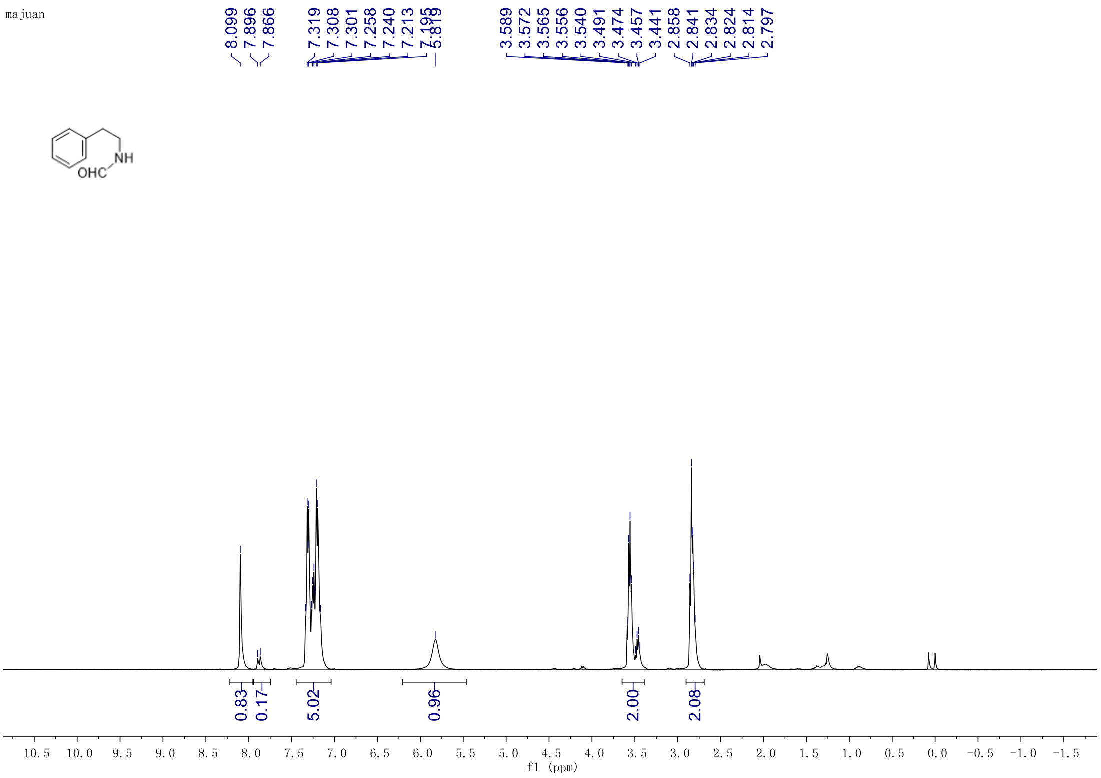

^{1}H NMR spectrum of **2q** (400 MHz, $CDCl_3$)

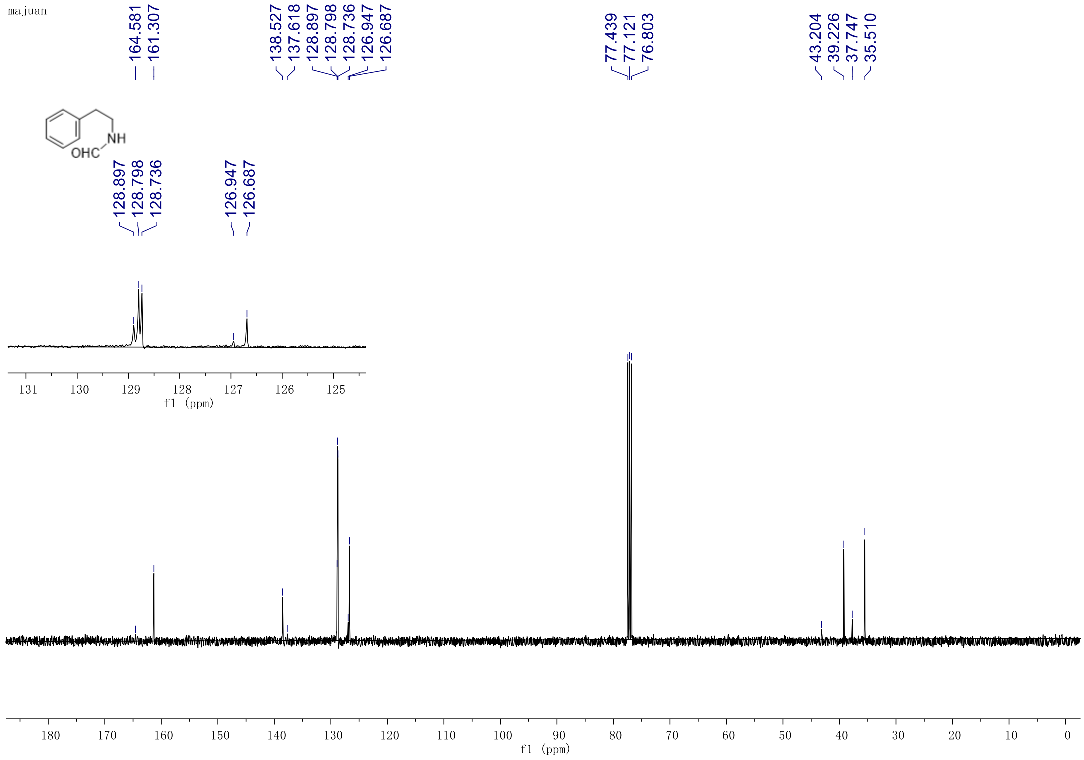

^{13}C NMR spectrum of **2q** (100 MHz, $CDCl_3$)

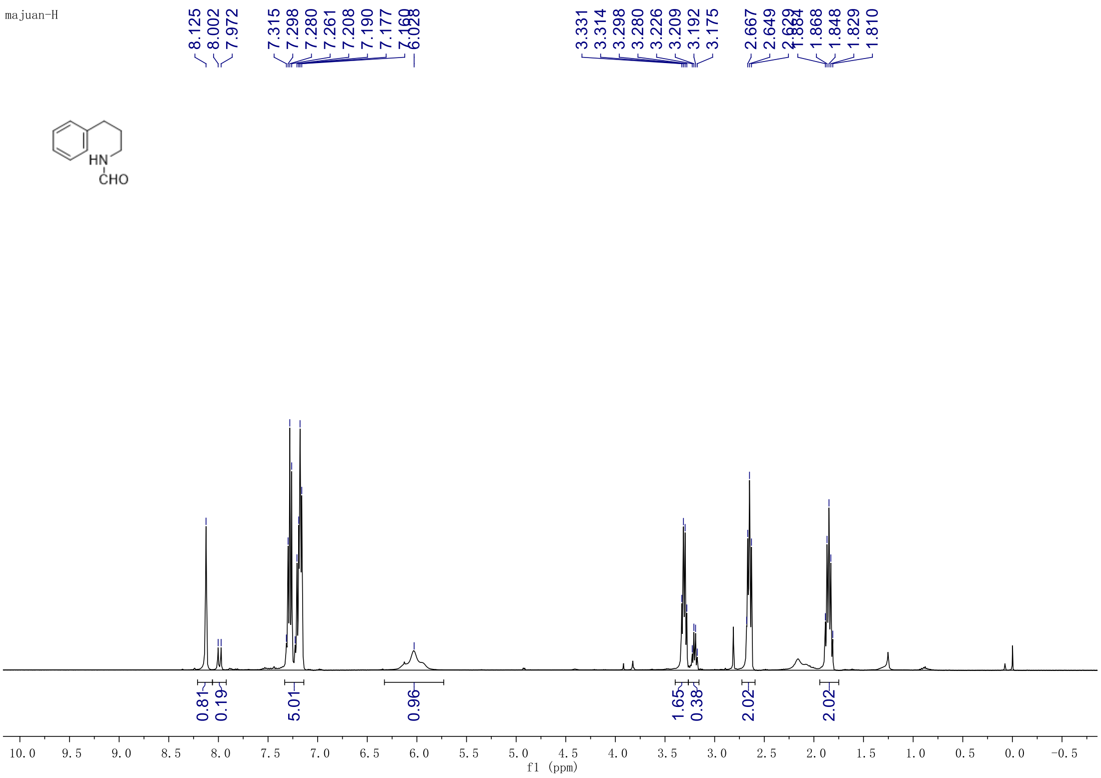

^{1}H NMR spectrum of **2r** (400 MHz, $CDCl_3$)

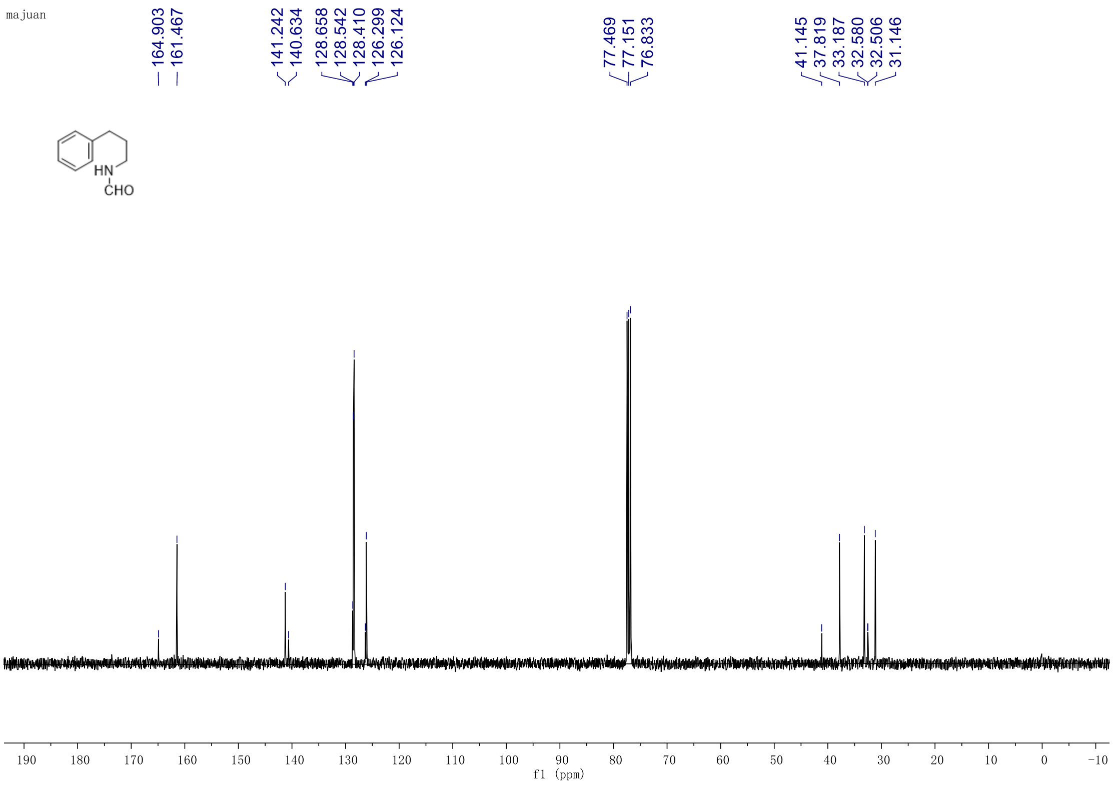

^{13}C NMR spectrum of **2r** (100 MHz, $CDCl_3$)

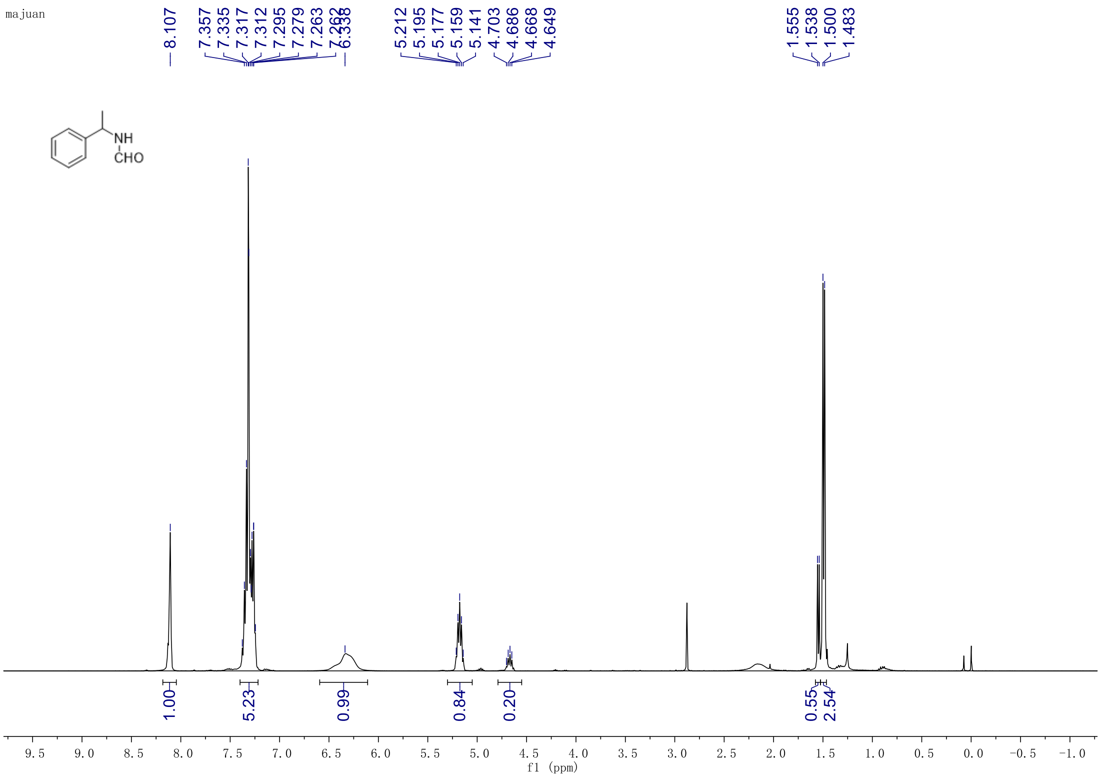

^{1}H NMR spectrum of **2s** (400 MHz, $CDCl_3$)

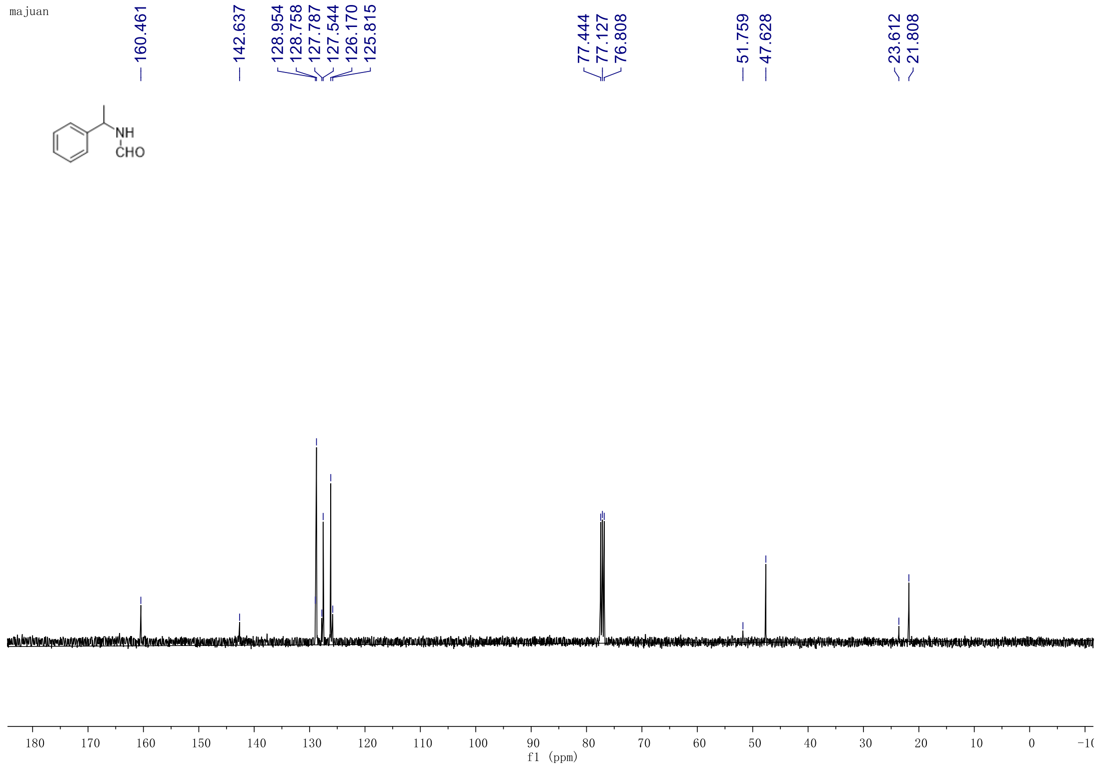

^{13}C NMR spectrum of **2s** (100 MHz, $CDCl_3$)

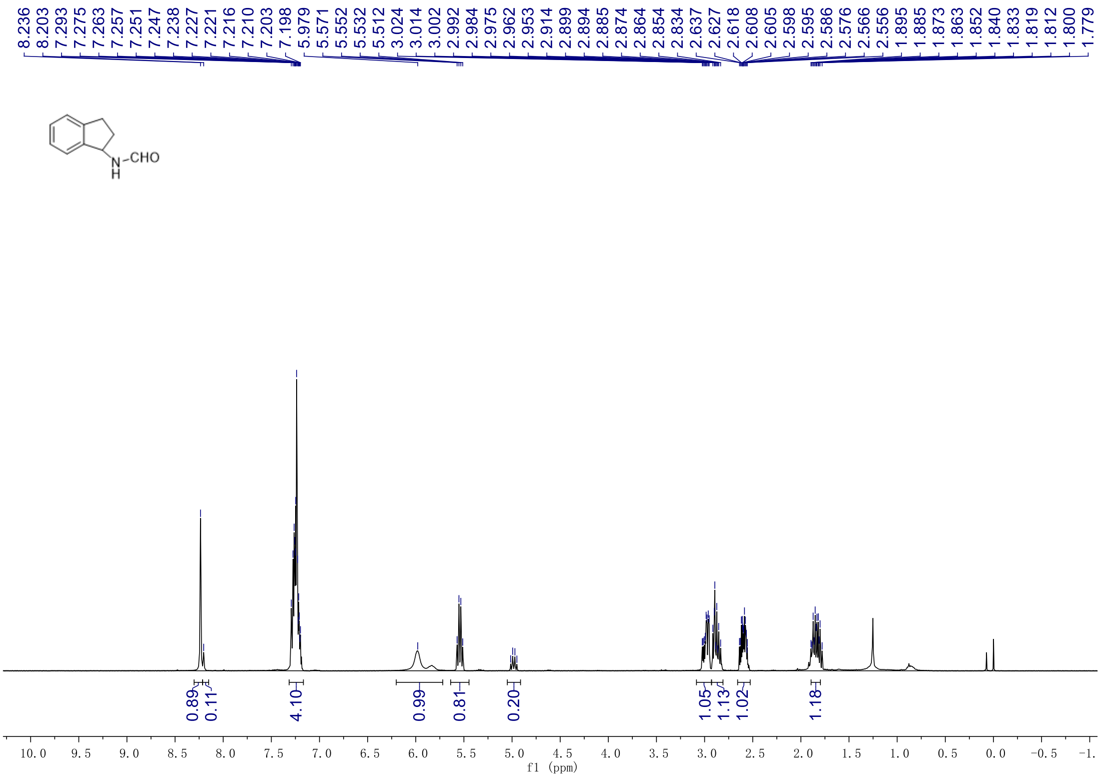

^{1}H NMR spectrum of **2t** (400 MHz, $CDCl_3$)

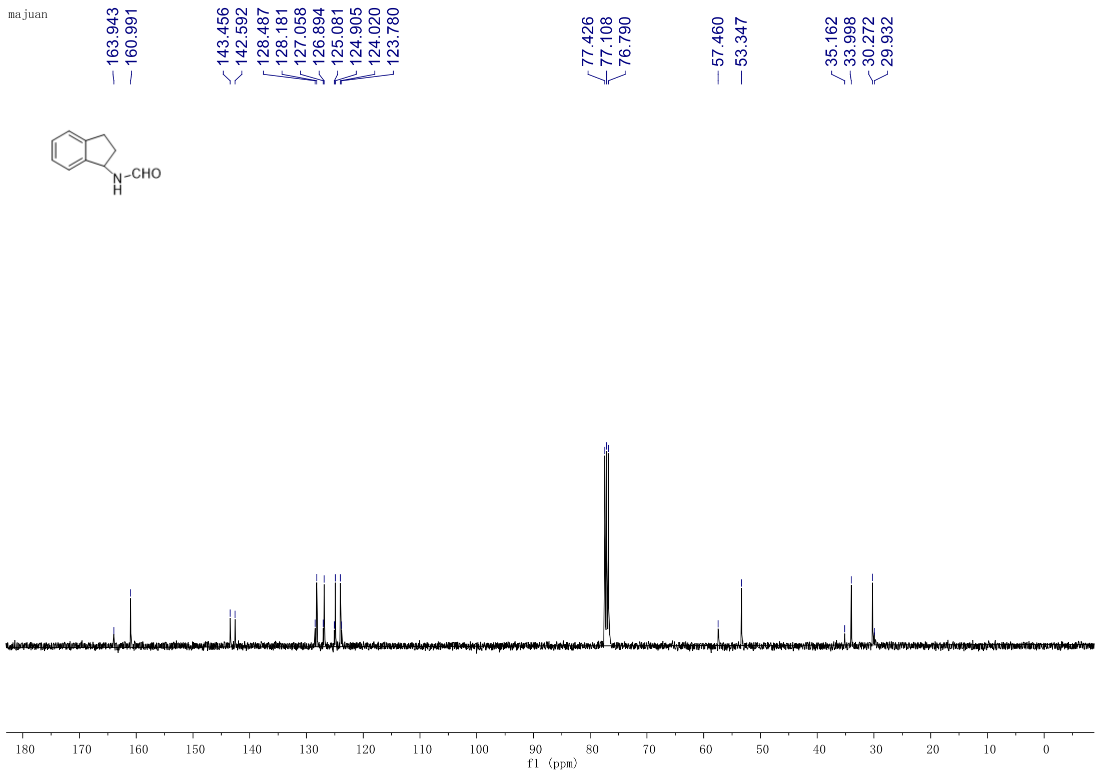

^{13}C NMR spectrum of **2t** (100 MHz, $CDCl_3$)

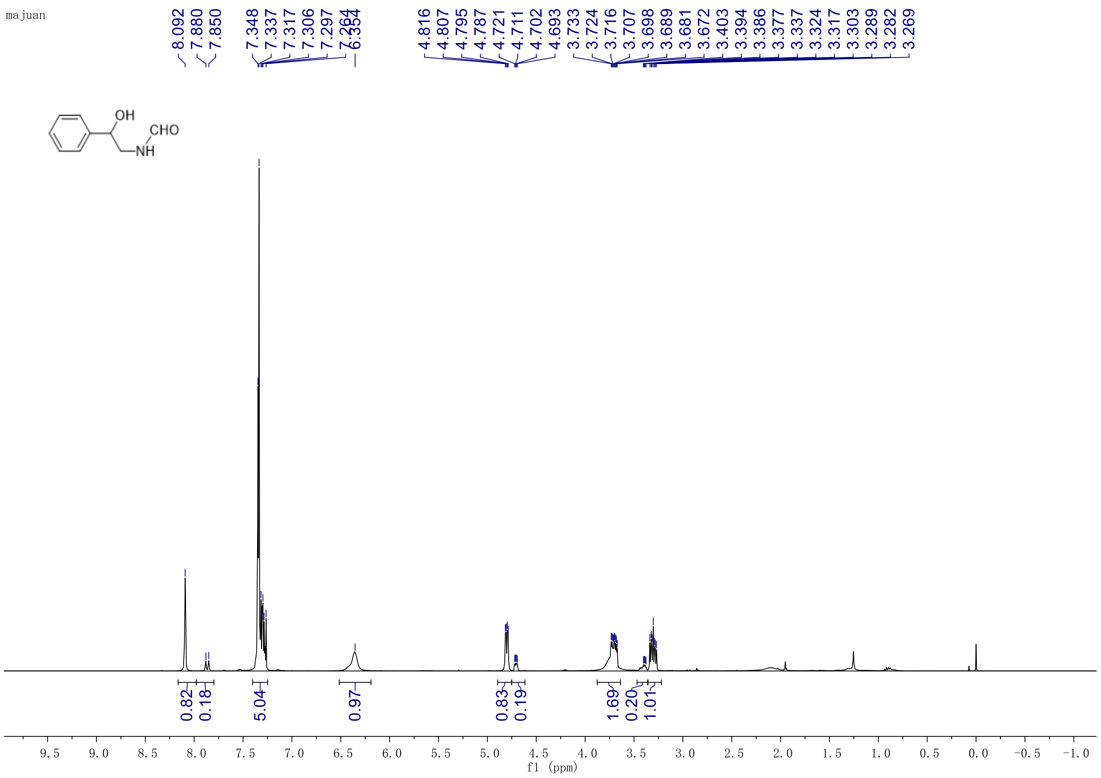

^{1}H NMR spectrum of **2u** (400 MHz, $CDCl_3$)

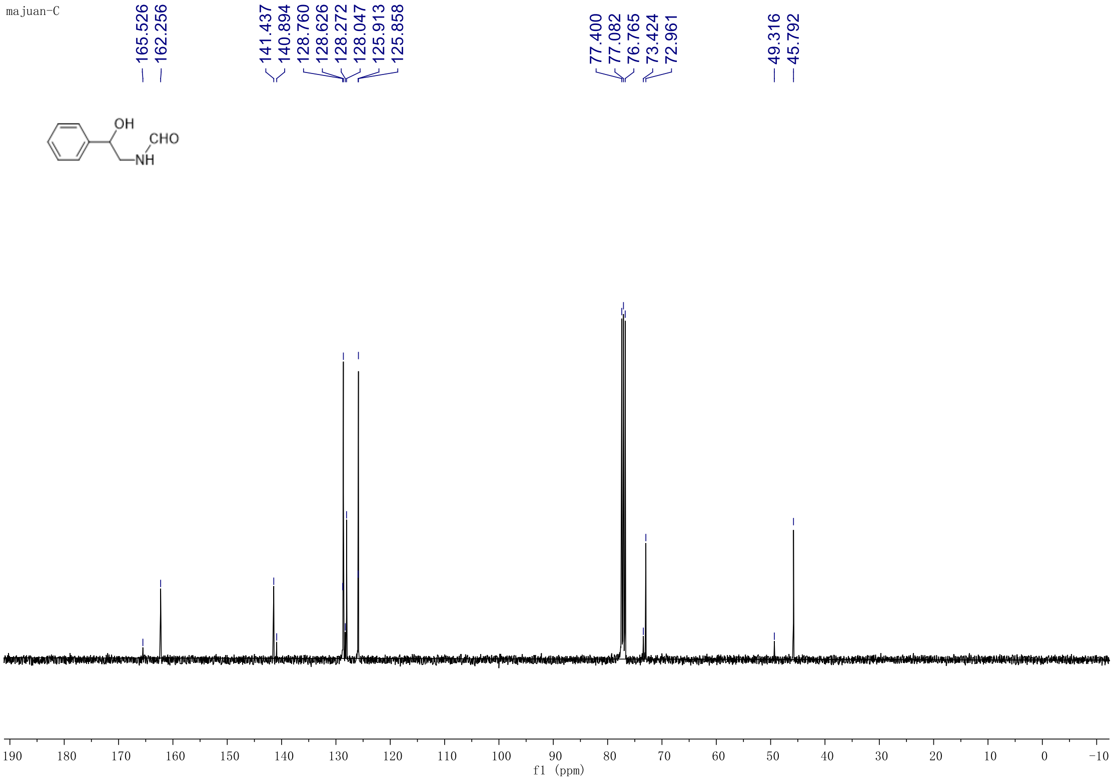

^{13}C NMR spectrum of **2u** (100 MHz, $CDCl_3$)

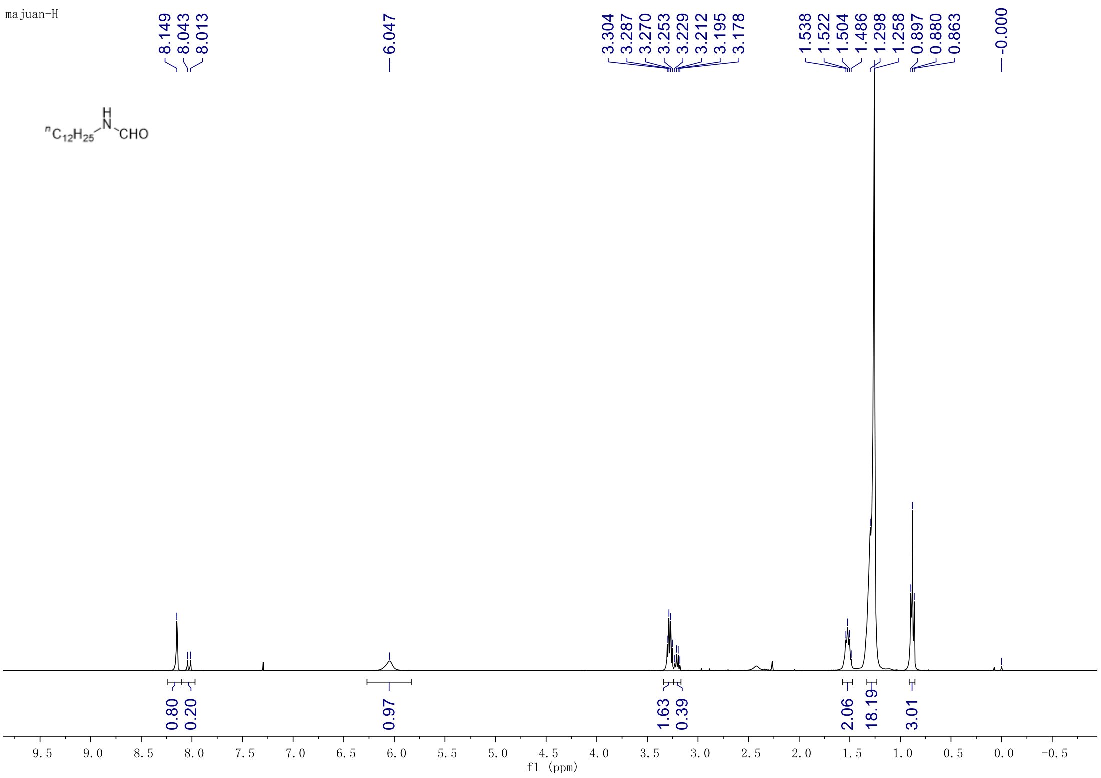

^{1}H NMR spectrum of **2v** (400 MHz, $CDCl_3$)

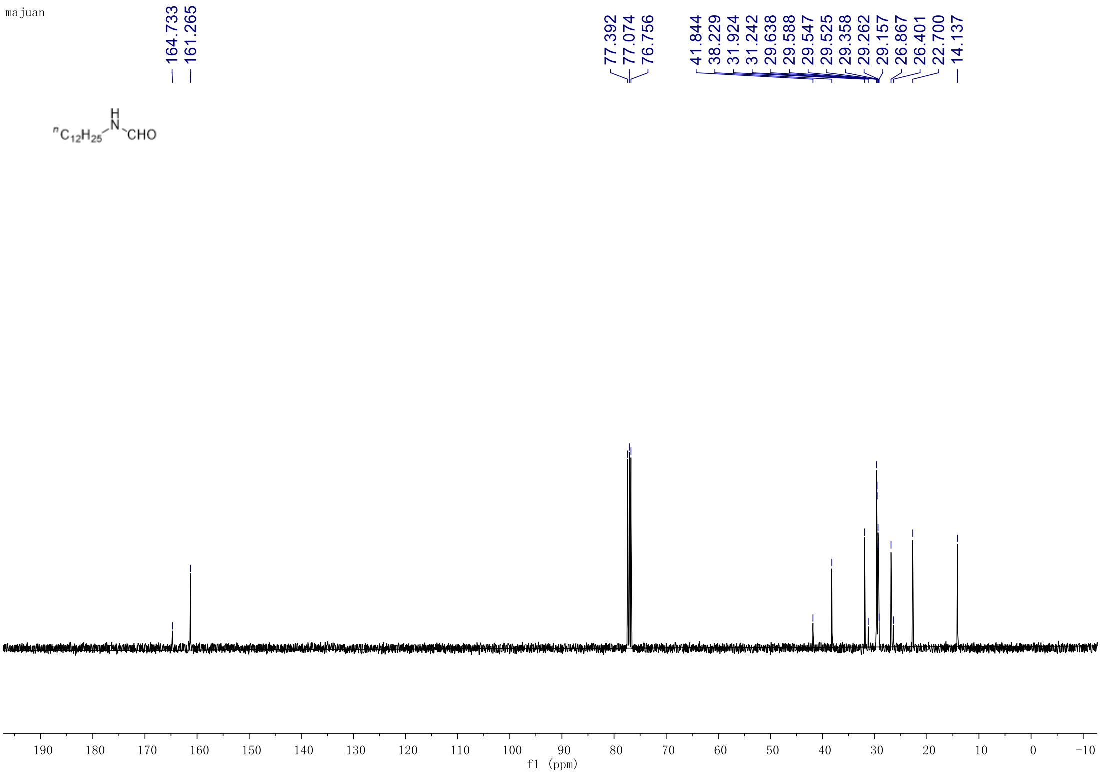

^{13}C NMR spectrum of **2v** (100 MHz, $CDCl_3$)

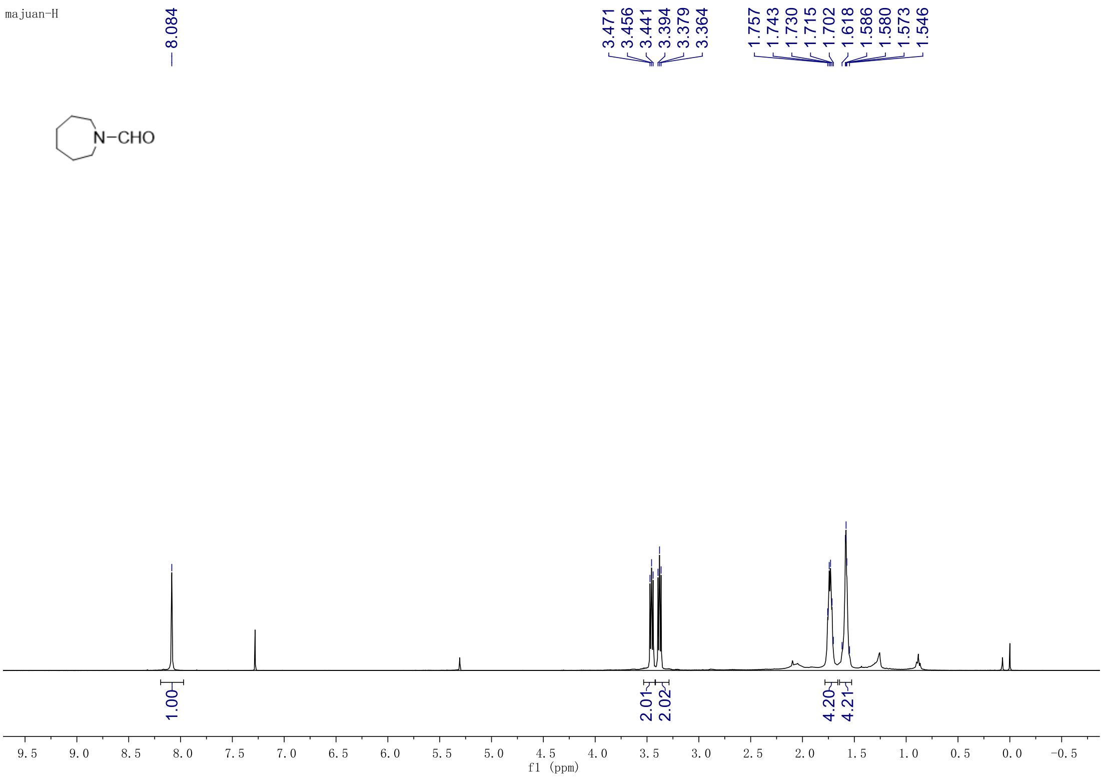

^{1}H NMR spectrum of **2w** (400 MHz, $CDCl_3$)

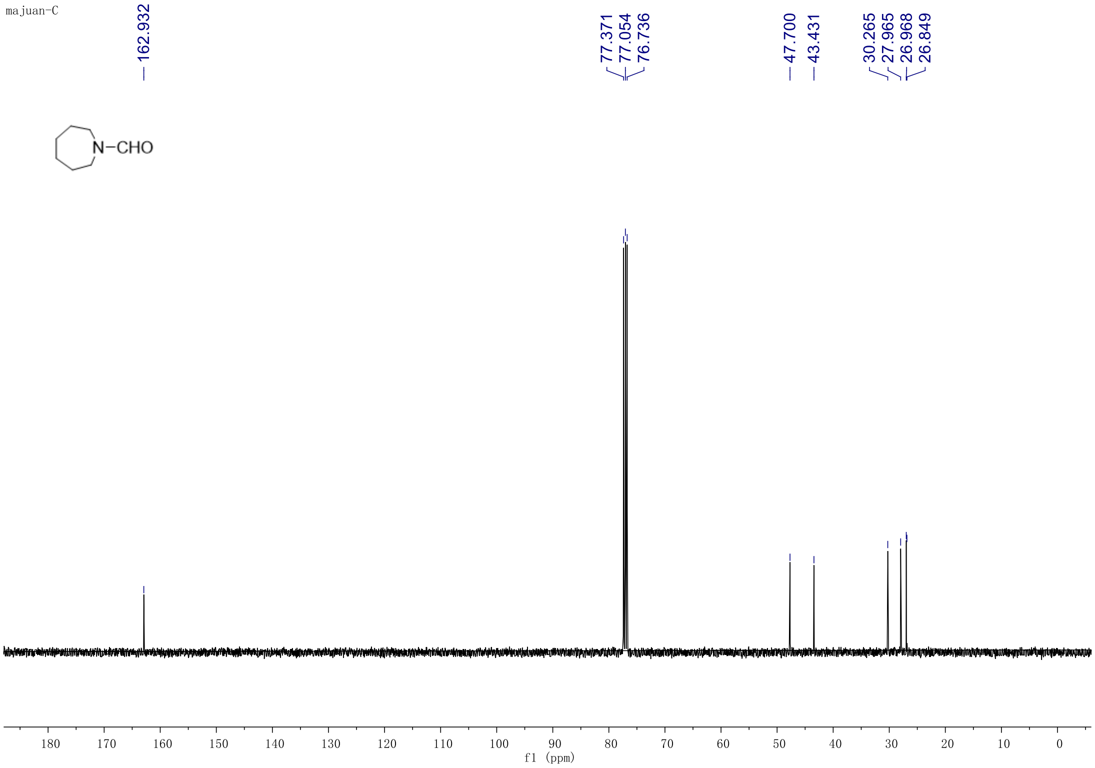

^{13}C NMR spectrum of **2w** (100 MHz, $CDCl_3$)

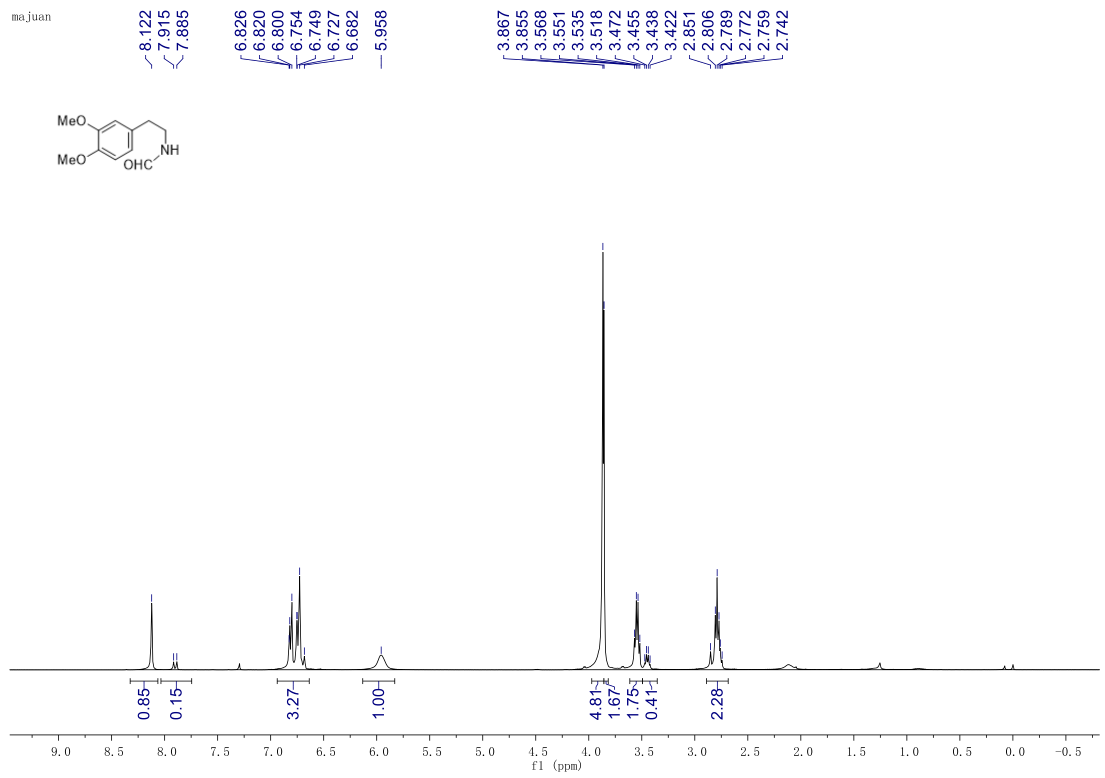

^{1}H NMR spectrum of **2x** (400 MHz, $CDCl_3$)

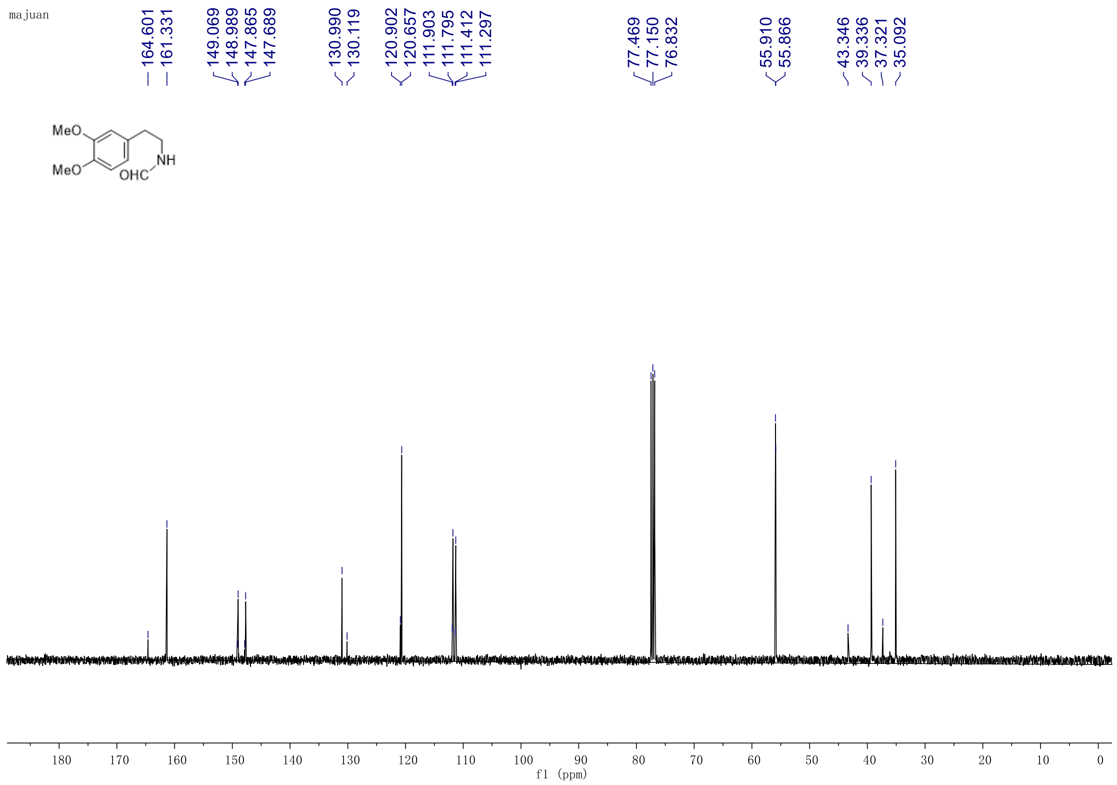

^{13}C NMR spectrum of **2x** (100 MHz, $CDCl_3$)

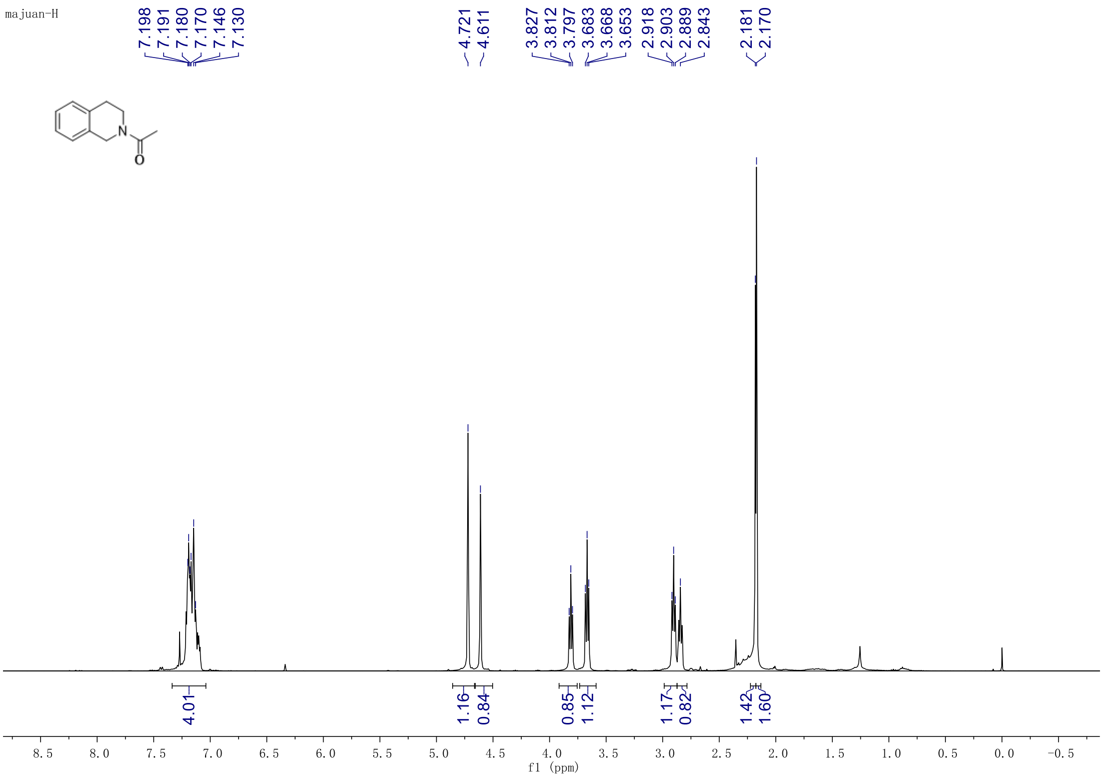

^{1}H NMR spectrum of **2y** (400 MHz, $CDCl_3$)

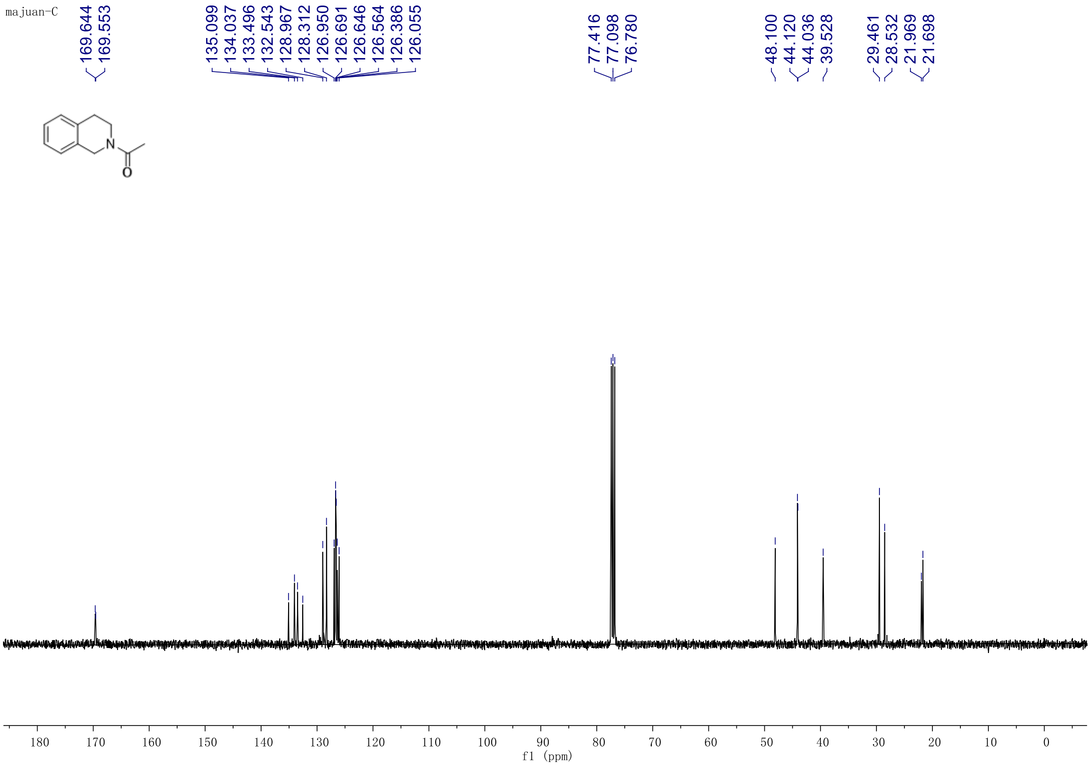

^{13}C NMR spectrum of **2y** (100 MHz, $CDCl_3$)

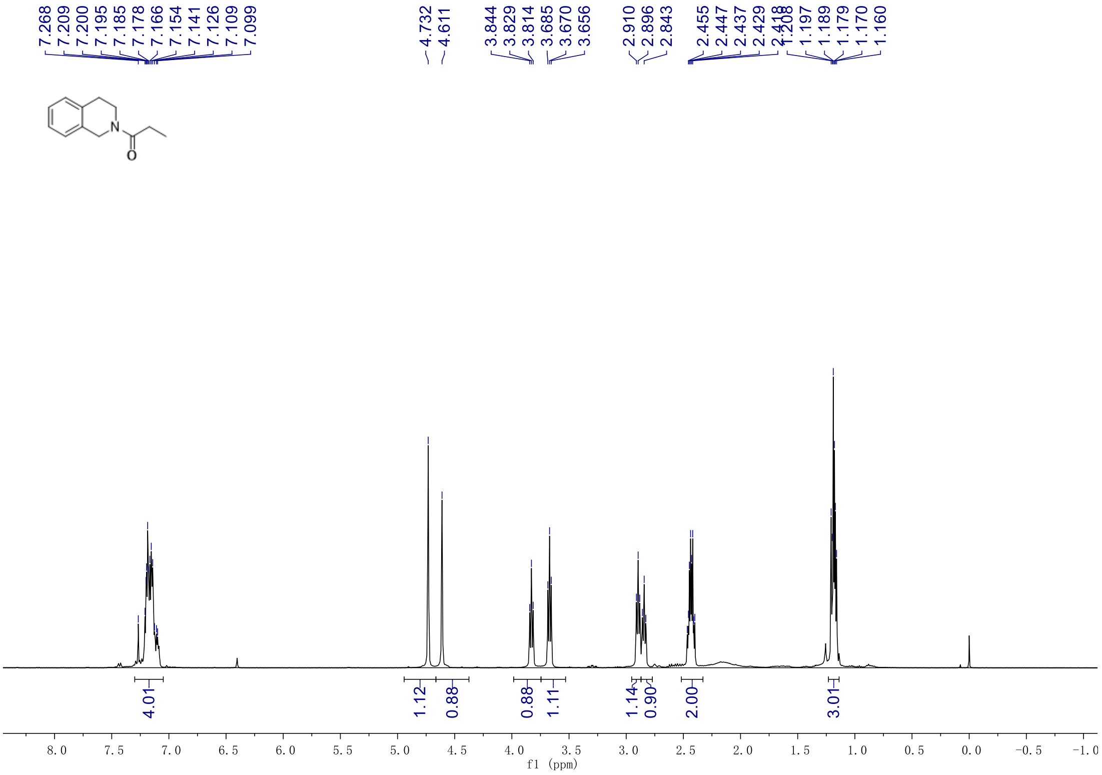

^{1}H NMR spectrum of **2z** (400 MHz, $CDCl_3$)

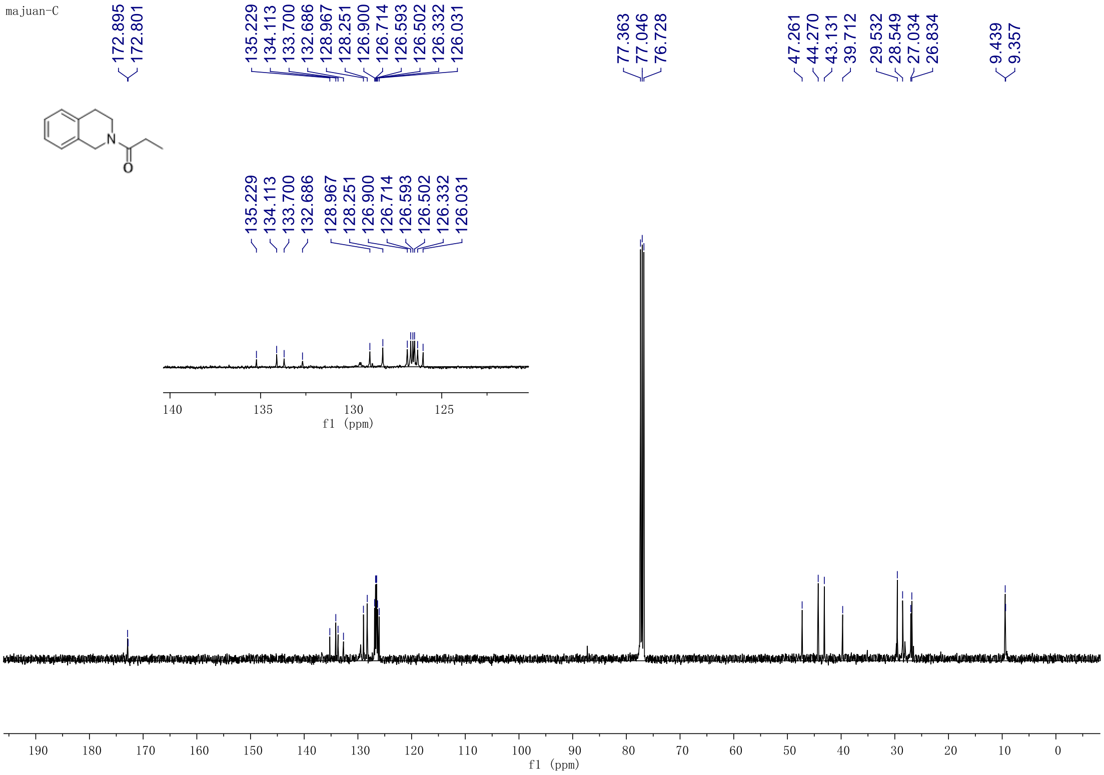

^{13}C NMR spectrum of **2z** (100 MHz, $CDCl_3$)

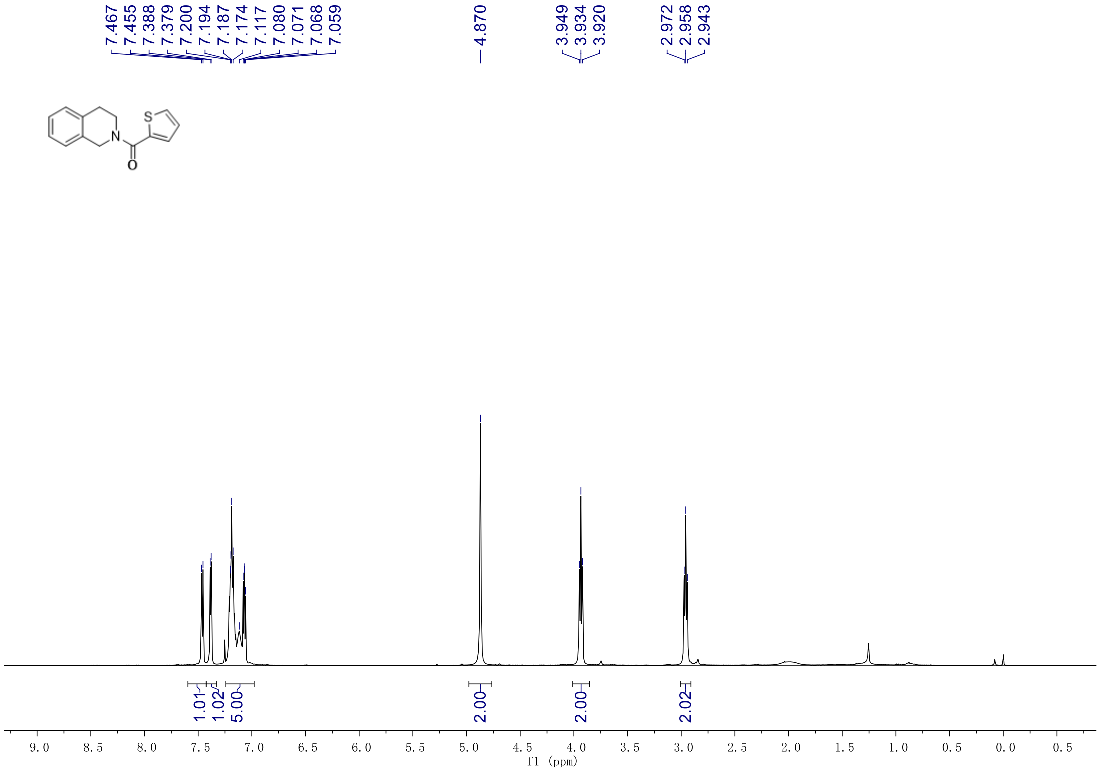

^{1}H NMR spectrum of **2za** (400 MHz, $CDCl_3$)

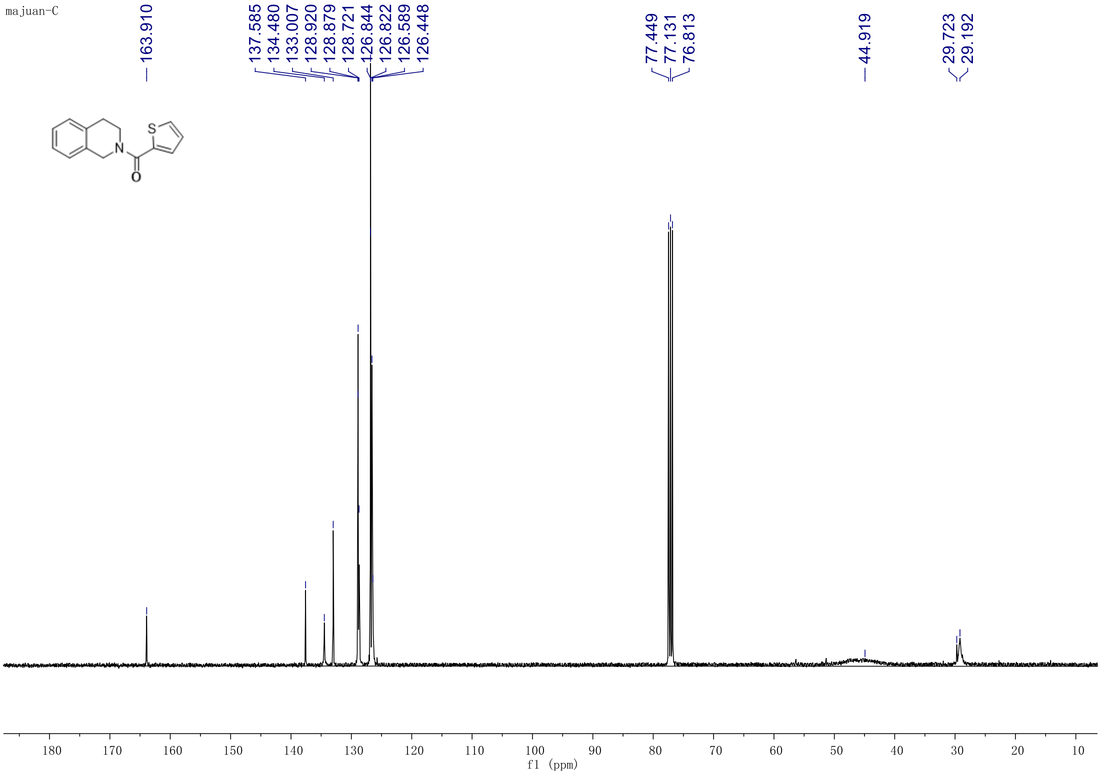

^{13}C NMR spectrum of **2za** (100 MHz, $CDCl_3$)

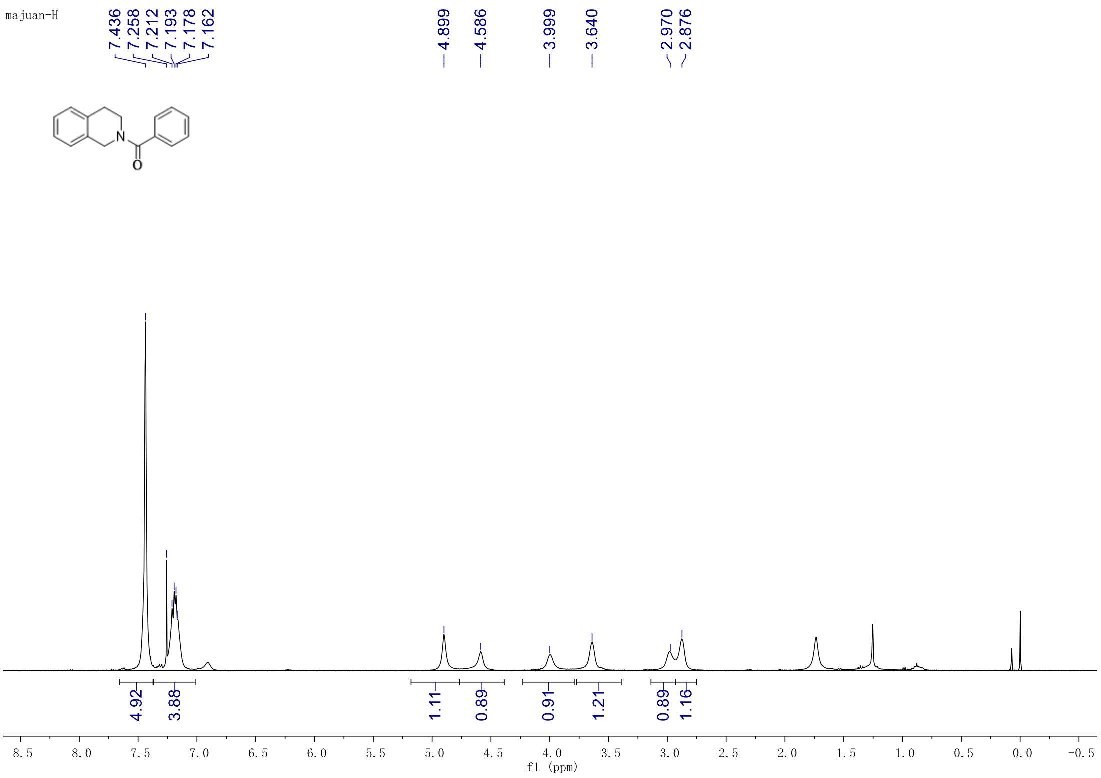

^{1}H NMR spectrum of **2zb** (400 MHz, $CDCl_3$)

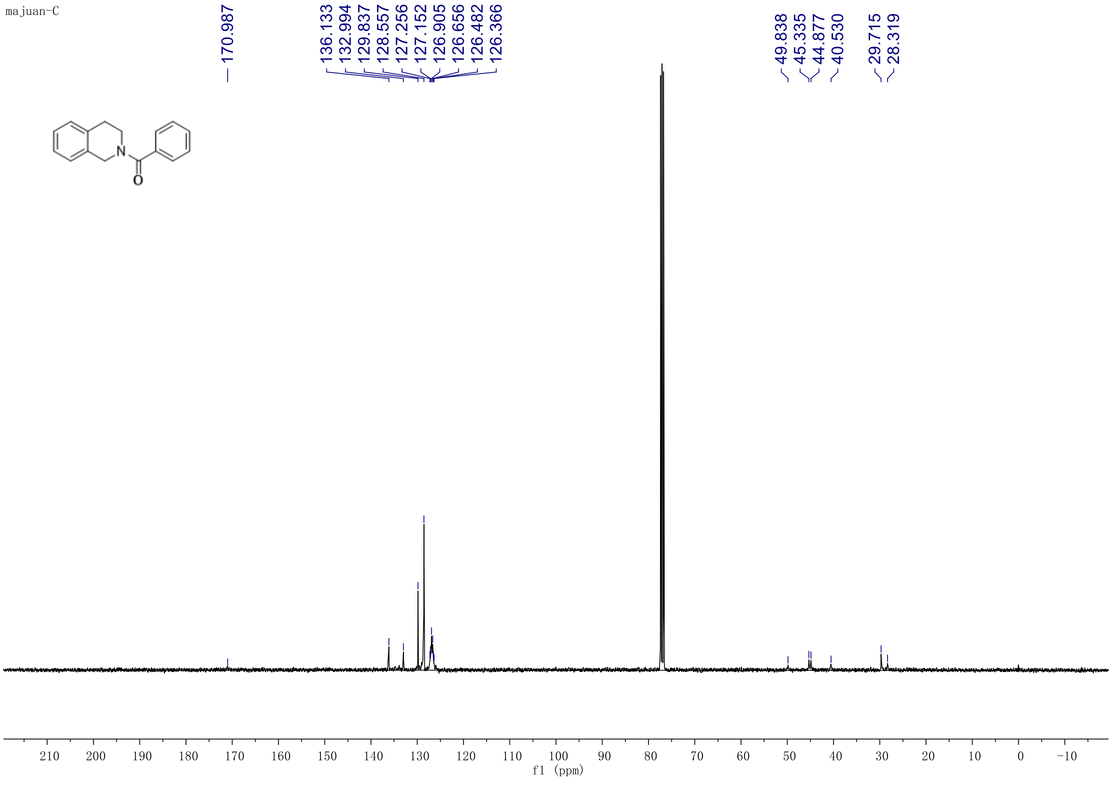

^{13}C NMR spectrum of **2zb** (100 MHz, $CDCl_3$)

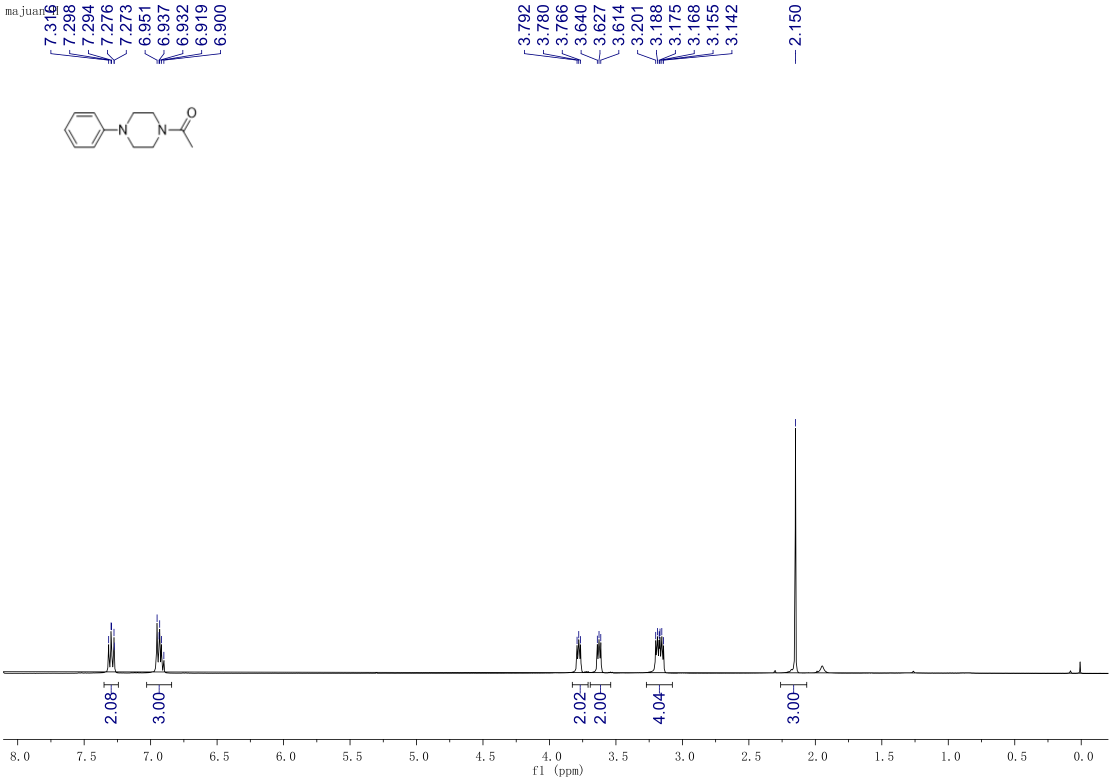

^{1}H NMR spectrum of **2zc** (400 MHz, $CDCl_3$)

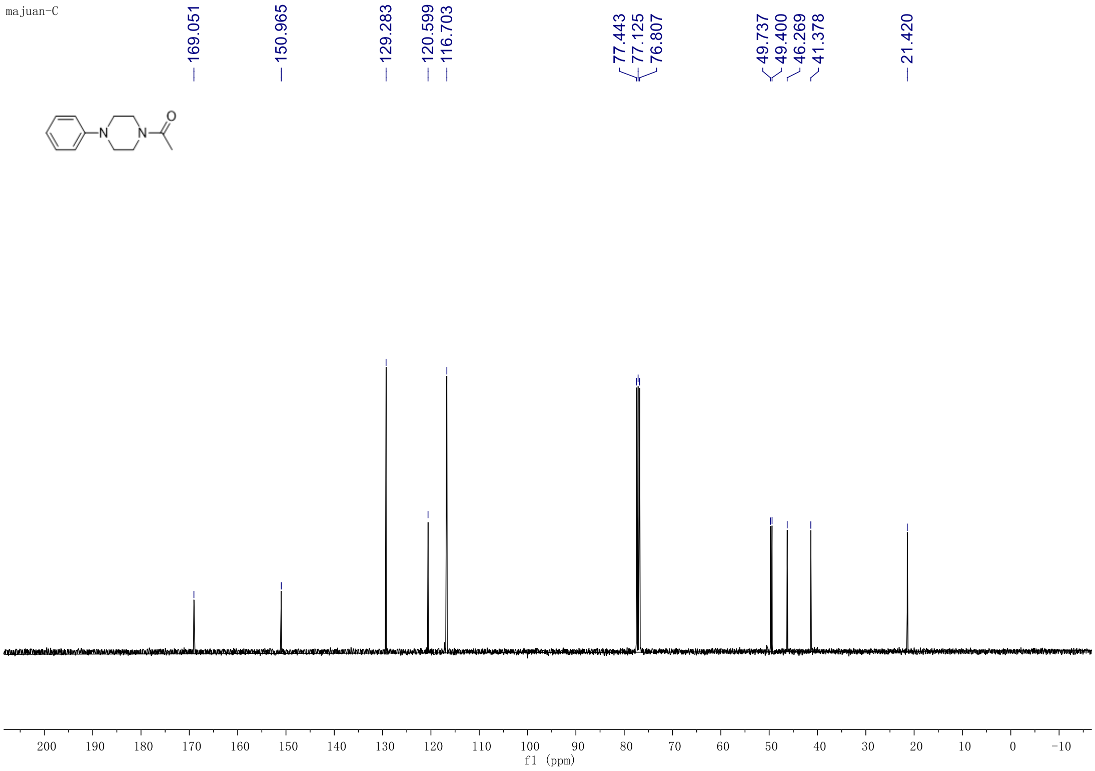

^{13}C NMR spectrum of **2zc** (100 MHz, $CDCl_3$)

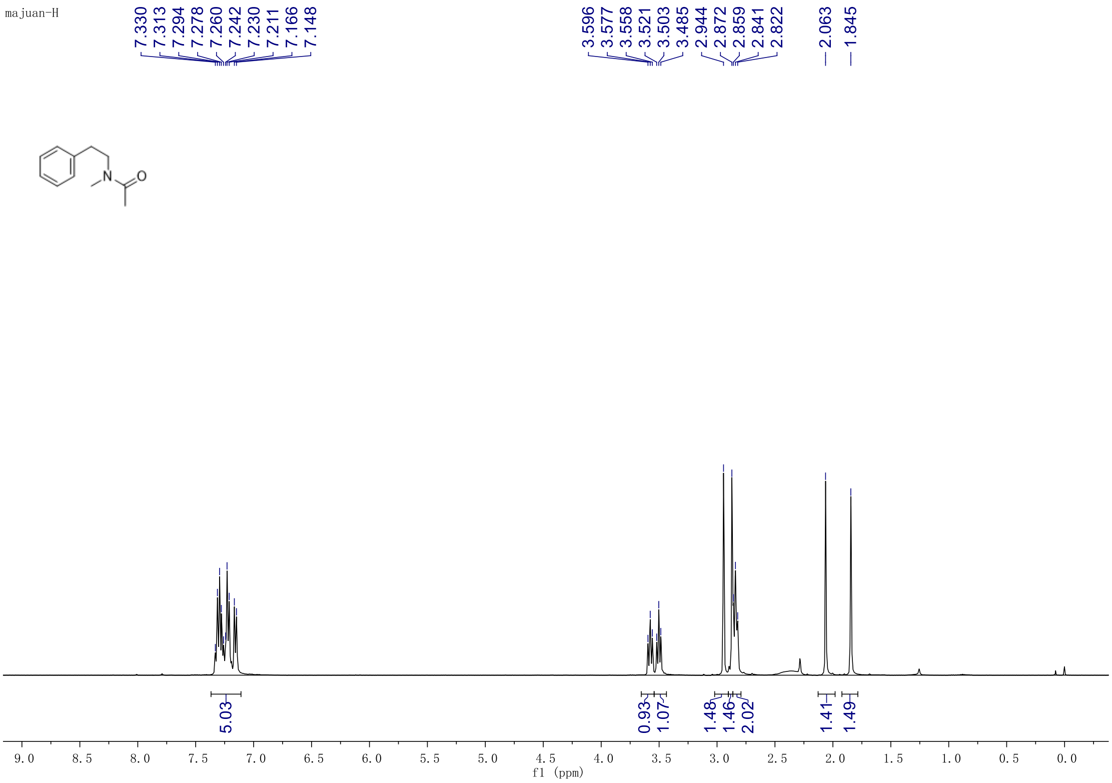

^{1}H NMR spectrum of **2zd** (400 MHz, $CDCl_3$)

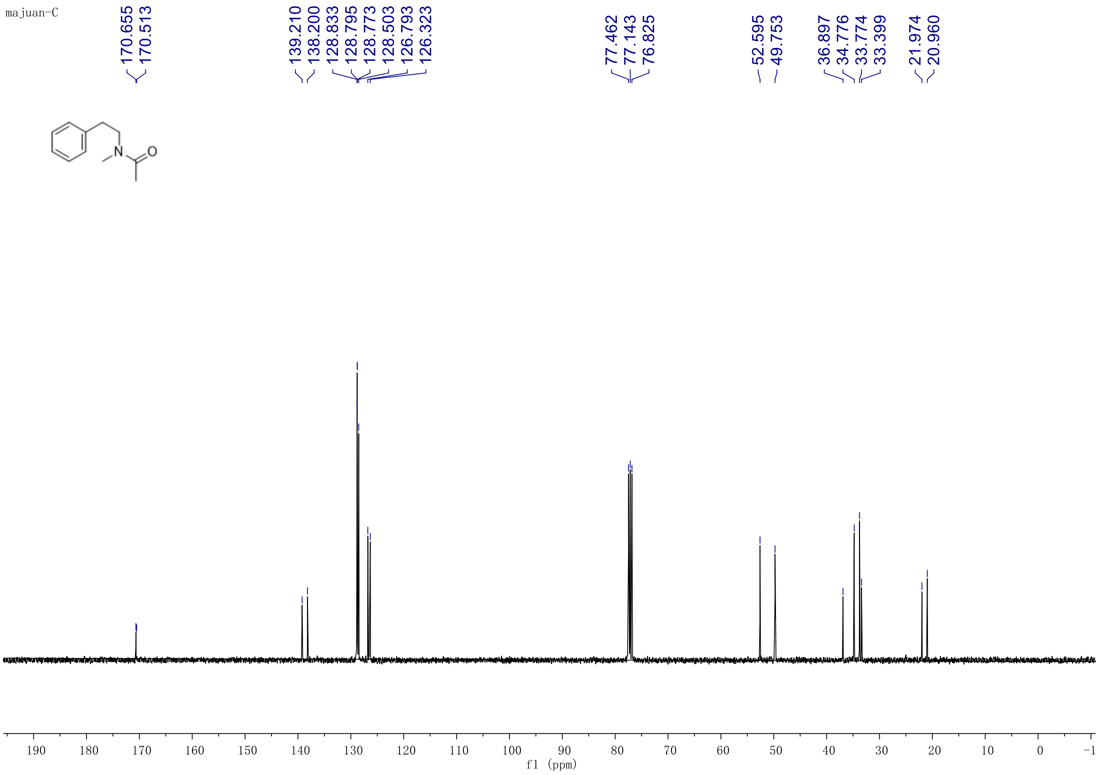

^{13}C NMR spectrum of **2zd** (100 MHz, $CDCl_3$)

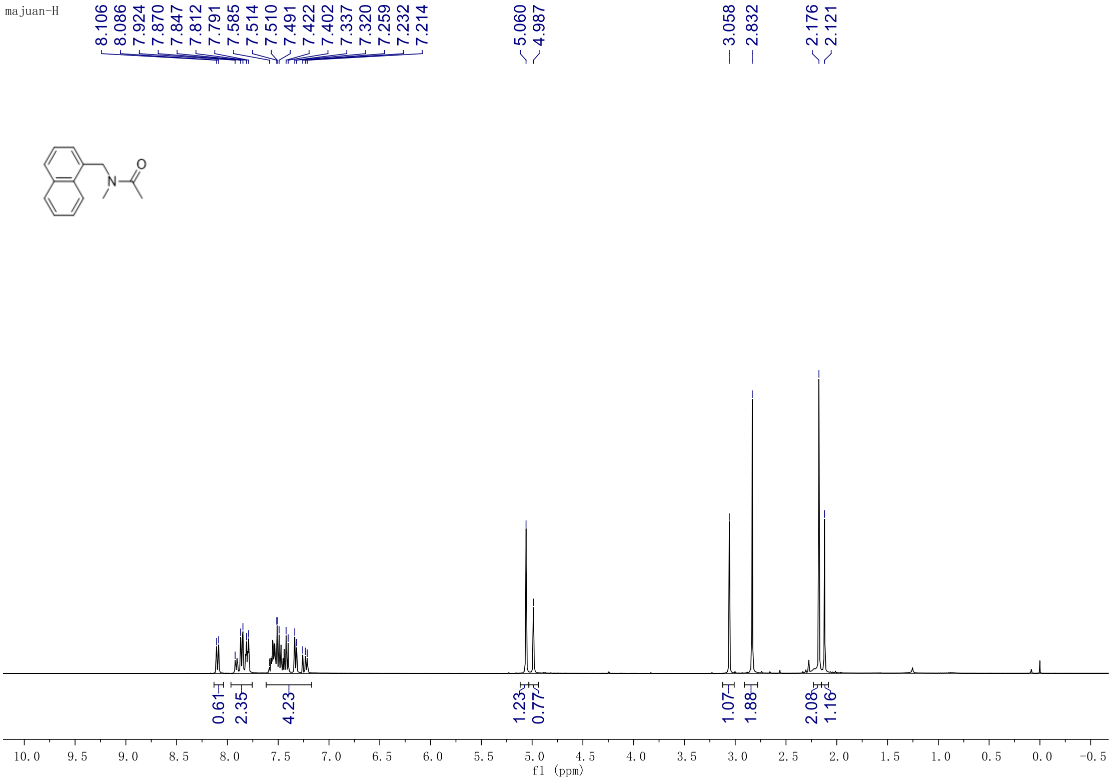

^{1}H NMR spectrum of **2ze** (400 MHz, $CDCl_3$)

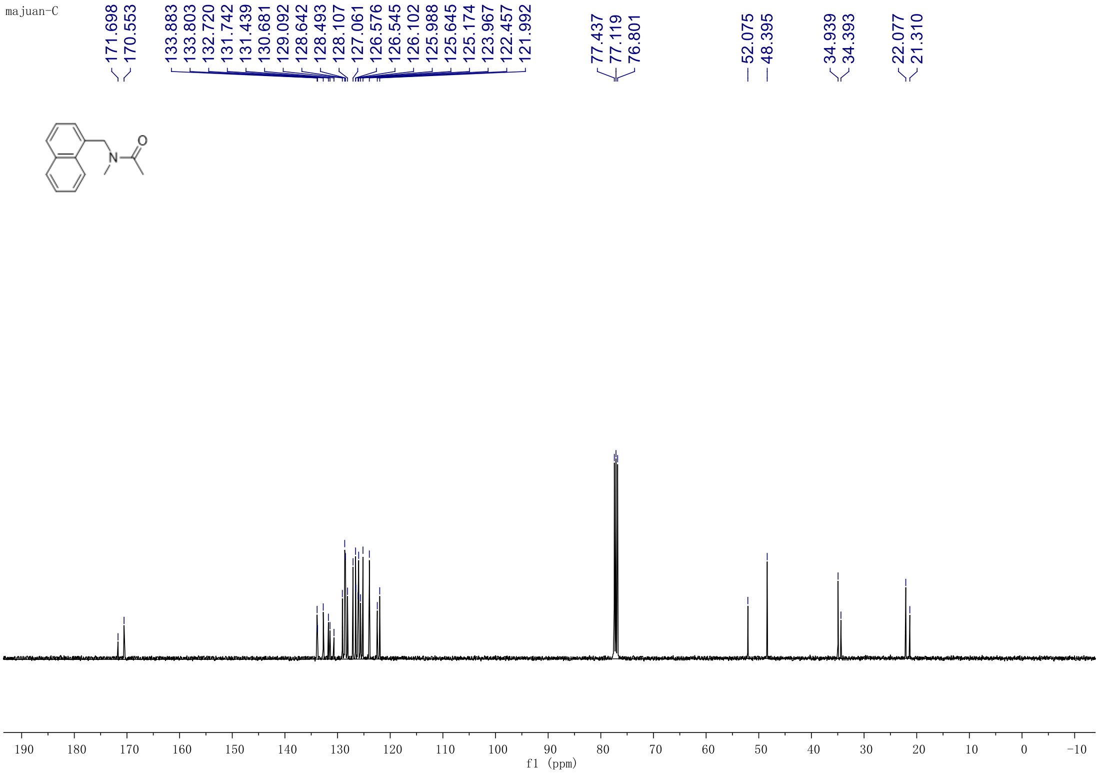

^{13}C NMR spectrum of **2ze** (100 MHz, $CDCl_3$)

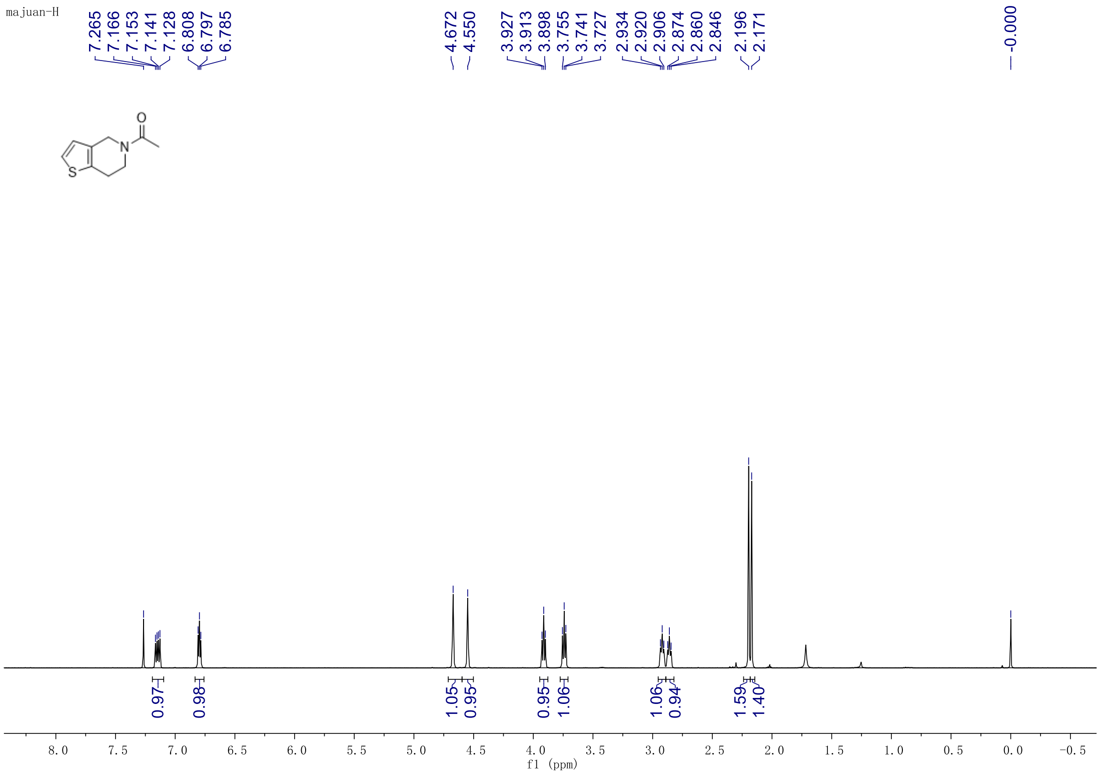

^{1}H NMR spectrum of **2zf** (400 MHz, $CDCl_3$)

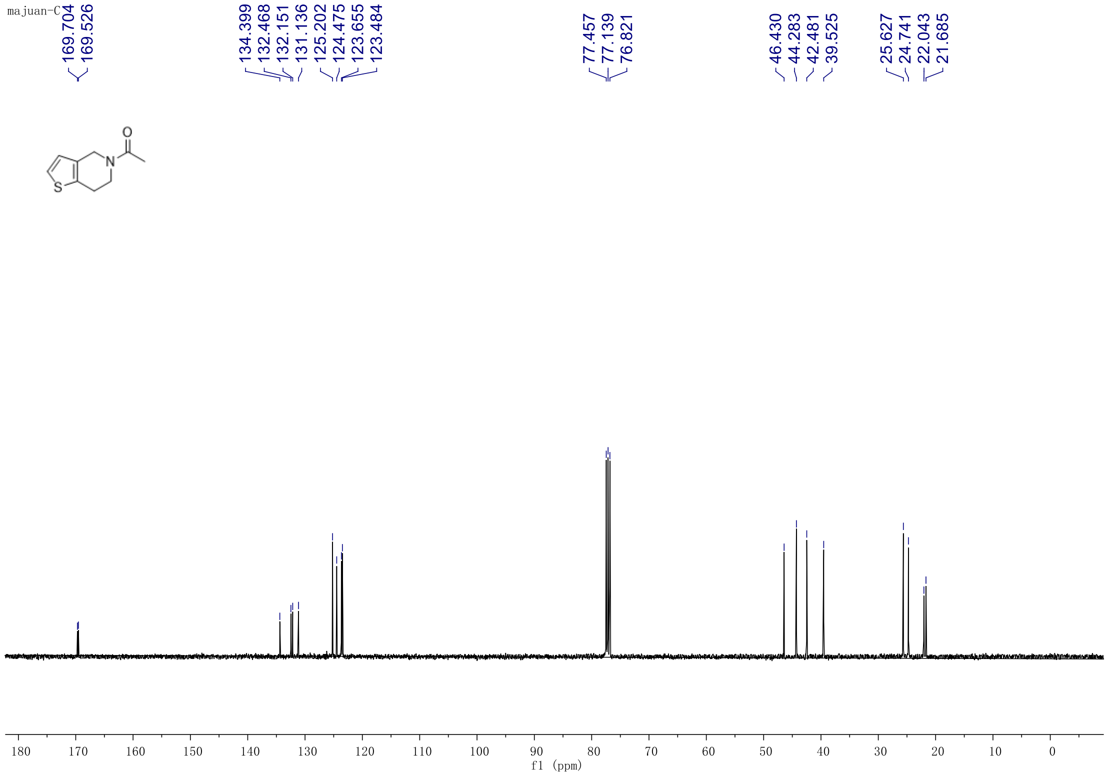

^{13}C NMR spectrum of **2zf** (100 MHz, $CDCl_3$)

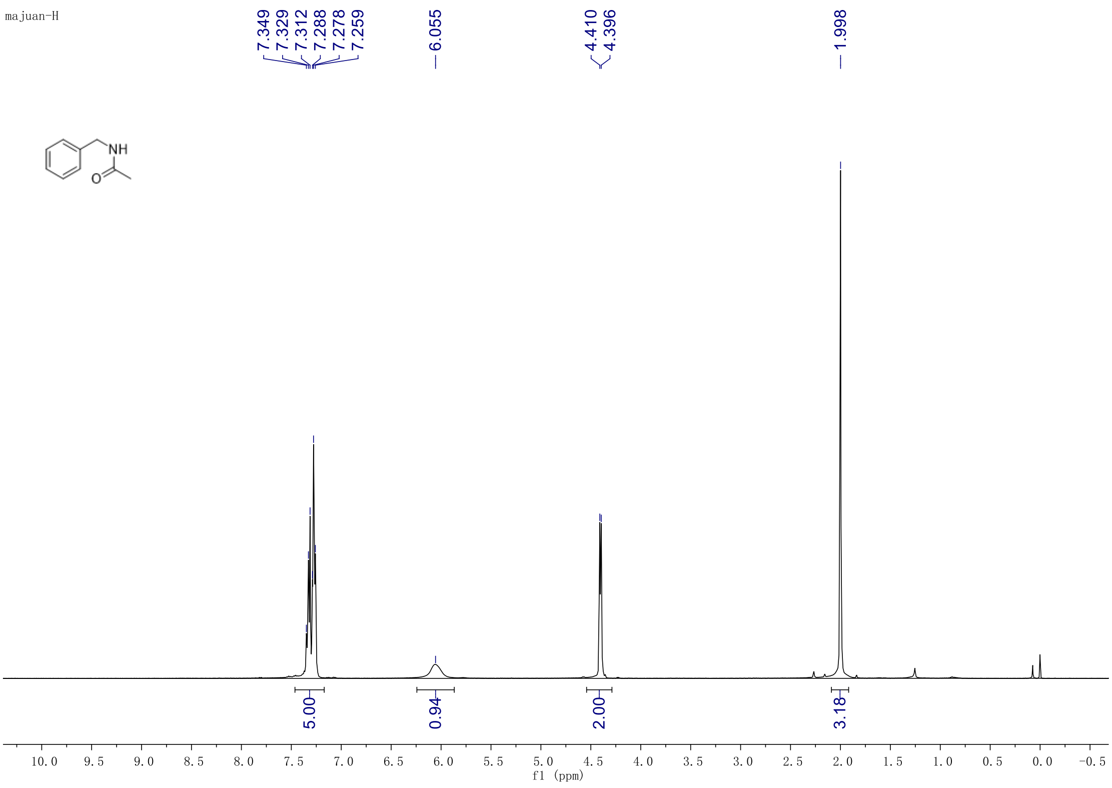

^{1}H NMR spectrum of **2zg** (400 MHz, $CDCl_3$)

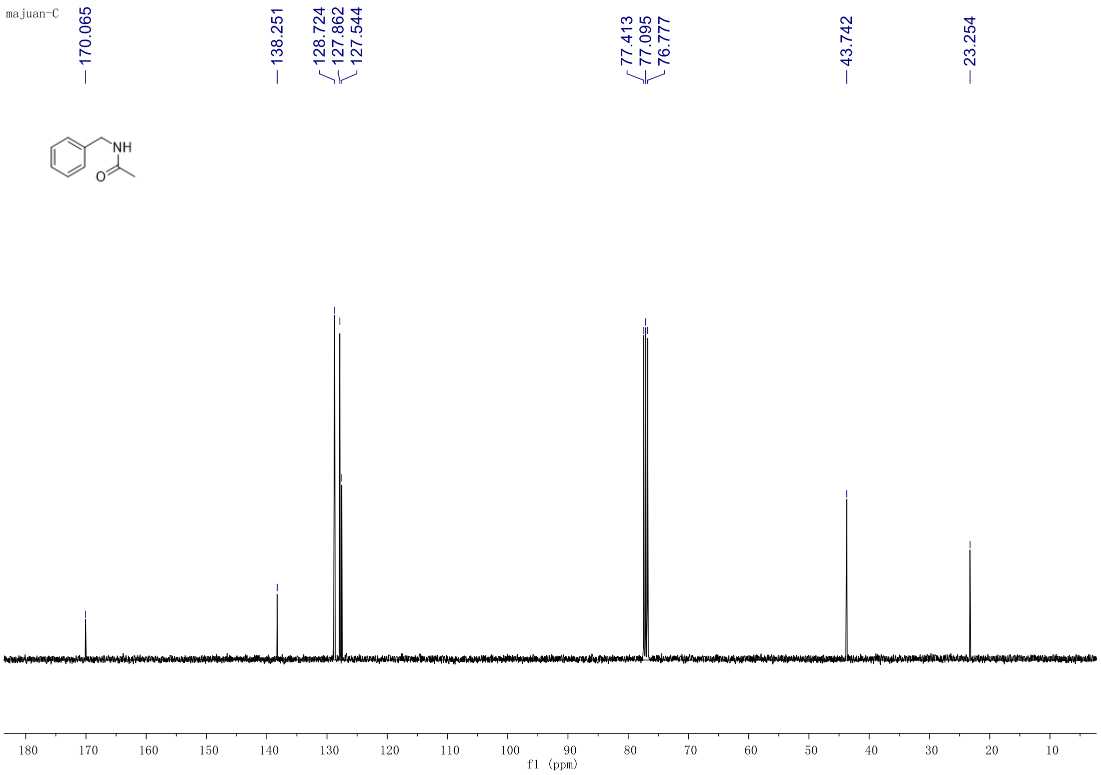

^{13}C NMR spectrum of **2zg** (100 MHz, $CDCl_3$)

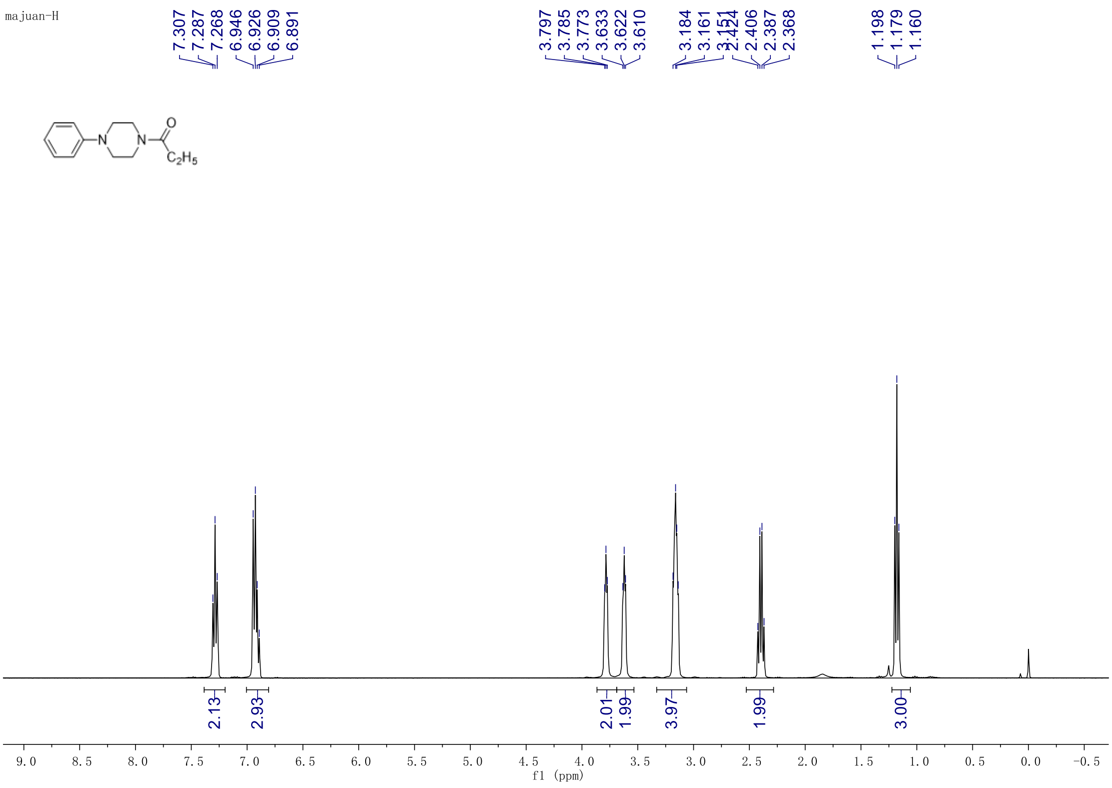

^{1}H NMR spectrum of **2zh** (400 MHz, $CDCl_3$)

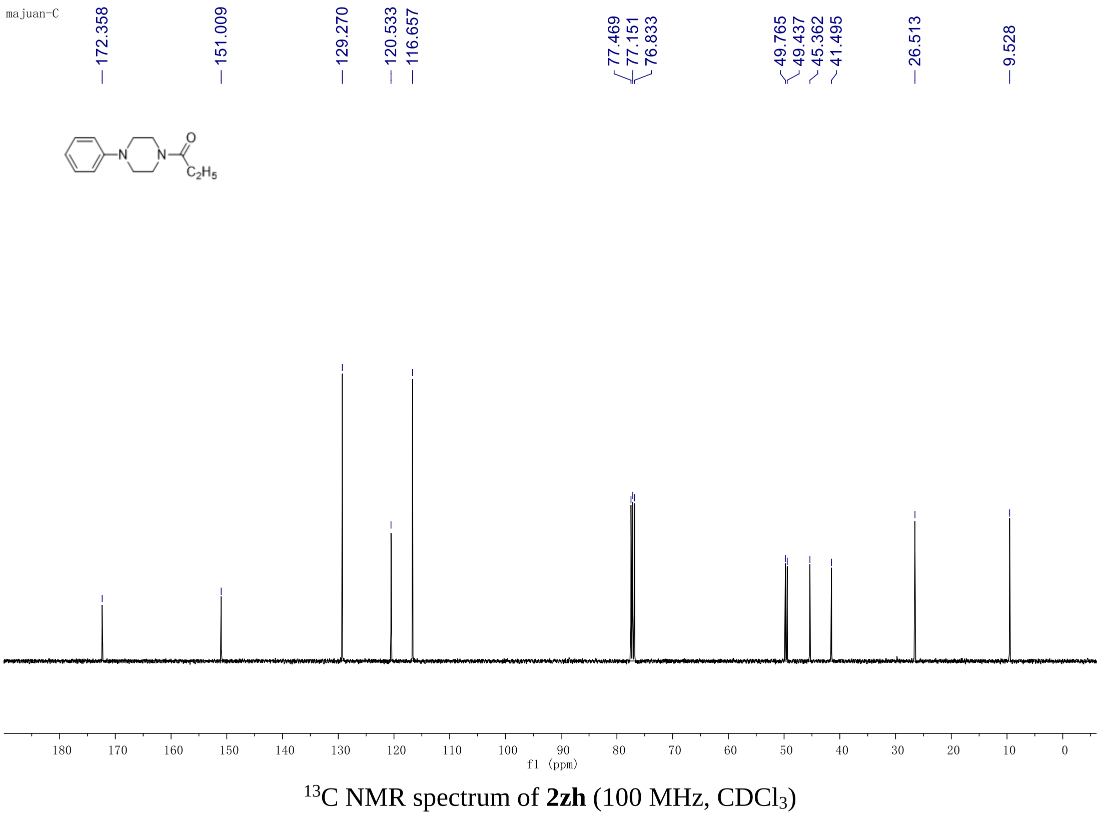

^{13}C NMR spectrum of **2zh** (100 MHz, $CDCl_3$)

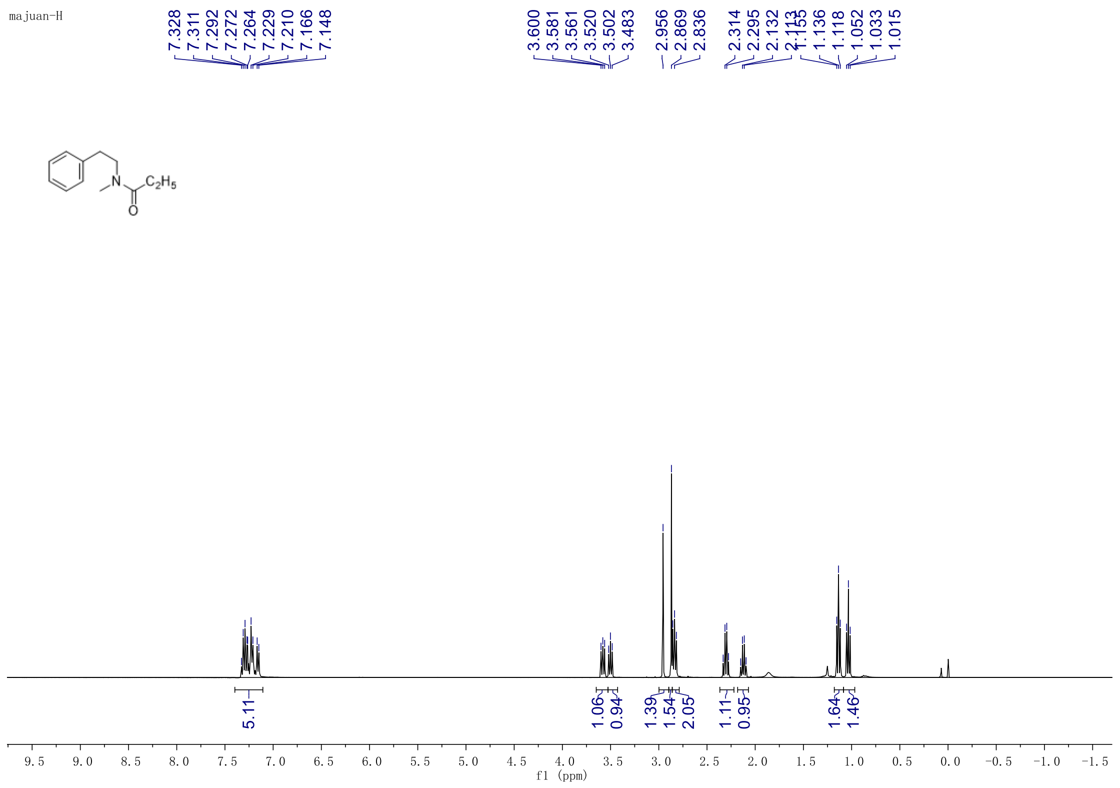

^{1}H NMR spectrum of **2zi** (400 MHz, $CDCl_3$)

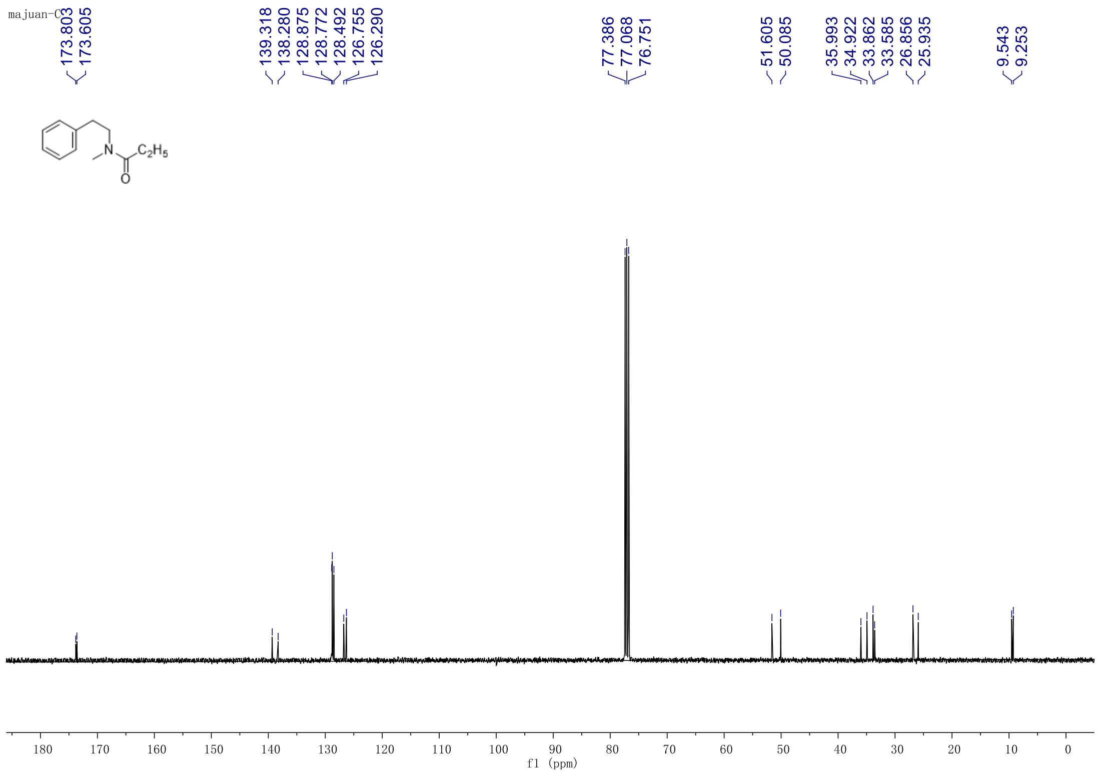

^{13}C NMR spectrum of **2zi** (100 MHz, $CDCl_3$)

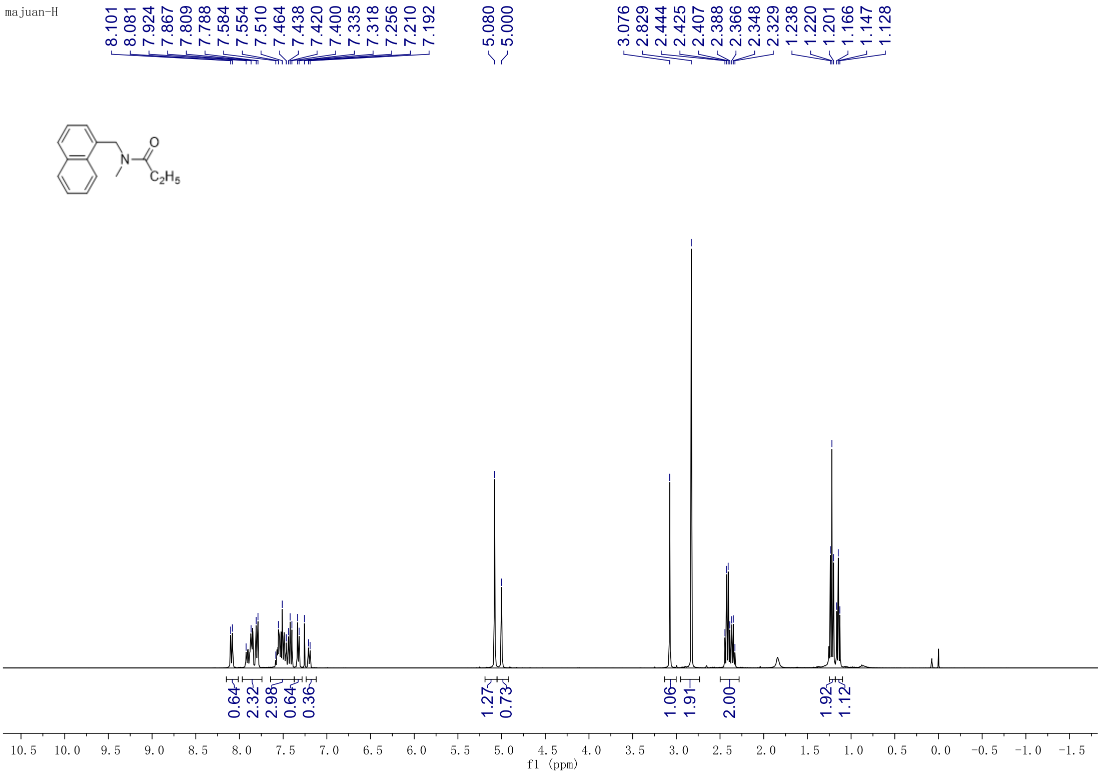

^{1}H NMR spectrum of **2zj** (400 MHz, $CDCl_3$)

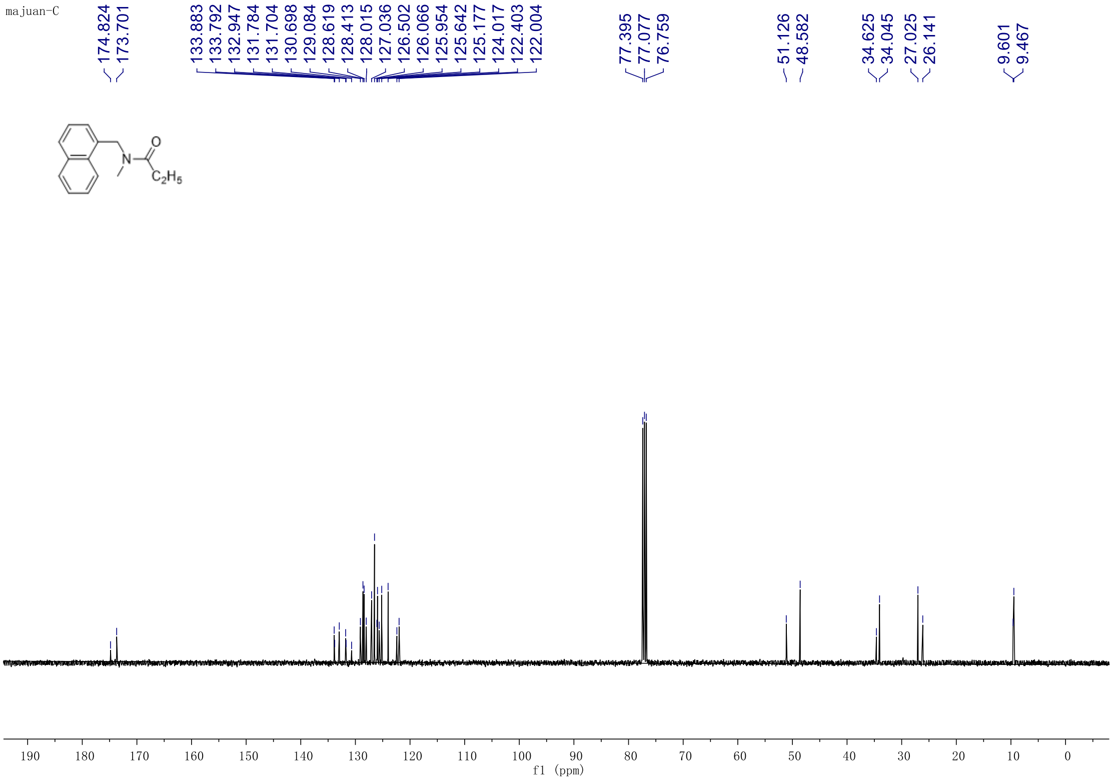

^{13}C NMR spectrum of **2zj** (100 MHz, $CDCl_3$)

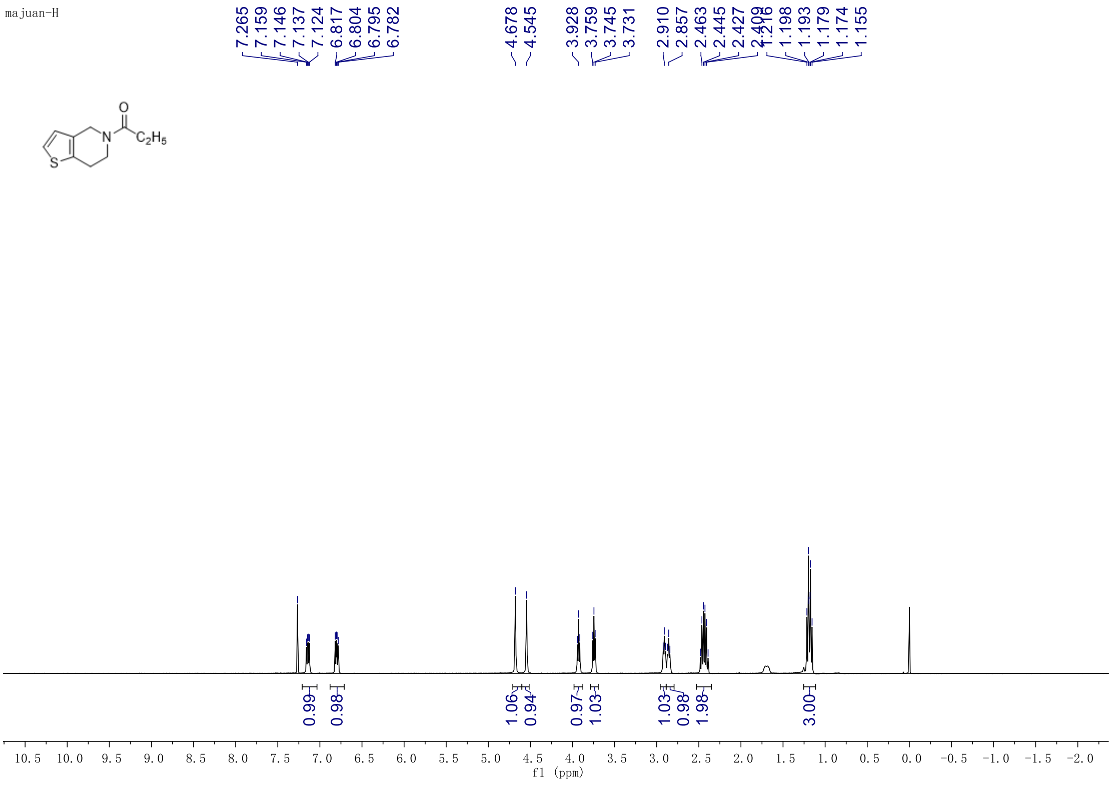

^{1}H NMR spectrum of **2zk** (400 MHz, $CDCl_3$)

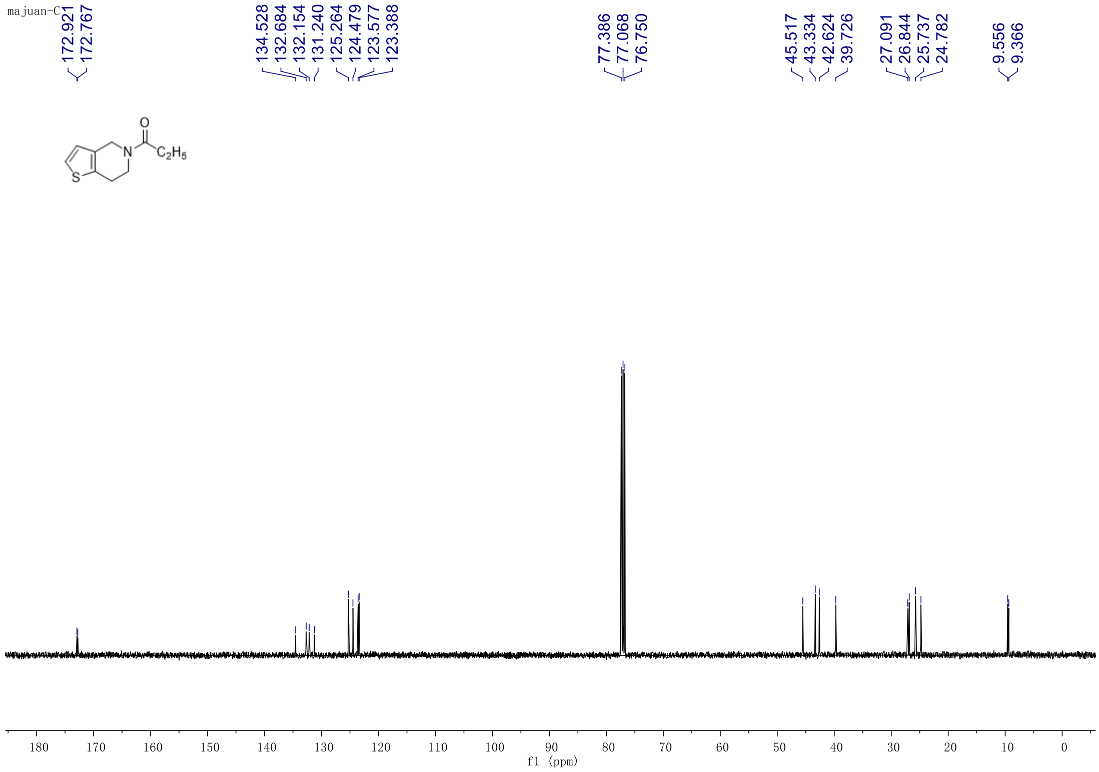

^{13}C NMR spectrum of **2zk** (100 MHz, $CDCl_3$)

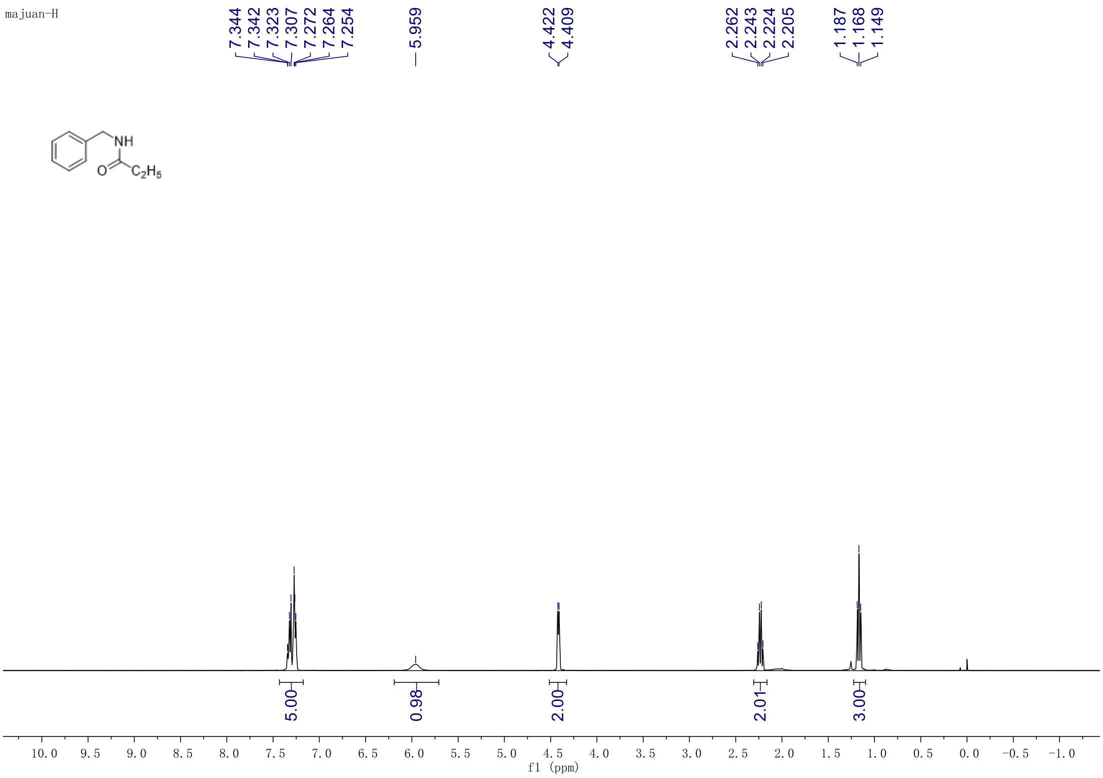

^{1}H NMR spectrum of **2zl** (400 MHz, $CDCl_3$)

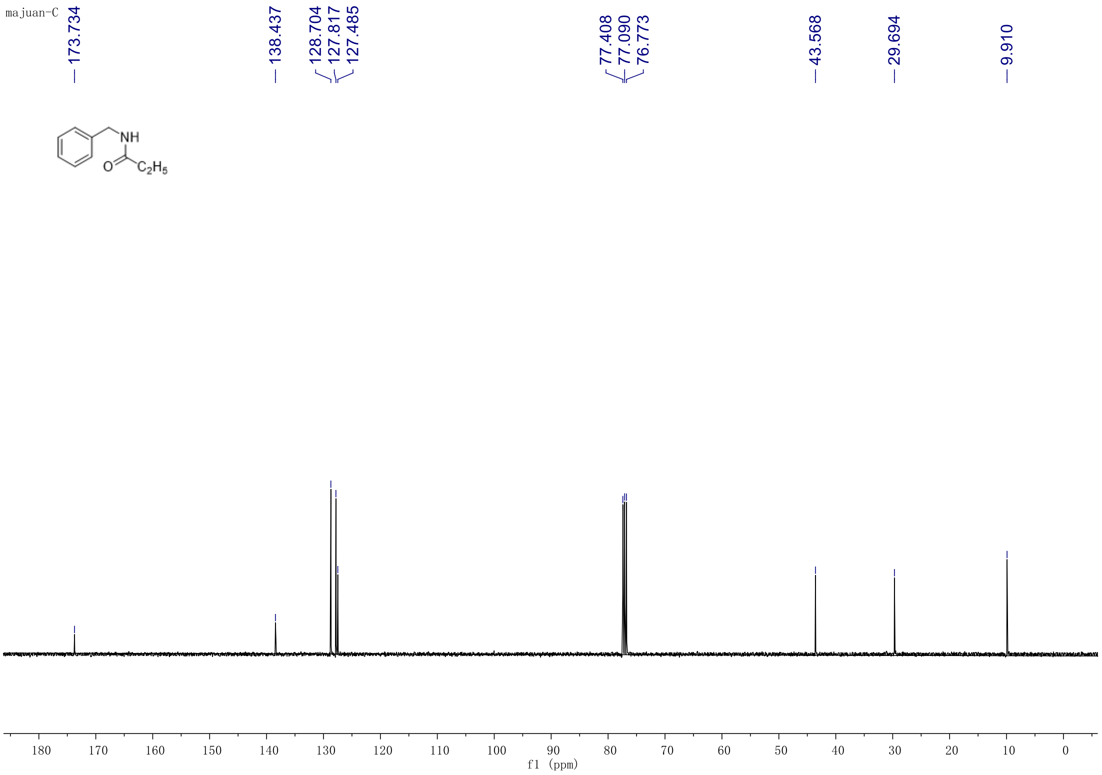

^{13}C NMR spectrum of **2zl** (100 MHz, $CDCl_3$)
